# Testing spatial heterogeneity with stock assessment models

**DOI:** 10.1371/journal.pone.0190791

**Published:** 2018-01-24

**Authors:** Ernesto Jardim, Margit Eero, Alexandra Silva, Clara Ulrich, Lionel Pawlowski, Steven J. Holmes, Leire Ibaibarriaga, José A. A. De Oliveira, Isabel Riveiro, Nekane Alzorriz, Leire Citores, Finlay Scott, Andres Uriarte, Pablo Carrera, Erwan Duhamel, Iago Mosqueira

**Affiliations:** 1 European Commission, Joint Research Centre (JRC), Via Enrico Fermi 2749, 21027 Ispra (VA), Italy; 2 Technical University of Denmark (DTU-AQUA), National Institute of Aquatic Resources, Charlottenlund, Denmark; 3 Instituto Português do Mar e da Atmosfera (IPMA), Av. Dr. Alfredo Magalhães Ramalho, 6, 1449-006 Lisboa, Portugal; 4 IFREMER, Laboratoire de Technologie et Biologie Halieutique, 8 rue François Toullec, 56100 Lorient, France; 5 AZTI-Tecnalia, Marine Research Division. Txatxarramendi Ugartea z/g, 48395 Sukarrieta, Bizkaia, Spain; 6 Centre for Environment, Fisheries and Aquaculture Science (CEFAS), Lowestoft Laboratory, Pakefield Road, Lowestoft, Suffolk NR33 0HT, United Kingdom; 7 Instituto Español de Oceanografía (IEO), Centro Oceanográfico de Vigo, Subida a Radio Faro 50, 36390 Vigo, Spain; 8 AZTI-Tecnalia, Marine Research Division. Herrera kaia Portualdea z/g, 20110 Pasaia, Gipuzkoa, Spain; 9 BCAM, Basque Center for Applied Mathematics, Mazarredo 14, E48009 Bilbao, Basque Country, Spain; Aristotle University of Thessaloniki, GREECE

## Abstract

This paper describes a methodology that combines meta-population theory and stock assessment models to gain insights about spatial heterogeneity of the meta-population in an operational time frame. The methodology was tested with stochastic simulations for different degrees of connectivity between sub-populations and applied to two case studies, North Sea cod (*Gadus morua*) and Northeast Atlantic sardine (*Sardina pilchardus*). Considering that the biological components of a population can be partitioned into discrete spatial units, we extended this idea into a property of additivity of sub-population abundances. If the additivity results hold true for putative sub-populations, then assessment results based on sub-populations will provide information to develop and monitor the implementation of finer scale/local management. The simulation study confirmed that when sub-populations are independent and not too heterogeneous with regards to productivity, the sum of stock assessment model estimates of sub-populations’ SSB is similar to the SSB estimates of the meta-population. It also showed that a strong diffusion process can be detected and that the stronger the connection between SSB and recruitment, the better the diffusion process will be detected. On the other hand it showed that weak to moderate diffusion processes are not easy to identify and large differences between sub-populations productivities may be confounded with weak diffusion processes. The application to North Sea cod and Atlantic sardine exemplified how much insight can be gained. In both cases the results obtained were sufficiently robust to support the regional analysis.

## Introduction

The spatial structure of fish and shellfish stocks is a major issue for fisheries management and assessment of stocks’ status. The large body of literature that addresses this problem goes back as far as the 1950’s [1] and crosses all the major text books on population dynamics [2–5], with a number of recent reviews [6–8].

Amid the panoply of processes that induce spatial structure of some kind in fish populations, spatial heterogeneity [6] is one of the most common, in particular in stocks that span large areas. These stocks are likely to cross different ecosystems with different oceanographic characteristics, food availability, etc. Such conditions are likely to have an impact on biological processes at a local scale, creating sub-populations with potentially distinct dynamics, organized in a network of sub-populations, much like the concept of meta-populations [9].

Under these conditions, assessment models that consider a single homogeneous population may overlook important features of the stock dynamics at a finer spatial scale, which may, in turn, impair management objectives.

Several authors developed and tested stock assessment models that explicitly included spatial dynamics [8, 10–18]. While the importance/relevance of understanding spatial dynamics is widely recognized, these models require a large amount of information and are not easy to fit [12, 19], with several authors reporting little or no advantage of spatially explicit models over closed population models [11–14, 16].

Getting informative data about processes with spatial dimensions and plugging it into stock assessment models is not a trivial task. It requires a large investment both in terms of data collection and model development. In most cases, spatially explicit models require information about individual movement, which may not be possible to obtain. For example, it has been shown [8] that models which include tagging data outperform closed population models in the presence of high population connectivity. However, the authors recognized that tagging information is limited to data-rich species. Furthermore, one must consider that in many cases tagging individuals may not be possible due to species sensitivity, *e*.*g*. small pelagic species.

This paper describes a methodology that combines meta-population theory [6, 9] and stock assessment models to gain insights about spatial heterogeneity of the meta-population in an operational time frame. Starting from a hypothesis of spatial dynamics of a meta-population, the comparison of assessment results of the meta-population and the combined results of its components, allows testing the existence of closed sub-populations which could form the basis of regional management actions. The methodology is tested with stochastic simulations for different degrees of connectivity between sub-populations and applied to two case studies, the stocks of North Sea cod (NS cod, *Gadus morua*) and Northeast Atlantic sardine (NEA sardine, *Sardina pilchardus*).

## Methods

Considering that the biological components of a population can be partitioned into discrete spatial units [6], we extended this idea into a property of additivity of sub-population abundances, such that $N_{iy}=\sum_{j=1}^{m}N_{ijy}$, where *N* is abundance, *i* indexes ages, *j* indexes sub-populations, *y* indexes years and *m* is the number of sub-populations. Furthermore, if each sub-population is closed, in the sense of not having significant migrations across sub-populations, the estimates of *N* obtained from stock assessment models fitted to each sub-population will add up to the estimates obtained from the meta-population fits.

Our assumption is that, if the additivity results hold true for putative sub-populations, then the sub-populations are isolated spatial components of the meta-population. Under this assumption the assessment results based on sub-populations may provide the necessary information to develop and monitor the implementation of finer scale/local management measures, *e*.*g*. setting fishing mortality reductions differentiated locally or monitoring local depletion.

**Fig 1:**
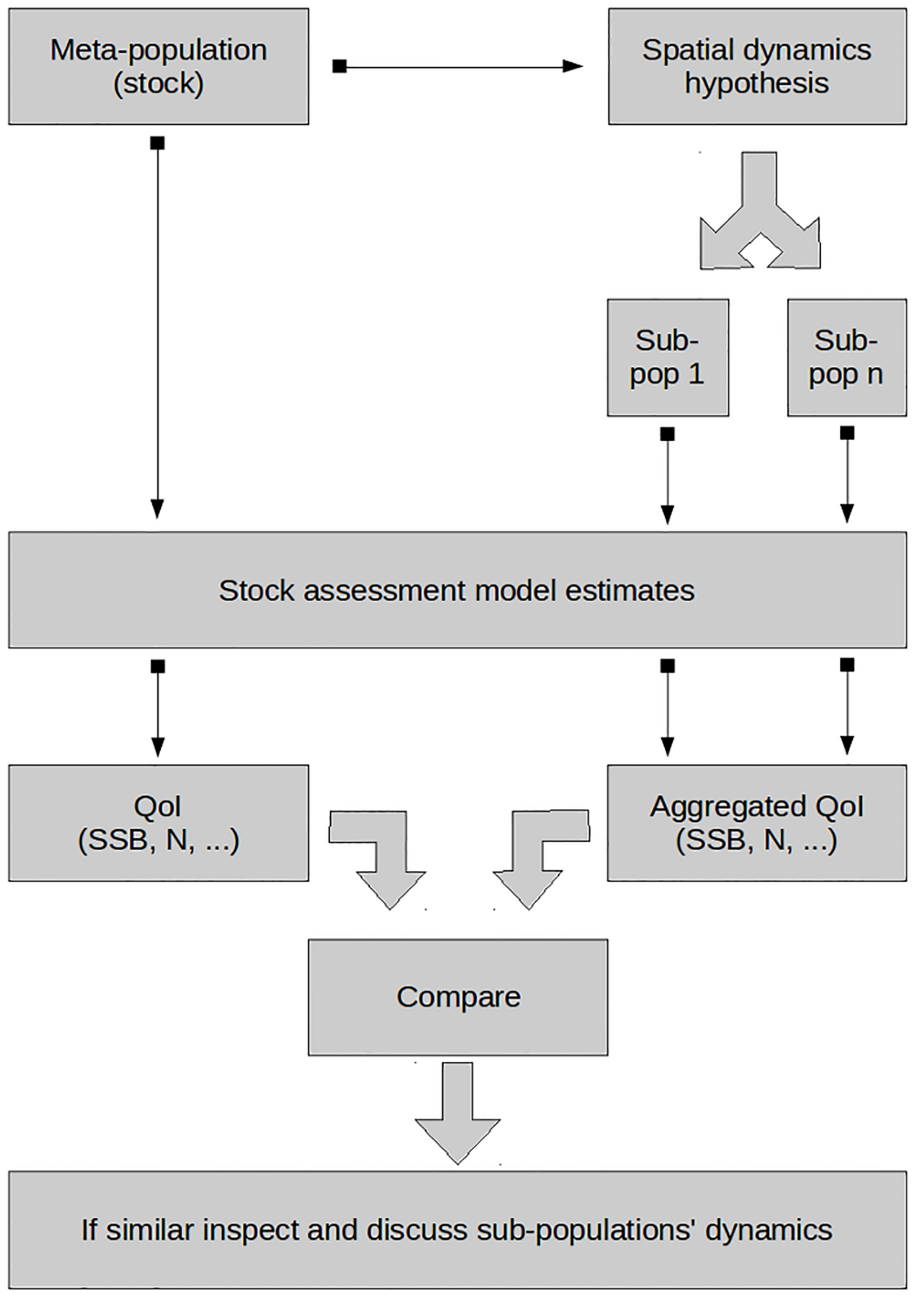
Method’s workflow. From top to bottom, the meta-population is broken down into subpopulations based on a spatial dynamics hypothesis; stock assessment models are fitted and quantities of interest (QoI) derived for the meta-population and the sub-populations; the QoIs of the two scenarios are compared and if considered similar the analysis proceeds with the inspection and discussion of the sub-populations’ dynamics.

Fig 1 depicts the method’s workflow. Starting from a stock for which a hypothesis of spatial dynamics exists, a set of sub-populations are created by reprocessing the raw data and creating stock assessment input data for each sub-population. Afterwards, stock assessments models are fitted to the meta-population and each sub-population, followed by the computation of quantities of interest (QoI), *e*.*g*. SSB, aggregated at the spatial level of the meta-population. The QoI are compared and if the estimates are considered similar, *e*.*g*. by having overlapping confidence intervals, the process continues with the analysis of the sub-population dynamics. Otherwise, the spatial dynamics hypothesis is not supported by the model results.

The methodology was first tested on simulated populations and afterwards applied to the case studies of NS cod and NEA sardine. The simulated populations were loosely based on the biology and fisheries of those stocks, to cover a demersal stock and a small pelagic stock.

All the analysis were carried out using the statistical platform R [20], FLR [21] and a4a [22].

### Simulation study

The simulation study was designed to assess if the methodology suggested was able to distinguish between a meta-population composed of independent sub-populations, from a meta-population composed of connected sub-populations through a diffusion process.

The simulation study was organized into a set of scenarios, each of them containing three operating models (OM):

- **A**: the meta-population, a single stock that spans the full area of distribution;
- **I**: two independent non-overlapping sub-populations that combined cover the same area as A;
- **D**: two non-overlapping sub-populations that combined cover the same area as A, which are connected through a diffusion process.

To generate the OMs, first the two sub-populations of OM I were simulated. Secondly, OM A was created by merging the two OM I sub-populations into a single meta-population, adding population abundances and catches. Finally, the two sub-populations of OM D were simulated by applying a generalized logistic curve to split the OM A population abundance at age into two sub-populations. One of which has a larger fraction of recruits and the other of adults. OM D approximates a situation were the spawner population migrates to a spawning area without showing parental homing effects. Although this OM is not simulated using an explicit spatial model, the two sub-populations share the relevant characteristics to approximate the intended dynamics. The logistic model sets the proportion by age of individuals that moved away from the recruitment region. The model was constrained between six different intervals of diffusion: 0.0 − 1.0, 0.1 − 0.9, 0.2 − 0.8, 0.3 − 0.7, 0.4 − 0.6, 0.5, where the left value refers to the proportion of diffusion applied to the youngest age and the right value to the oldest age (S1 Fig). Note that the last interval, 0.5, simply splits the population abundance, and catches, in two equal parts.

Population’s dynamics were simulated using methods designed by [23]. These methods use life history parameters and fleet selectivity to generate a population and fishery which are consistent with specified reproductivity, growth and mortality. Natural mortality was kept constant across OMs. Two fisheries were used to inform the simulations, NS cod caught by a trawl like fleet (parameters’ details in S1 Table), and Northeast Atlantic sardine caught by a purse seine like fleet (parameters’ details in S2 Table).

For stock recruitment relationships [2] a Beverton and Holt model was used for cod and a Ricker model for sardine. In both cases steepness [24] was set between 0.7 and 0.95 at 0.05 intervals, generating 6 distinct stock recruitment relationships for each population (S1 Fig). A total of 21 unique combinations of stock-recruitment relationships were created.

Each population was projected for 30 years from an unfished status, applying two different fishing histories. In the case of sardine, during the first 12 years fishing mortality was increased linearly from 0 to *F*_*max*_ and kept at that level for the next 18 years, mimicking a situation of optimal exploitation. For cod, during the first 12 years fishing mortality was increased linearly from 0 to 1.5 × *F*_*MSY*_, kept at that level for the next 12 years and linearly reduced back to *F*_*MSY*_ in the following 6 years, mimicking an over-exploitation followed by a recovery process. These patterns do not aim at simulating the history of these two fisheries in particular, but rather general patterns that can be observed in the development of fisheries worldwide.

Uncertainty was added by generating 250 iterations for each population. Drawing log normal independent and identically distributed simulations of recruitment, target fishing mortality (*F*_*max*_ or *F*_*MSY*_), catch numbers-at-age and survey catchability. Survey catchability was set to mimic a research survey, using an exponential decay selectivity with larger selectivities at younger ages, which was used to create an age-structured abundance index required to fit the stock assessment model.

Summarizing, there were 252 scenarios, composed of 21 pairs of stock-recruitment steepness values, 6 diffusion levels, 2 life histories and fleet selectivities (cod and sardine). Each scenario had 3 OMs with 5 populations in total, one meta-population, two independent sub-populations and two connected sub-populations. Each population had 250 iterations.

Having generated the populations for each scenario, the a4a stock assessment framework [22] was used to estimate each population’s abundance and fishing mortality, which were later used to compute relevant statistics for comparison purposes. The a4a framework is based on a flexible statistical catch-at-age stock assessment model [2], which requires as minimum input data catch in numbers-at-age, natural mortality and an abundance index-at-age.

The stock assessment model was set to use a tensor product of thin plate splines to model fishing mortality by age and time, and a regression spline to model survey catchability by age. For more information about splines see [25] and the R package ‘mgcv’.

For each OM, estimates of SSB were derived by:

$$\begin{array}{c} {S\hat{S}B}_{y}=\sum_{i}{\hat{N}}_{iy}*W_{iy}*P_{iy} \end{array}$$ (1)

where $\hat{N}$ represents abundance in numbers at age estimated by the stock assessment model, *W* represents individual mean weight by age, *P* proportion mature by age, and *i* and *y* index ages and years, respectively.

For OM I and D the estimates of population abundance of each sub-population were added before computing SSB:

$$\begin{array}{c} {\hat{N}}_{iy}=\sum_{j=1}^{2}{\hat{N}}_{ijy} \end{array}$$ (2)

where *j* represents sub-populations.

To compare across OMs, the root median square deviation (RMSD) between SSB estimates by OM I or OM D, and OM A was computed:

$$\begin{array}{c} RMSD=\sqrt{median{\left({S\hat{S}B}_{yr}^{x}-{S\hat{S}B}_{yr}^{A}\right)}^{2}} \end{array}$$ (3)

where *r* represents stochastic replicates and *x* the OMs, which may be *I* or *D*.

A strong diffusion process should generate larger differences between the estimates obtained for OM D and OM A, than between OM I and OM A. In fact, the differences between OM I and OM A estimates are expected to be small, as long as the stock assessment fits are unbiased.

### Case studies

For each case study an initial hypothesis of spatial dynamics was used to split the meta-population into sub-populations. The data were disaggregated to the sub-population level to allow fitting stock assessment models. A stock assessment model was configured for each case study taking into account the quality of each fit. The meta-population estimates were compared with the aggregated estimations from the assessment models of the sub-populations. Finally, estimated *N*, *F* and stock-recruit relationships were depicted to show the differences across sub-populations. To test the robustness of results to model structure, a separable fishing mortality model was fitted to the meta-population and sub-populations and the same QoI compared.

Detailed descriptions of data compilation, tests and model fits are provided in the supporting information for cod (S1 File) and sardine (S2 File).

### North Sea cod case study

For stock assessment purposes the International Council for the Exploration of the Sea (ICES) considers NS cod to span ICES areas 4 (North Sea), 3.a.20 (Skagerrak) and 7.d (Eastern Channel). This stock is assessed by ICES on a yearly basis as a single stock.

However, it has long been recognized that the NS cod stock is likely composed of several sub-populations [26–29]. The clearest evidence is for two populations, one inhabiting the north east North Sea and the other shallower waters.

This is supported by both microsatellite and SNP evidence [29–31] and limited movements among life-stages of cod [32, 33]. The reproductive isolation between these subpopulations may have partly arisen through oceanographic retention of the early life-history stages [34]. The other genetic evidence for population separation indicates a separation of the North Sea and Norwegian coastal groups. Tag-recapture studies along the Norwegian coast suggest that coastal cod exhibit a residential behaviour [35]. Scales of juvenile and adult fidelity indicated from non-genetic methods suggest an even more detailed population structuring, with little exchange between the southern and northwest North Sea [36].

These two populations experience widely different hydrogeographic and environmental conditions, with Northern waters being on average colder but more stable environments. These conditions induce important differences in growth patterns [33, 37], maturation schedules [38] and fecundity-size relationships [39].

**Fig 2:**
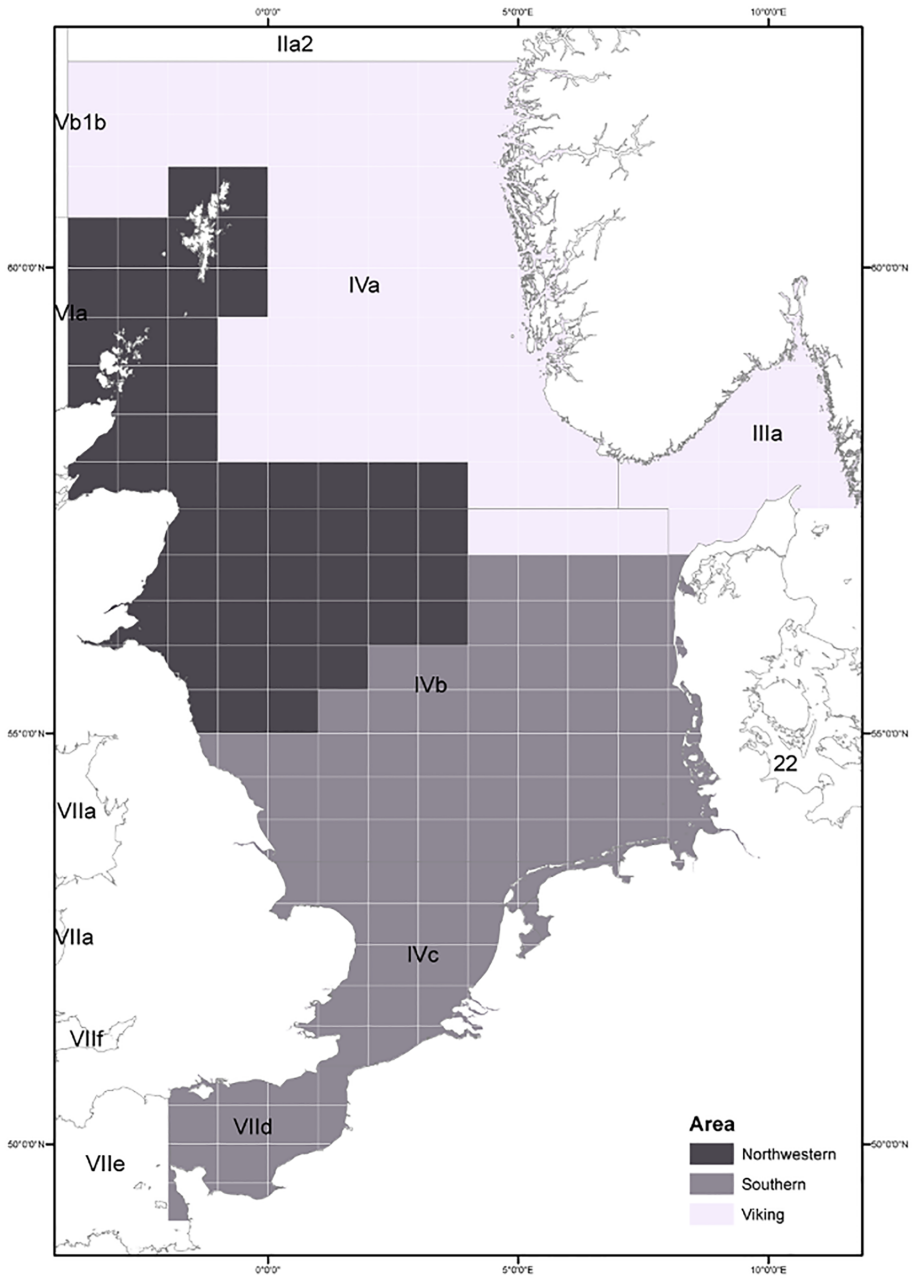
North Sea cod meta-population distribution area and sub-populations. The areas used to split the meta-population into putative sub-populations: Southern (mid gray), Northwestern (dark gray) and Viking (light gray).

The analyses in this paper are based on the hypothesis that there are three sub-populations of cod in the North Sea: Southern, Northwestern and Viking (Fig 2). The delineation of sub-areas was defined based on a synthesis of available information on population structure conducted for the NS cod assessment benchmark workshop in ICES [40].

It should be noted that the exact boundaries for distribution of subpopulations are not clear and there is uncertainty in the extent of mixing of sub-populations across the sub-areas we have defined for the purpose of this study. The defined sub-areas are similar to those used in earlier analyses [41].

Input data for area-based analyses of stock dynamics and fishing pressure were compiled by sub-areas for the years 2003–2013. Landings in weight by sub-areas were obtained from the European Commission’s Scientific, Technical and Economic Committee for Fisheries (STECF) database (http://datacollection.jrc.ec.europa.eu/dd/effort). Total North Sea cod discards in weight were distributed across sub-areas based on the relative distribution of cod’s landings in those areas. Spatial data on age structure of landings and discards were available from England & Wales and Denmark.

International Bottom Trawl Survey (IBTS) observations in quarter 1 (Q1) and quarter 3 (Q3) were used to derive catch per unit of effort indices by sub-areas, using the same methodology as applied for the entire stock. Area-specific mean weights at age in the stock were estimated from IBTS Q1 data. For natural mortality and maturity at age, the values used in the ICES assessment were applied for all sub-areas.

### Northeast Atlantic sardine case study

For stock assessment purposes ICES considers three stocks of sardine (*Sardina pilchardus*) in the Northeast Atlantic: the Bay of Biscay stock distributed in ICES divisions 8.a, b, d (North and Central Bay of Biscay), the Iberian Peninsula stock distributed in ICES divisions 8.c (South Bay of Biscay) and 9.a (South Galicia, Portuguese waters and Gulf of Cadiz), and the Celtic Seas-English Channel stock (ICES sub-area 7), recently considered to be a separate stock [42]. Due to lack of information the Celtic Seas-English Channel stock was not taken into account in this study.

Genetic studies [43] provide no sign of reproductive isolation among sardine populations across European Atlantic waters. However, a pattern of isolation by distance, such that far apart populations are differentiated, is evident in various studies [44]. Regional differences in body morphology, otolith shape, growth, maturation, spawning seasonality and demography suggest there might be several sardine sub-populations forming a meta-population within the area [45, 46].

**Fig 3:**
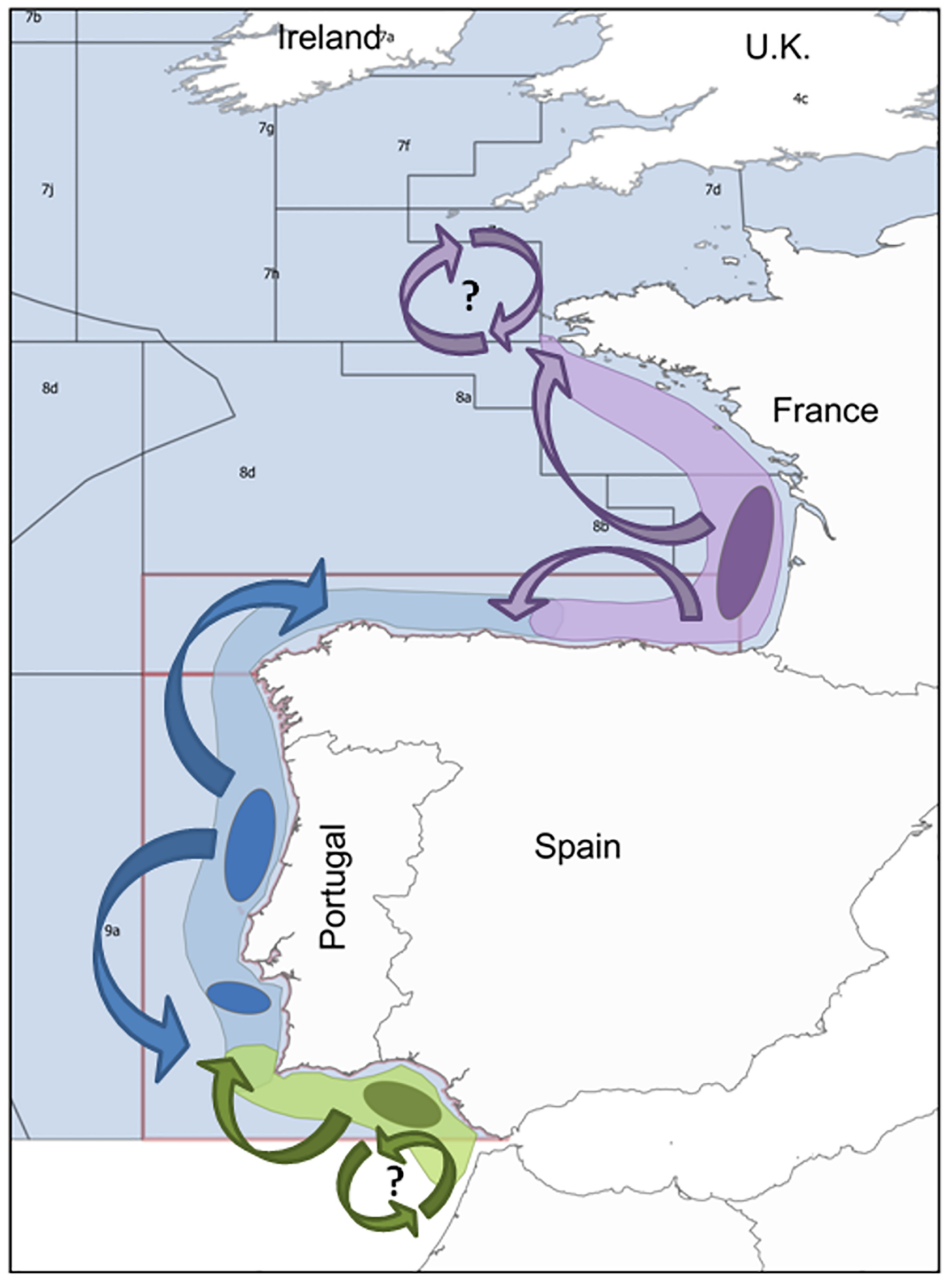
Northeast Atlantic sardine meta-population distribution area and sub-populations. The areas used to split the meta-population into putative sub-populations: Bay of Biscay sub-population (purple), Northwest sub-population (blue) and South sub-population (green). Ellipses refer to persistent recruitment hot spots. Arrows refer to potential migration paths between sub-populations.

The analyses in this paper are based on a conceptual model that hypothesizes the existence of three sardine sub-populations (Fig 3): a Bay of Biscay sub-population (divisions 8.a, b), a Northwest sub-population including from the Cantabrian Sea to southwestern Portugal (divisions 8.c and 9.a) and a South sub-population, including the southern Portuguese waters and the Gulf of Cadiz (division 9.a) [46, 47]. The conceptual model assumes each sub-population is associated with a separate localized and persistent recruitment hot spot (ellipses in Fig 3): Bay of Biscay with southern Brittany and mid-Bay of Biscay, Northwest with north-western Portugal and South with the Gulf of Cadiz [48, 49]. Furthermore, it assumes there’s mixing between the two main stocks and evidence of sub-structure within each of them [50, 51], represented by arrows in Fig 3.

Mixing between sub-populations at the border areas is likely given the pelagic behaviour of juveniles and adults, the continuity of the spawning area and the overlap of the spawning period [52, 53]. For example, a study simulating egg and larvae dispersal indicated an exchange rate of 5% between neighbour ICES-subdivisions [54]. At the adult phase, a multi-area Bayesian assessment model estimated that 19% of the biomass distributed in east Cantabria (8.c-east) derived from juveniles recruited in south Biscay (8.b), the border areas of the Northwest and Biscay sub-populations respectively [55]. Emigration of adults into the Bay of Biscay was lower but also likely. However, if the whole Iberian Peninsula was considered, immigration accounted for 1–4% of the stock, supporting the hypothesis of independent dynamics between the two sub-populations despite a degree of mixing. Low connectivity between the two sub-populations is further corroborated by asynchronous year-class strength and lack of massive migration of strong year-classes recruited in the Bay of Biscay towards the southern sub-populations which have decreased severely in recent years [42]. With respect to the Northwest and South sub-populations, differences in body and otolith shape, life-history properties and cohort dynamics, all point to some differentiation, mainly with respect to the Gulf of Cadiz. In terms of otolith shape [56], growth and maturation [57, 58] sardine distributed in the Gulf of Cadiz appear to be closer to sardine in southwestern Mediterranean than to those in western Portugal. Nevertheless, otolith chemistry suggested that a strong cohort (2004) dispersed from northwest Portugal and made up the bulk of the adults of that cohort in the southern areas [59]

The three putative sub-populations are possibly connected by larval transport and juvenile/adult dispersal/migration but typical seasonal spawning migrations and homing behavior do not seem to take place across the area [49, 52, 60]. It is also likely that the extent of connectivity changes over time depending on local recruitment strength and environmental conditions.

Input data for the analyses were fisheries and survey data in the period 2000–2015, disaggregated into three geographical areas corresponding to the Bay of Biscay, Northwest and South sub-populations. Fisheries data used were: biomass, numbers-at-age and mean weight-at-age in the catches per year. Survey data were abundance-at-age from annual French, Spanish and Portuguese spring acoustic surveys, and either an index of biomass from a triennial Daily Egg Production Method (DEPM) survey (Northwest and South areas) or the total abundance of eggs from an annual DEPM survey (Bay of Biscay) [61].

In the case of sardine the reconstruction of the time series for each sub-population was more challenging due to differences in sampling plans between the Iberian region and Bay of Biscay.

## Results

### Simulation study

**Fig 4:**
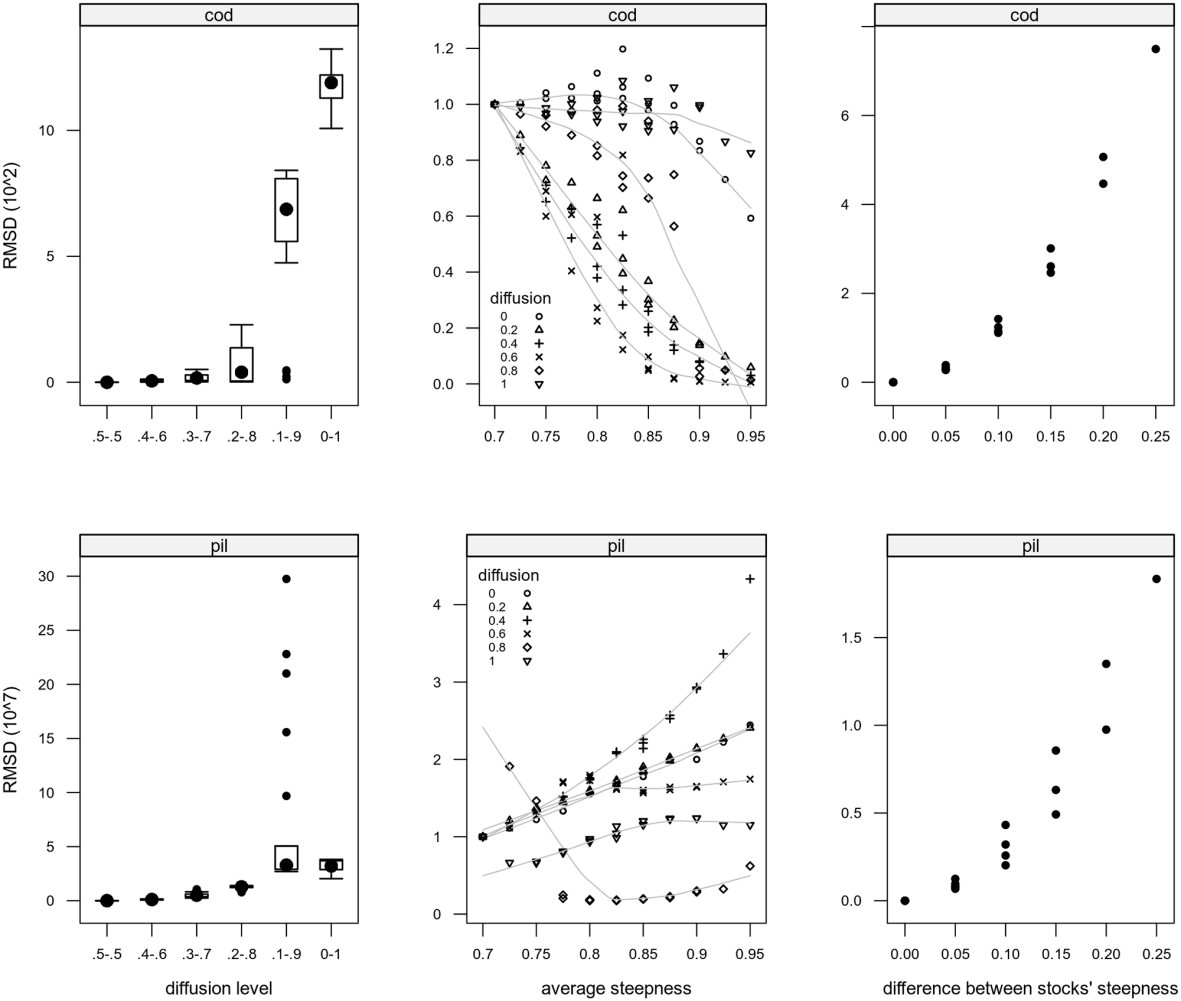
Simulation study results based on the spawning stock biomass (SSB) root median square deviation (RMSD, Eq 3). The top row shows results for the case based on North Sea cod dynamics, the bottom row for the Northeast Atlantic sardine. The left column refers to RMSD as a function of the diffusion level and the central column as a function of stock’s productivity (average steepness), for dependent sub-populations (OM D). The light gray lines are smoothers fitted to each diffusion level to help visualization. These results are presented relative to the 0.7 average steepness and for each diffusion level (legend in the plot). Levels of 0.5 − 0.5 refer to a weak diffusion, where the population has the same age distribution in both sub-populations, while a value of 0.0 − 1.0 refers to the strongest diffusion process, where all recruits are in one area and all adults in another area. The right column shows RMSD as a function of productivity differences between independent sub-populations (OM I).

Fig 4 shows the results of the simulation study (SSB’s RMSD as defined by Eq 3). The top panels show results for the case study based on NS cod dynamics, the bottom panels for the NEA sardine. The left column refers to RMSD as a function of the diffusion level. The central column refers to RMSD as a function of stock’s productivity (average steepness). These results are presented relative to the 0.7 average steepness. Both depict results for dependent sub-populations (OM D). The right column shows RMSD as a function of productivity differences between independent sub-populations (OM I).

For diffusion levels a value of 0.5 − 0.5 refers to a weak diffusion where each sub-population has the same age distribution, while a value of 0.0 − 1.0 refers to the strongest diffusion process, where most recruits are in one area and adults in the other area.

The left panels of Fig 4 show that the stronger the diffusion process, the larger RMSD will be. However, diffusion processes below average (*i*.*e*. < 0.4) in our simulations show a small RMSD when compared with diffusion processes above average. These results show that our methodology will be able to identify situations where the sub-populations have strong diffusion processes but may not be very precise in cases where the spatial heterogeneity in age distribution is small to moderate.

The central panels of Fig 4 depict the relationship between RMSD and stock-recruitment steepness across diffusion levels. For cod (upper central panel) it shows a decrease in RMSD as average steepness increases. While for sardine (bottom central panel) the opposite happens, RMSD increases with increasing average steepnesses. Note that in the case of a Beverton and Holt curve, as used for cod, high steepnesses represent relationships with little structure, with the extreme situation of recruitment independent of SSB if *s* ≈ 1. For the Ricker curve, used for sardine, larger steepness generates curves that generally show larger changes in recruitment at smaller SSBs, particularly in the right side of the curve. In conclusion, when the stock-recruitment relationship reflects a strong link between SSB and recruitment, our method detects the diffusion process better.

The right panels of Fig 4 show RMSD as a function of the difference between steepnesses of the two independent sub-populations used to generate the meta-population. RMSD increases as the difference between two stock’s steepnesses becomes larger, although by very small amounts as can be seen from inspecting the scales of the y-axis across rows. This effect is minor when compared with the effects of diffusion levels and stock’s productivity (average steepness) across connected sub-populations. Nevertheless, at low diffusion levels and/or weak stock-recruitment relationships, this effect may be confounded with the previous ones and mask the relationship between the sub-populations and meta-population.

In summary the simulation study showed that differences between the SSB of the meta population and the aggregated SSB of the sub-populations, arise mainly from diffusion processes as modified by the strength of the stock-recruitment relationships. The largest RMSD values are caused by strong diffusion processes coupled with strong stock-recruitment relationships.

Deriving thresholds from the simulations which can be applied to real case studies was not possible, due to the variability of the processes. Nevertheless, the simulation study identified factors that may be relevant when analyzing real world results and showed that instances of small to moderate levels of diffusion result in low RMSD. In other words, the more similar the indicators from OM A and OM D are, the higher the likelihood that sub-populations are not connected.

### North Sea cod case study

**Fig 5:**
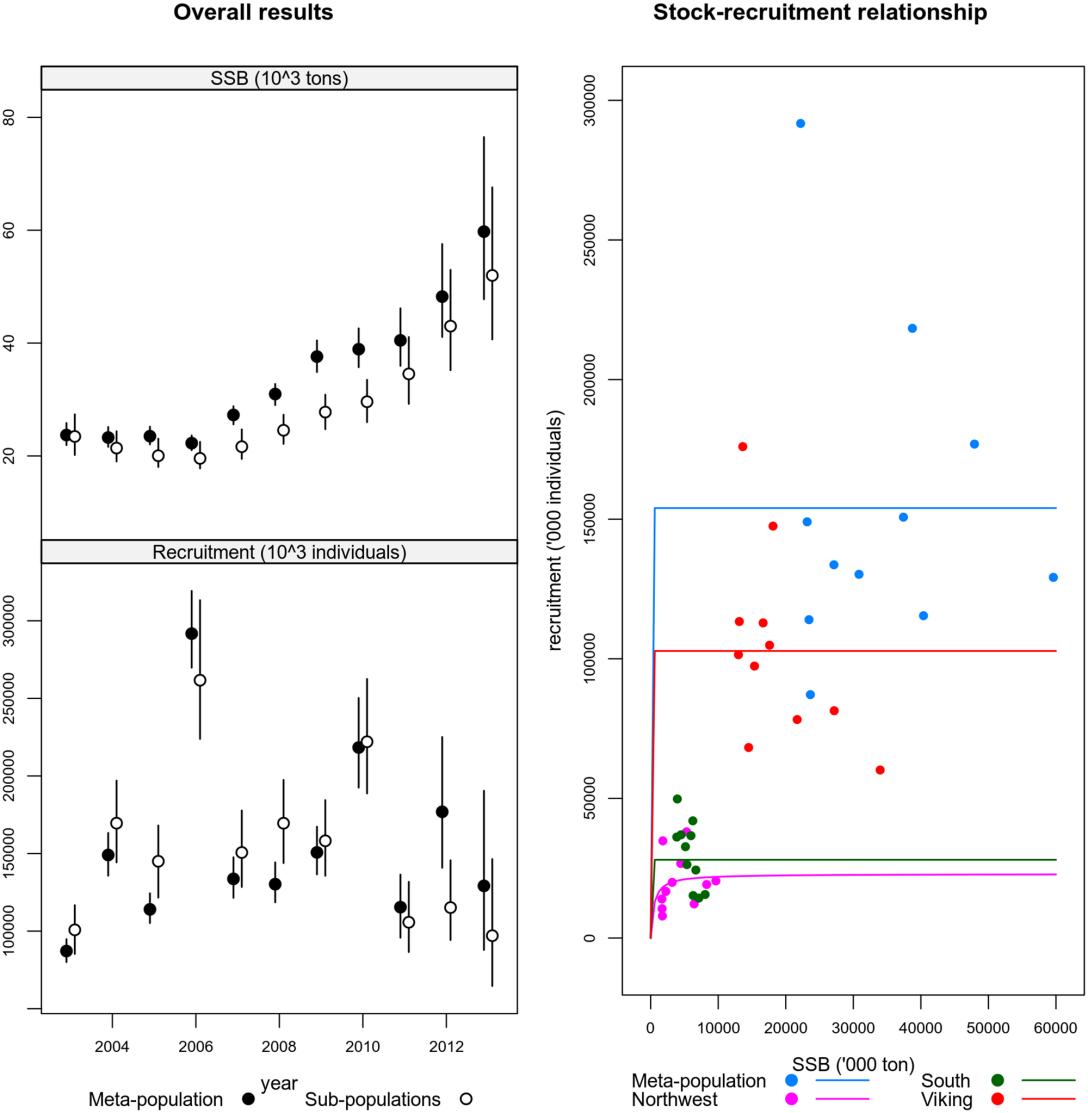
Estimates of spawning stock biomass (*SSB*) for North Sea cod. The left top figure refers to spawning stock biomass and the left bottom to recruitment. In both cases points show the median values and vertical lines represent 95% confidence intervals. The right panel shows the stock recruitment plot and model fits, to the meta-population and each sub-population.

Fig 5 (top left panel) presents SSB estimates (Eq 1) based on the meta population assessment and the aggregation of sub-populations’ assessments. The aggregation is done using Eq 2. Yearly estimates are similar, showing large areas of overlap between confidence intervals, with the exception of the period 2007 to 2010, albeit still sharing a similar time pattern. The differences found can result from the assumptions made to build the sub-populations datasets, namely the allocation of discards to each sub-population, and/or overlap of sub-populations in the boundaries of the spatial distribution. A full review of the data, which is outside the scope of this paper, would be needed to fully address this issue.

The bottom left panel of Fig 5 presents recruitment estimates for the same cases. With the exceptions of 2008 and 2012, all other estimates show overlapping confidence intervals. As expected in statistical catch at age models the most recent year in the assessment is the poorest estimated, showing large confidence intervals.

These results support the assumptions of additivity across sub-populations when breaking down the meta-population into discrete areas, which might indicate that the sub-populations are not overlapping.

The Beverton and Holt stock-recruitment model fits for the meta population and for each sub-population are presented in Fig 5 (right panel). The model fit is very poor and defaults to a mean recruitment model, with the exception of the Northwest area. The figure shows that the Viking area is the largest contributor to recruitment mainly due to large amounts of SSB.

**Fig 6:**
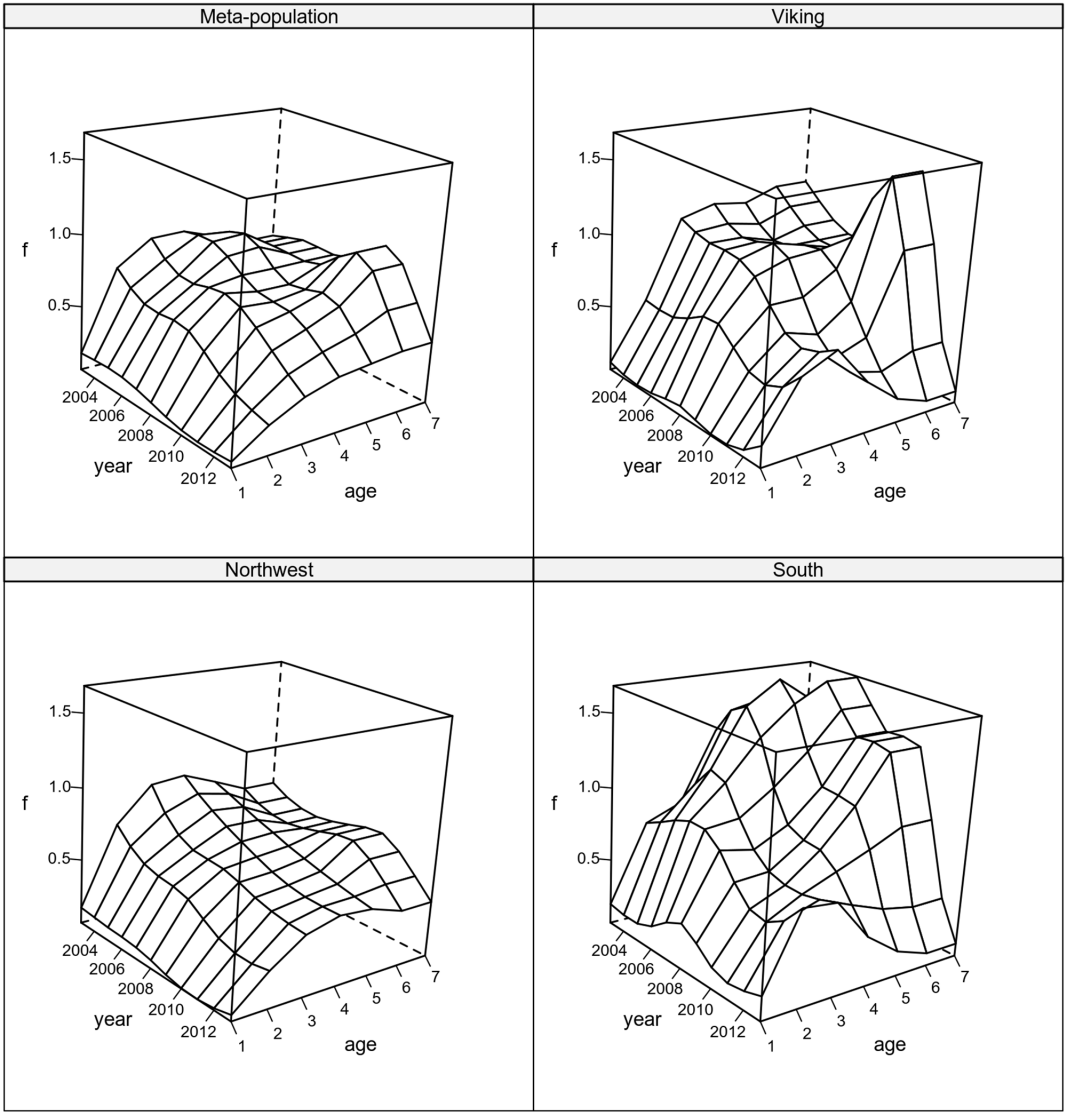
Fishing mortality surface. Fitted to the North Sea cod meta-population and each of the three sub-populations.

Fig 6 presents the fishing mortality estimates for the meta population and for each sub-population. The meta-population fishing mortality shows a general decrease in recent years. Analysing the results by age shows an increase in exploitation of older ages until 2010, followed by a large drop afterwards. Inspecting the sub-populations fishing mortality it can be seen that the Viking and South areas have a larger F on the older ages, which since 2010 decreased sharply, while mortality in the younger ages remained stable. The Northwest area shows a pattern more similar to the meta-population, with a continuous decrease in F for intermediate ages. The inspection of sub-populations shows that the 2010 shift in F at older ages for the meta-population mainly resulted from changes in the Viking and South areas.

### Northeast Atlantic sardine case study

**Fig 7:**
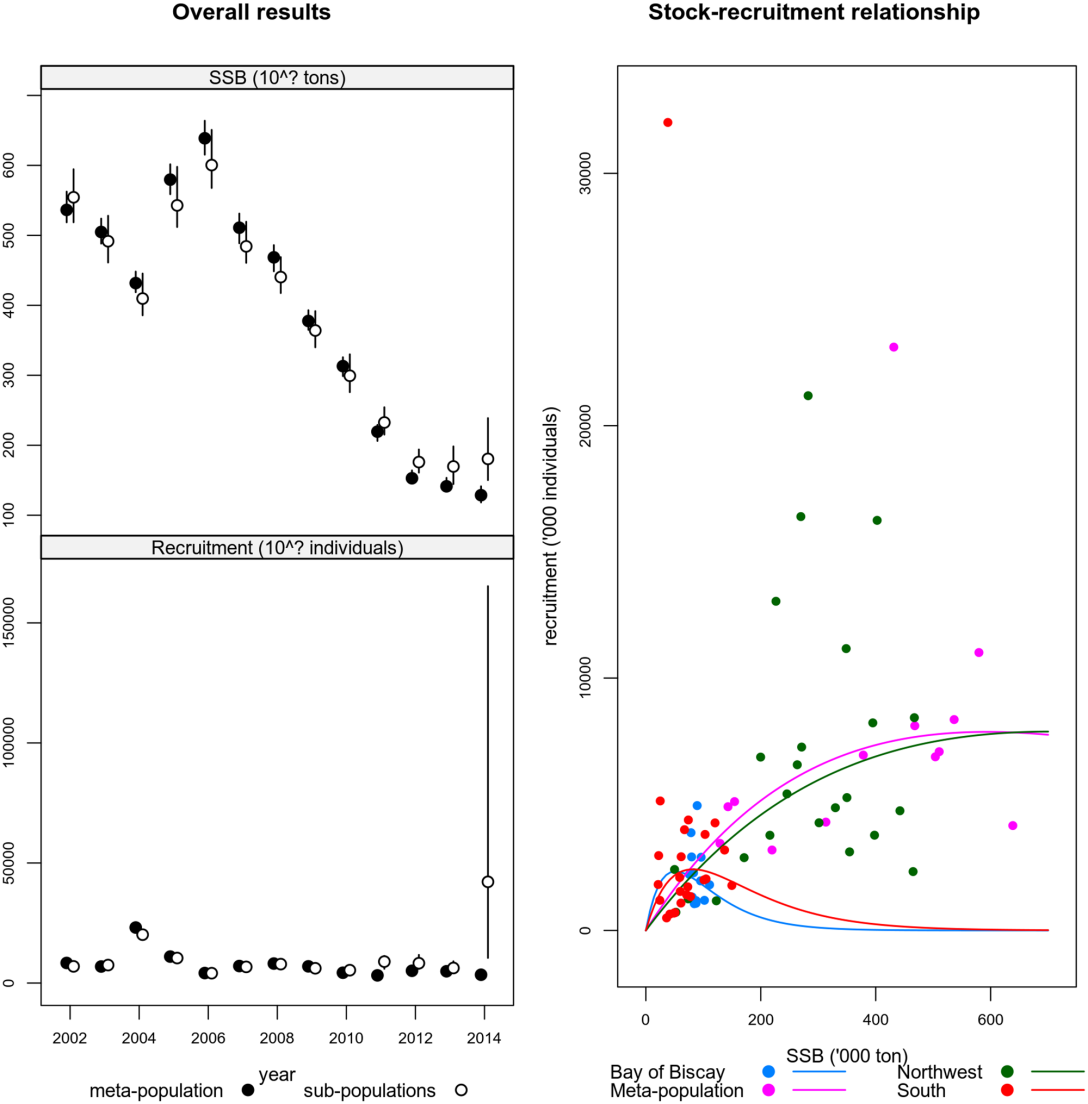
Estimates of spawning stock biomass (*SSB*) for the Northeast Atlantic sardine. The left top figure refers to spawning stock biomass and the left bottom to recruitment. In both cases points show the median values and vertical lines represent 95% confidence intervals. The right panel shows the stock recruitment plot and model fits, to the meta-population and each sub-population.

Fig 7 (top left panel) presents the same information as the previous section for NEA sardine. Yearly estimates are very similar, showing large areas of overlap between confidence intervals. Comparing to cod, sardine shows more consistent results between the two estimates. In 2013 and 2014, there’s a divergence between the two estimates, with the meta-population showing a continuous downwards trend while the aggregated SSB from the sub-populations’ shows a shift upwards. These outcomes may reflect assessment uncertainty or confounding between productivity and diffusion, as shown through the simulations.

The bottom left panel of Fig 7 presents recruitment estimates for the same cases. In general all estimates overlap, with the exception of 2014. As with cod, the results support the assumption of additivity across sub-populations and the existence of sub-population dynamics.

Fig 7 (right panel) presents the Ricker stock-recruitment model estimates for the meta population and for each sub-population. The S/R analysis shows that the Northwest area is the largest contributor to recruitment mainly due to large amounts of SSB. Productivity seems very different though, with the sub-populations of the Bay of Biscay and South showing a higher steepness and the Northwestern a shallower curve. This could mean that those sub-populations are more resilient to low population sizes than the Northwest.

**Fig 8:**
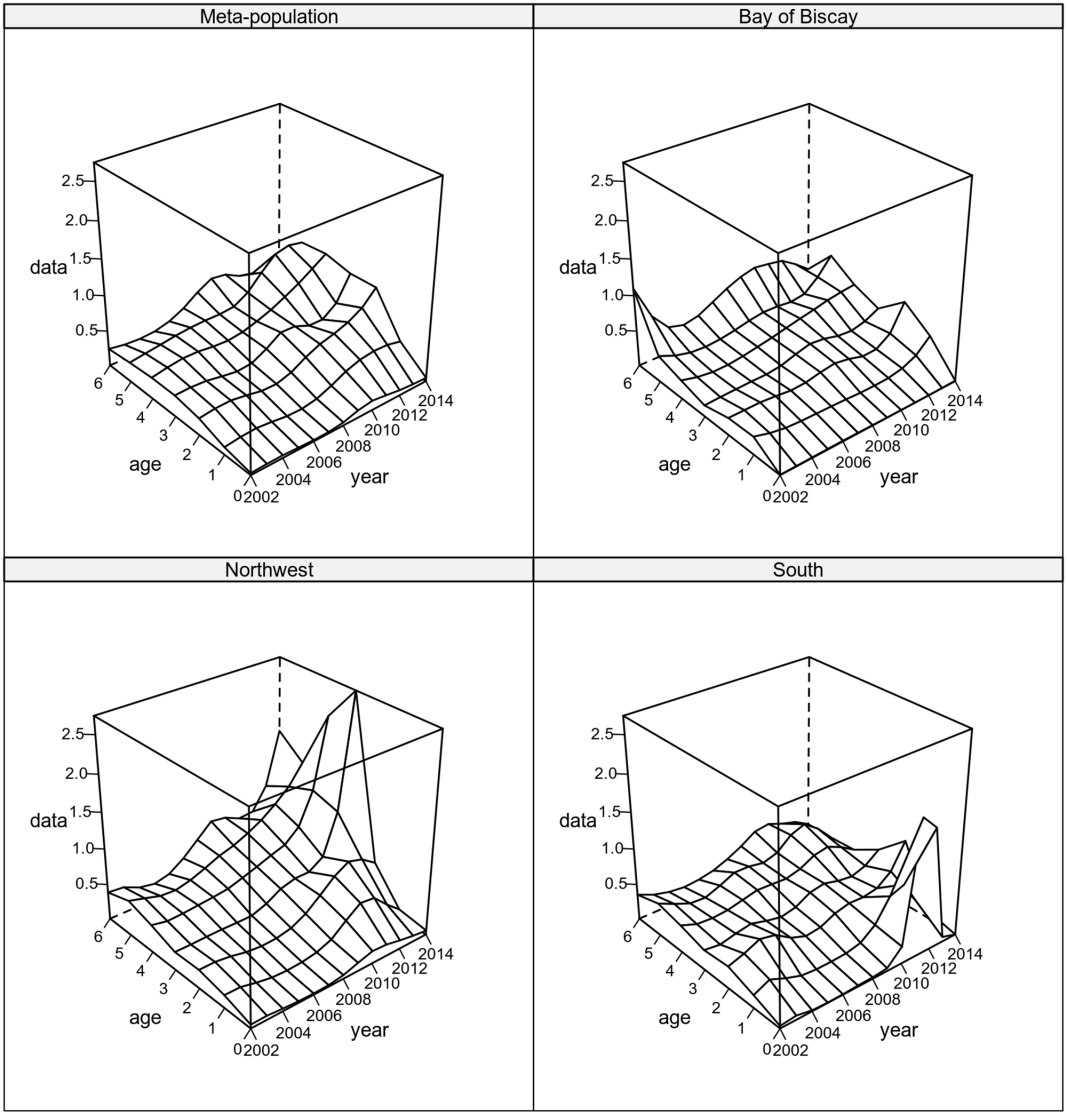
Fishing mortality surface. Fitted to the Iberian sardine meta-population and each of the three sub-populations.

Fig 8 presents the fishing mortality estimates for the meta population and for each sub-population. The overall fishing mortality shows a general increase and a dome shape, centered on ages 2 to 5. Sub-populations’ fishing mortality show an increase in recent years in the Bay of Biscay sub-population and the Northwest sub-population, with a very steep increase in F at ages 3 and 4 in the most recent years. The South sub-population shows a recent decrease in F except at age 0 in 2012 and 2013, when a peak in F was estimated.

Sub-populations’ estimates tend to show larger variances than the meta-population’s estimates (Figs 5 and 7). This result is expected due to the number of parameters which are estimated to construct both statistics. The meta-population estimates use one single model fit, while the sub-populations’ use 3 model fits.

## Discussion

The work presented in this paper shows that meta-population theory [6, 9] and stock assessment models can be combined in an operational way to study spatial heterogeneity, allowing the development of regional management actions and reducing the risks of local depletion.

The rationale of the methodology is to compare the assessment results of a meta-population with the combined results of its components. If the sub-populations are closed—or near closed—or if a zero sum migration exists, the results should be similar. If the sub-populations have a strong connectivity, the stock assessment model fits to each sub-population will be misspecified and the comparison will show large differences between the two cases.

Note that in a situation where all the sub-populations are isolated, and, as such, don’t have any connectivity amongst them, the meta-population doesn’t exist, since meta-population theory assumes some level of connectivity across sub-populations exists. Nevertheless, the forms of connectivity can be very diverse [9] and/or happen at scales that may not be captured by a stock assessment model, *e*.*g*. larvae drift. Our method doesn’t have the power to distinguish such cases. It assumes that if the sub-populations are lightly connected, one can relax the closed population assumption and use stock assessment models. It’s a pragmatic assumption which allows the analyst to focus on the sub-populations and investigate their properties in the context of fisheries management.

Some characteristics of the sub-populations may not be relevant for determining optimum fisheries exploitation. In short, the relevant traits are those related with productivity and survivability. Those are the characteristics that will differentiate sub-populations with relation to fishing impacts and will require adjusted management actions. If a population is less productive due to environmental factors, the exploitation of that sub-population has to be reduced/adjusted to that fact, *e*.*g*. by reducing fishing effort in the sub-population spatial domain in comparison to other areas. On the other hand, if the sub-populations structure is related with *e*.*g*. morphologic diversity traits which don’t impact productivity and/or survivability, the sub-populations structure may not be relevant in this context. However, even when a single harvest strategy is appropriate across sub-populations such sub-population structure is still something of which managers should be aware, to prevent unsustainable fishing pressure on any given sub-population. The limited exchange of individuals across sub-population boundaries implies limited re-colonisation should a sub-population become depleted.

The initial hypothesis of sub-population spatial dynamics must be based on information sources designed for such studies, like morphometrics, genetics, tagging, etc. so that the potential existence of sub-populations are supported by geo-physical and ecological conditions. Using stock assessment model fits to develop meta-population hypotheses can generate spurious results due to the large uncertainty stock assessment results may have [62].

The simulation study confirmed that when sub-populations are independent and not too heterogeneous with regards to productivity, the sum of stock assessment model estimates of sub-populations’ SSB is similar to the SSB of the meta-population. It also showed that a strong diffusion process (age dependent migration) can be detected, presenting SSB estimates that differ between the aggregation over sub-populations and the meta-population. Furthermore, the simulation results showed that the stronger the connection between SSB and recruitment, the better the diffusion process will be detected.

On the other hand, although the simulations showed that stock assessment models’ fits were able to support the case for putative sub-populations, they also showed that (i) weak to moderate diffusion processes are not easy to identify, and (ii) large differences between sub-populations productivities may be confounded with weak diffusion processes.

An important requirement for these studies is reprocessing the input data for stock assessment. Scientists have to go back to their samples and (i) estimate total weights caught in each sub-population, (ii) estimate age and/or length structure of the catches, split by discards and landings if relevant, (iii) reprocess biological samples to estimate maturity matrices and growth models or age-length-keys, and (iv) estimate abundance indices adjusted to the sub-populations. This is not a minor task and should be considered carefully when deciding to split a stock into sub-populations. In the case studies presented, reprocessing of data required a new set of assumptions to be made and trimming the initial periods of the time series of the sub-populations, loosing part of the information available. Shorter time series render stock assessment model fits more unstable and less precise. Key parameters like stock’s productivity or fleet’s exploitation patterns, will be worse estimated, potentially having a negative impact on scientific advice.

Another important factor to have in mind when applying our methodology is the comparability across stock assessment models fitted to each stock (meta-population and sub-populations). Modeling frameworks like SS3 [63] or a4a [22] allow the analyst to choose across a large number of model structures, which may have an impact on the results. Comparing model fits require these to be as similar as possible to avoid confounding the model structure with model estimates. An extreme example would be to fit a biomass dynamic model to one sub-population and compare its results with an age-based model in another sub-population. Our suggestion is to test the robustness of results using a sensitivity analysis on model structure. For the case studies presented here, a separable model for fishing mortality was used as an alternative (see support material for cod in S1 File and sardine in S2 File). The perspective about spatial dynamics didn’t change when considering the three sub-populations hypothesis for both stocks, which is reassuring regarding the robustness of our results.

The evaluation of similarity across scenarios was another important subject. In the case studies we relied on the visual inspection of the overlap between confidence intervals. The computation of confidence intervals for the QoI over sub-populations assumes the estimates are independent. In our opinion it doesn’t constitute a major problem since the methodology was designed on the same principles. Developing more formal statistics, although possible, was outside the scope of our work. Nevertheless, considering the large uncertainty associated with stock assessment models, a fair degree of flexibility has to be given to the analyst.

The application to North Sea cod and Atlantic sardine exemplified how much insight can be gained by using the methodology proposed here. If the initial hypothesis of sub-population structure is supported, estimates of population abundance, fishing mortality, exploitation pattern and stock-recruitment dynamics could be derived for each sub-population. In both cases the results obtained were sufficiently robust to allow the regional analysis as postulated in Figs 2 and 3, respectively. The information clearly showed differences in exploitation patterns. Some areas were more focused in exploiting younger ages, areas South for Sardine and Northwest for cod. While others were more focused in older ages, Bay of Biscay and Northwest for sardine, and Viking and South for cod. This information allows the development of management measures targeting the characteristics of the fleet and sub-population dynamics in each region. In the long run such differentiation will allow a better management of the stock and/or a quicker recovery of over-exploited populations.

In our case studies, as it would be expected in other cases, the boundaries between sub-populations have some level of uncertainty. It won’t be easy to establish rigid geographical boundaries, unless clear physical barriers exist. This fluidity, which most likely will show some temporal dynamics, has to be taken into account by the analysis, since the results will be conditional on it. Our suggestion is to repeat the analysis some years later when more data will be available. For the case studies presented here, further work like recovering the full time series of data, would be desirable and could provide more insights about each stock’s spatial dynamics.

Our approach is not as sophisticated as full spatial models [7, 18], it resembles more a lattice approach [64], where the lattices are considered to be independent. Such an assumption is obviously a simplification but it allows the analysts to study the spatial dynamics of the stocks in an operationally useful time frame, constituting a complementary framework for providing scientific advice to fisheries management which takes into account the spatial dynamics of the stock. This approach therefore provides a powerful tool to build a regional perspective on the dynamics of stocks and their exploitation, and explore the impact of management options.

## Supporting information

S1 Fig Simulation study conditioning. Top figures show the stock recruitment relationships used in the simulation study. The Beverton and Holt curve was used for the cod like stock (right panel), while a Ricker curve was used for the sardine stock (left panel). The different lines refer to values of steepness of the stock recruitment relationship. Bottom figures show the generalized logistic curve used to simulate the diffusion process as the percentage by age of total population corresponding to sub-populations. A value of 0.5 − 0.5 refers to the flat line which means the population has the same age distribution in both areas. A value of 0.0 − 1.0 refers to a logistic between 0 and 1 which imposes the strongest diffusion process, where all recruits are in one area and all adults in another area.
(TIF)

S1 Table Parameters used in the population and fishery simulation loosely based on North Sea cod (*Gadus morua*). (PDF)

S2 Table Parameters used in the population and fishery simulation loosely based on Northeast Atlantic sardine *Sardina pilchardus*). (PDF)

S1 File North Sea cod—Model sensitivity analysis. (PDF)

S2 File Northeast Atlantic sardine—Model sensitivity analysis. (PDF)
