# Supplementary figures and images for: Testing spatial heterogeneity with stock assessment models
Source: PLoS One. 2018 Jan 24;13(1):e0190791. doi: 10.1371/journal.pone.0190791 (PMC5783371; doi:10.1371/journal.pone.0190791)

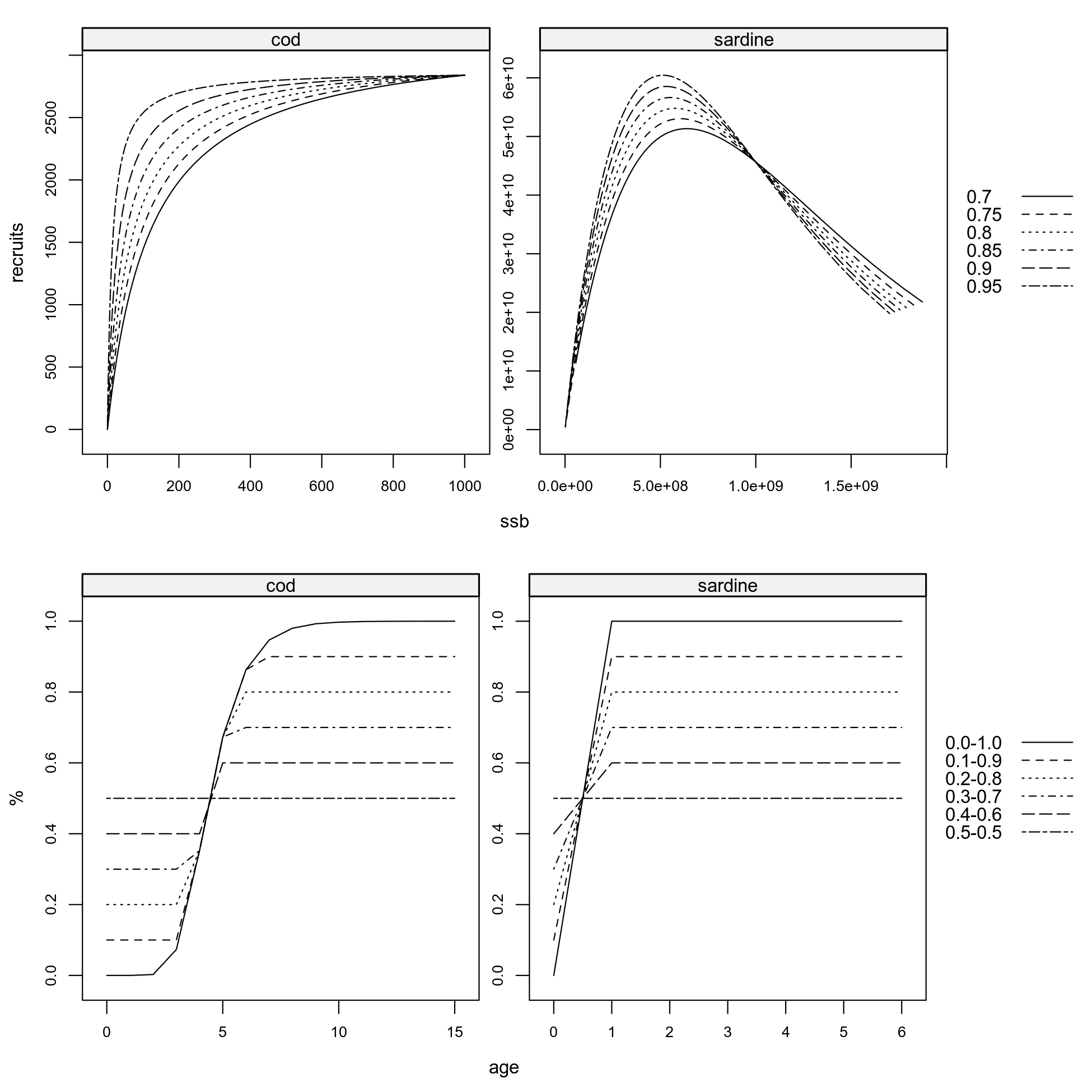

Supplement: S1 Fig — Top figures show the stock recruitment relationships used in the simulation study. The Beverton and Holt curve was used for the cod like stock (right panel), while a Ricker curve was used for the sardine stock (left panel). The different lines refer to values of steepness of the stock recruitment relationship. Bottom figures show the generalized logistic curve used to simulate the diffusion process as the percentage by age of total population corresponding to sub-populations. A value of 0.5 − 0.5 refers to the flat line which means the population has the same age distribution in both areas. A value of 0.0 − 1.0 refers to a logistic between 0 and 1 which imposes the strongest diffusion process, where all recruits are in one area and all adults in another area. (TIF) [file pone.0190791.s001.tif]
