# Supplementary material for: Testing spatial heterogeneity with stock assessment models
Source: PLoS One. 2018 Jan 24;13(1):e0190791. doi: 10.1371/journal.pone.0190791 (PMC5783371; doi:10.1371/journal.pone.0190791)
Supplement: S1 Table — (PDF) [file pone.0190791.s002.pdf]

S1 table Parameters used in the population and fishery simulation loosely based on North Sea cod (*Gadus morua*).

subject	process	model	parameter	value
population	growth	von Bertalanffy	L_∞	$140cm$
			k	0.125
			t_0	0
	natural mortality	Gislason[1]		
	length-weight		a	0.0006
			b	3
	reproduction	logistic	a_{50}	3.5
	stock-recruitment	beverton and holt[2]	virgin biomass (SSB_0)	1000
			steepness (s)	$\{0.7, 0.75, 0.8, 0.85, 0.9, 0.95\}$
fishery	selectivity	double normal	σ	2
		(flat top)	σ	100
			L_{50}	2
error	fishing mortality	log-normal	cv	0.4
	recruitment	log-normal	cv	0.4
	survey catchability	log-normal	cv	0.4
	F reference point	log-normal	cv	0.4

[1] ln(M) = 055 - 1.61 ln L + 1.44 ln Linf + ln K (Gislason H, Daan N, Rice JC, Pope JG. Size, growth, temperature and the natural mortality of marine fish. Fish Fish. 2010;11(2):149–158.)

[2] $R = \frac{aSSB}{b+SSB}$ where $a = \frac{4SSB_0 s}{spr0(5s-1)}$, $b = \frac{SSB_0(1-s)}{5s-1}$, SSB_0 is the SSB of the unfished population and $spr0$ is the spawning stock biomass per recruit of the unfished population (Mace PM, Doonan IJ. A generalised bioeconomic simulation model for fish population dynamics. MAFFish, POB 297, Wellington, NZ.: Fisheries Research Centre, 88/4; 1988.).
