# Supplementary material for: Testing spatial heterogeneity with stock assessment models
Source: PLoS One. 2018 Jan 24;13(1):e0190791. doi: 10.1371/journal.pone.0190791 (PMC5783371; doi:10.1371/journal.pone.0190791)
Supplement: S2 Table — (PDF) [file pone.0190791.s003.pdf]

S2 table Parameters used in the population and fishery simulation loosely based on Northeast Atlantic sardine *Sardina pilchardus*).

subject	process	model	parameter	value
population	growth	von Bertalanffy	L_∞	$23cm$
			k	0.45
			t_0	-1.5
	natural mortality	Gislason[1]		
	length-weight		a	0.0000092
			b	2.98
	reproduction	knife edge	a_{50}	0.7
	stock-recruitment	ricker[2]	virgin biomass (SSB_0)	$1.70e9 - 1.85e9$
			steepness (s)	$\{0.7, 0.75, 0.8, 0.85, 0.9, 0.95\}$
fishery	selectivity	double normal	σ	1
		(dome shape)	σ	3
			L_{50}	3
error	fishing mortality	log-normal	cv	0.4
	recruitment	log-normal	cv	0.4
	survey catchability	log-normal	cv	0.4
	F reference point	log-normal	cv	0.4

[1] ln(M) = 055 - 1.61 ln L + 1.44 ln Linf + ln K (Gislason H, Daan N, Rice JC, Pope JG. Size, growth, temperature and the natural mortality of marine fish. Fish Fish. 2010;11(2):149–158.)

[2] $R = aSSBe^{-bSSB}$ where $a = e^{\frac{b \cdot SSB_0}{spr0}}$, $b = \frac{\log(5 \cdot s)}{0.8 \cdot SSB_0}$, SSB_0 is the SSB of the unfished population and *spr*0 is the spawning stock biomass per recruit of the unfished population.
