# Supplementary material for: Testing spatial heterogeneity with stock assessment models
Source: PLoS One. 2018 Jan 24;13(1):e0190791. doi: 10.1371/journal.pone.0190791 (PMC5783371; doi:10.1371/journal.pone.0190791)
Supplement: S1 File — (PDF) [file pone.0190791.s004.pdf]

Testing spatial heterogeneity with stock assessment models
Appendix I - North Sea cod model sensitivity analysis.

May 11, 2017

Contents

1 Introduction **2**

2 Read data **3**

2.1 Sub-populations 3

3 Fitting - "best sub-models" **5**

3.1 NS cod meta population 5

3.2 Northwest sub-population 7

3.3 South sub-population 10

3.4 Skagerrak + viking sub-population 13

4 Fitting - separable **17**

4.1 NS cod meta population 17

4.2 Northwest sub-population 19

4.3 South sub-population 22

4.4 Skagerrak + viking sub-population 25

5 Comparing tensor with separable fishing mortalities **29**

1 Introduction

This document shows the results from assessments made to different spatial aggregations of the North Sea cod stock data:

- all data combined as in the current stock assessment,
- data split according to a four sub-stock scenario and
- data split according to a three sub-stock scenario.

For each stock definition a comparison was also made between two different assessment approaches; a seperable model for fishing mortality and a time/age variant model (tensor).

The initial putative sub-populations were taken from a synthesis of results from genetic, tagging, modelling of early life stages and knowledge of spawning areas presented to the ICES benchmark assessment of North Sea cod (ICES, 2015a). The areas identified were as shown in Figure 1 of the main text:

- **"skagerrak" & viking**: ICES sub-division IIIa and the North East part of ICES sub-division IVa
- **"northwest"**: The remainder of ICES sub-division IVa (including waters around the Orkney and Shetland islands) and that part of ICES sub-division IVb north of a line running roughly north east from the east English coast at 55N to 57N 4E.
- **"south"**: An area covering ICES sub-divisions VIId, and IVc and a part of ICES sub-division IVb defined by and then east to the Danish coast.

Input data for area-based analyses of stock dynamics and fishing pressure were compiled by sub-areas for the years 2003-2013. Landings in weight by sub-areas were obtained from the EU STECF Fisheries-dependent information database, which includes landing information for EU countries by rectangle. For the UK and Denmark, the STECF estimates were supplemented by landings information from national sources. Norwegian landings, taken from ICES (2015b), were all allocated to the Viking sub-area. For the Skagerrak area, it was possible to supply landings back to 1987, available from national sources and ICES (2015b). The age distribution of landings by sub-areas was available from England & Wales and Denmark. If no area specific information was available the age distribution within the areas was taken to be equal to the overall North Sea wide age structure of landings and mean weights-at-age obtained from ICES (2015b). This was the case for the northwest area. For the skagerrak and viking areas Danish age compositions and mean weights at age were allocated to the total international landings; Denmark takes approzimately 70% and 23% of the landings for these areas. In the south area, age composition data were available from Denmark and England & Wales, that together take an average of 44% of the total landings in this area. An average age composition and mean weights-at-age from the two countries was allocated to the remaining landings to compute numbers-at-age.

For the Skagerrak area, discard numbers-at-age from monitoring programs were available from Denmark for the years 2000-2013. An average discard percentage in relation to landings for 2000-2005, was applied to derive discard estimates for 1987-1999. No discards assumptions were made for other countries. Only Danish discards were added to the international landings to derive catch-at-age. For the viking area, discard numbers-at-age from monitoring programs were available from Denmark for the years 2003-2013. The ratio between Danish discard and Danish landing numbers-at-age by year were applied to the total international landings to derive estimates of total discards in the area. For the northwest area discards-at-age were not available. The ratio between total North Sea wide landing and discard numbers-at-age from ICES (2015b) by year were applied to landings-at-age in the Northwestern area to derive discards. For the south area, discards-at-age estimates from monitoring programs were available for England & Wales and for Denmark for 2003-2013. The ratio between combined landings and discards-at-age from these countries by year was used to derive discard estimates for the rest of the countries fishing cod in the area.

Survey cpue data was obtained from the International Bottom Trawl Survey (IBTS) Q1 and Q3 data available from the DATRAS website. A Delta-GAM model approach for fitting numbers-at-age from DATRAS haul-by-haul exchange data was applied (Berg *et.al*, 2013). The indices are obtained by adding filtered model predictions over aspatial grid. The model is able to account for changes in gear, spatial

coverage, and ship as well as steep depth gradients and country effects. The methodology is as used in ICES (2015b) but applied to each area seperately.

Mean weights-at-age in the stock for each area were estimated from the IBTS Q1 data. Natural mortality and maturity-at-age were kept as used by ICES (2015b).

Following the ideas of Cadrin and Secor (2009) it was considered that additivity of population abundances, such that $N_i = \sum_{j=1}^{m} N_{ij}$, where N is population abundance, i indexes ages and j indexes sub-populations could be used as a test on whether the assumption of largely closed sub-populations could be applied to these areas.

Using the tensor modelling approach the sum of the SSBs from assessments on the four sub-populations and the SSB obtained by fitting to data over the whole area were comparable (Fig. 19) but using the seperable model the sum of SSBs from the four sub-populations were approximately three times the SSB values obtained for the single whole area stock assessment. This lack of consistency between the four sub-population results and whole stock result and the lack of consistency between the separable modelling approach and tensor modelling approach implied the four (closed) sub-population assumption was flawed.

While genetic analysis indicates a weak but temporally stable genetic difference between north east North Sea and Skagerrak cod, tagging studies and the observed distribution of juveniles suggest that many juvenile cod that grow up in coastal Skagerrak waters migrate back towards the North Sea when reaching maturity. This is believed to be evidence of natal fidelity acting upon fish that were advected as eggs from North Sea spawning grounds (Andre *et.al*, 2016).

It was therefore decided that when it came to fitting stock assessment models it was necessary to merge the Skagerrak and Viking areas. When the resulting three areas were fitted the sum of SSBs form the individual areas were comparable to the SSB obtained fitting to data over the whole area from both the tensor and separable models. The evidence for sub-populations from the literature does not contain any evidence for strong migrations of any age classes between the remaining three putative areas. This, combined with the consistent SSB results, lead the study to conclude the three sub-population scenario was the best to adopt given current scientific evidence.

2 Read data

```
load("cod347_FLStockObject.RData")
load("cod347_FLIndexObject_both.RData")
cod1 <- iter(cod, 1)
```

2.1 Sub-populations

```
# ~~~~~~~~~~~~~~~~~~~~~~~~~~~~~~~~~~~~~~~~~~~~~~~~~~~~~~~~~~~~~~~~~~~~~~~~
# northwest
# ~~~~~~~~~~~~~~~~~~~~~~~~~~~~~~~~~~~~~~~~~~~~~~~~~~~~~~~~~~~~~~~~~~~~~~~~

codnw.stk <- readFLStock("NW/files.ind", name = "NSCOD-NW", no.discards = TRUE,
    harvest.units = "f")
catch(codnw.stk) <- computeCatch(codnw.stk)
landings(codnw.stk) <- computeLandings(codnw.stk)
discards(codnw.stk) <- computeDiscards(codnw.stk)
harvest.spwn(codnw.stk) <- 0
m.spwn(codnw.stk) <- 0
codnw.stk <- setPlusGroup(codnw.stk, 7)

## [1] "maxfbar has been changed to accomodate new plusgroup"
```

```
range(codnw.stk)[c("minfbar", "maxfbar")] <- c(2, 4)
codnw.ids <- readFLIndices("NW/survey.dat")
codnw.stk <- window(codnw.stk, start = 2003, end = 2013)
codnw.ids <- window(codnw.ids, start = 2003, end = 2013)
# FLCore bug ...
names(codnw.ids) <- c("NW_Q1", "NW_Q3")
```

```
# ~~~~~~~~~~~~~~~~~~~~~~~~~~~~~~~~~~~~~~~~~~~~~~~~~~~~~~~~~~~~~~~~~~~~
# southern
# ~~~~~~~~~~~~~~~~~~~~~~~~~~~~~~~~~~~~~~~~~~~~~~~~~~~~~~~~~~~~~~~~~~~~

codso.stk <- readFLStock("South/files.ind", name = "NSCOD-SO", no.discards = TRUE,
    harvest.units = "f")
catch(codso.stk) <- computeCatch(codso.stk)
landings(codso.stk) <- computeLandings(codso.stk)
discards(codso.stk) <- computeDiscards(codso.stk)
harvest.spwn(codso.stk) <- 0
m.spwn(codso.stk) <- 0
codso.stk <- setPlusGroup(codso.stk, 7)

## [1] "maxfbar has been changed to accomodate new plusgroup"

range(codso.stk)[c("minfbar", "maxfbar")] <- c(2, 4)
codso.ids <- readFLIndices("South/survey.dat")
codso.stk <- window(codso.stk, start = 2003, end = 2013)
codso.ids <- window(codso.ids, start = 2003, end = 2013)
# FLCore bug ...
names(codso.ids) <- c("South_Q1", "South_Q3")
```

```
# ~~~~~~~~~~~~~~~~~~~~~~~~~~~~~~~~~~~~~~~~~~~~~~~~~~~~~~~~~~~~~~~~~~~~
# skagerrak + viking
# ~~~~~~~~~~~~~~~~~~~~~~~~~~~~~~~~~~~~~~~~~~~~~~~~~~~~~~~~~~~~~~~~~~~~
codskvk.stk <- readFLStock("SKVK/files.ind", name = "NSCOD-SKVK", no.discards = TRUE,
    harvest.units = "f")
catch(codskvk.stk) <- computeCatch(codskvk.stk)
landings(codskvk.stk) <- computeLandings(codskvk.stk)
discards(codskvk.stk) <- computeDiscards(codskvk.stk)
harvest.spwn(codskvk.stk) <- 0
m.spwn(codskvk.stk) <- 0
codskvk.stk <- setPlusGroup(codskvk.stk, 7)

## [1] "maxfbar has been changed to accomodate new plusgroup"

range(codskvk.stk)[c("minfbar", "maxfbar")] <- c(2, 4)
codskvk.ids <- readFLIndices("SKVK/survey.dat")
# FLCore bug ...
names(codskvk.ids) <- c("VikingSk_Q1", "VikingSk_Q3")

codskvk.stk <- window(codskvk.stk, start = 2003, end = 2013)
codskvk.ids <- window(codskvk.ids, start = 2003, end = 2013)
```

3 Fitting - "best sub-models"

```
# ~~~~~~~~~~~~~~~~~~~~~~~~~~~~~~~~~~~~~~~~~~~~~~~~~~~~~~~~~~~~~~~~~~~~
# sub-models
# ~~~~~~~~~~~~~~~~~~~~~~~~~~~~~~~~~~~~~~~~~~~~~~~~~~~~~~~~~~~~~~~~~~~~
fmod <- ~te(replace(age, age > 6, 6), year, k = c(4, 5)) + s(year, k = 3, by = as.numeric(age ==
    1))
qmod <- list(~s(age, k = 5), ~s(age, k = 4) + s(year, k = 3))
mc <- SCAMCMC(mcmc = 125000, mcsave = 250, mcprobe = 0.4, mcrb = 3)
```

3.1 NS cod meta population

```
stk <- window(cod1, 2003, 2013)
ids <- window(cod.tun, 2003, end = 2013)
fit <- sca(stk, ids, fit = "assessment", fmodel = fmod, qmodel = qmod)
res <- residuals(fit, stk, ids)
plot(res)
```

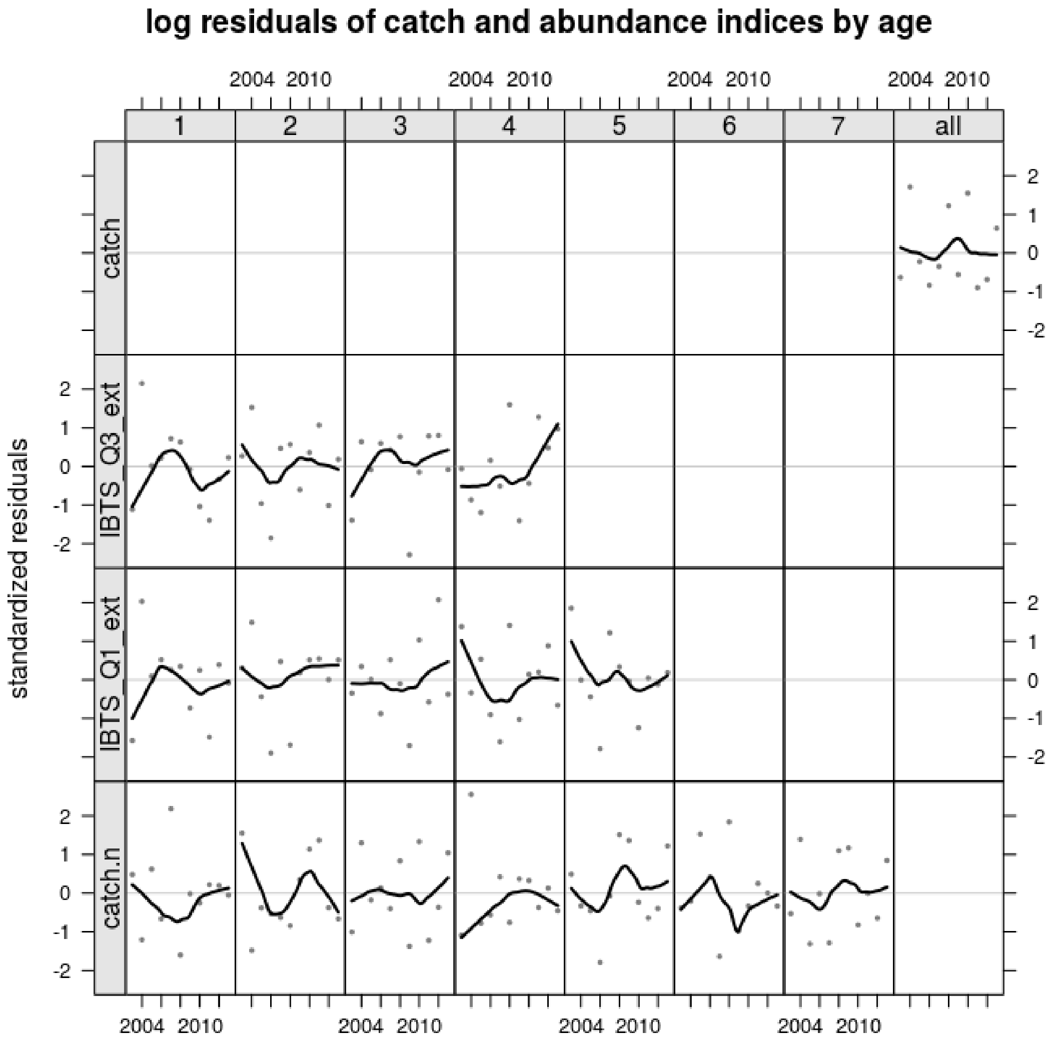

```r
wireframe(data ~ year + age, data = harvest(fit))
```

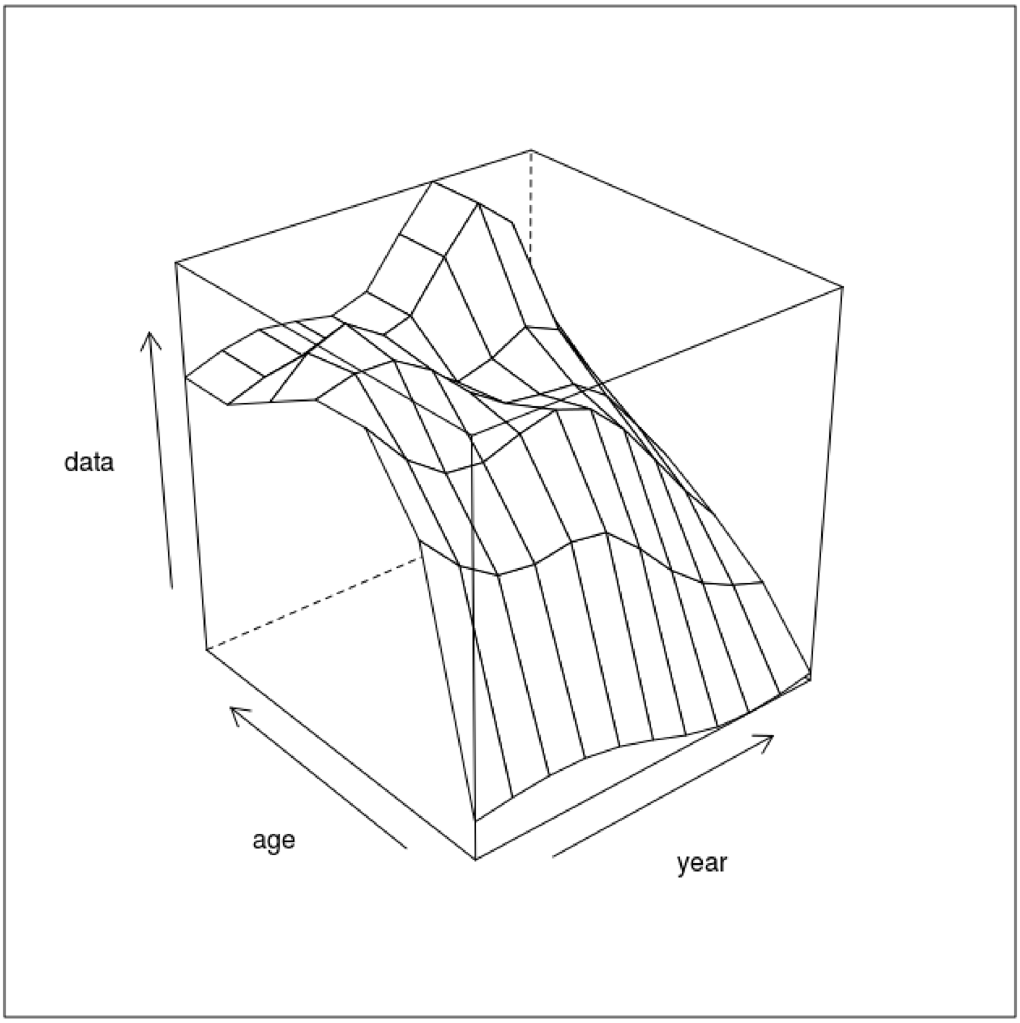


```r
# cod.fit <- fit
cod.fstk <- stk + fit
cod.fstks <- stk + a4aSCA(stk, ids, fit = "MCMC", fmodel = fmod, qmodel = qmod,
    mcmc = mc)
plot(cod.fstks)
```

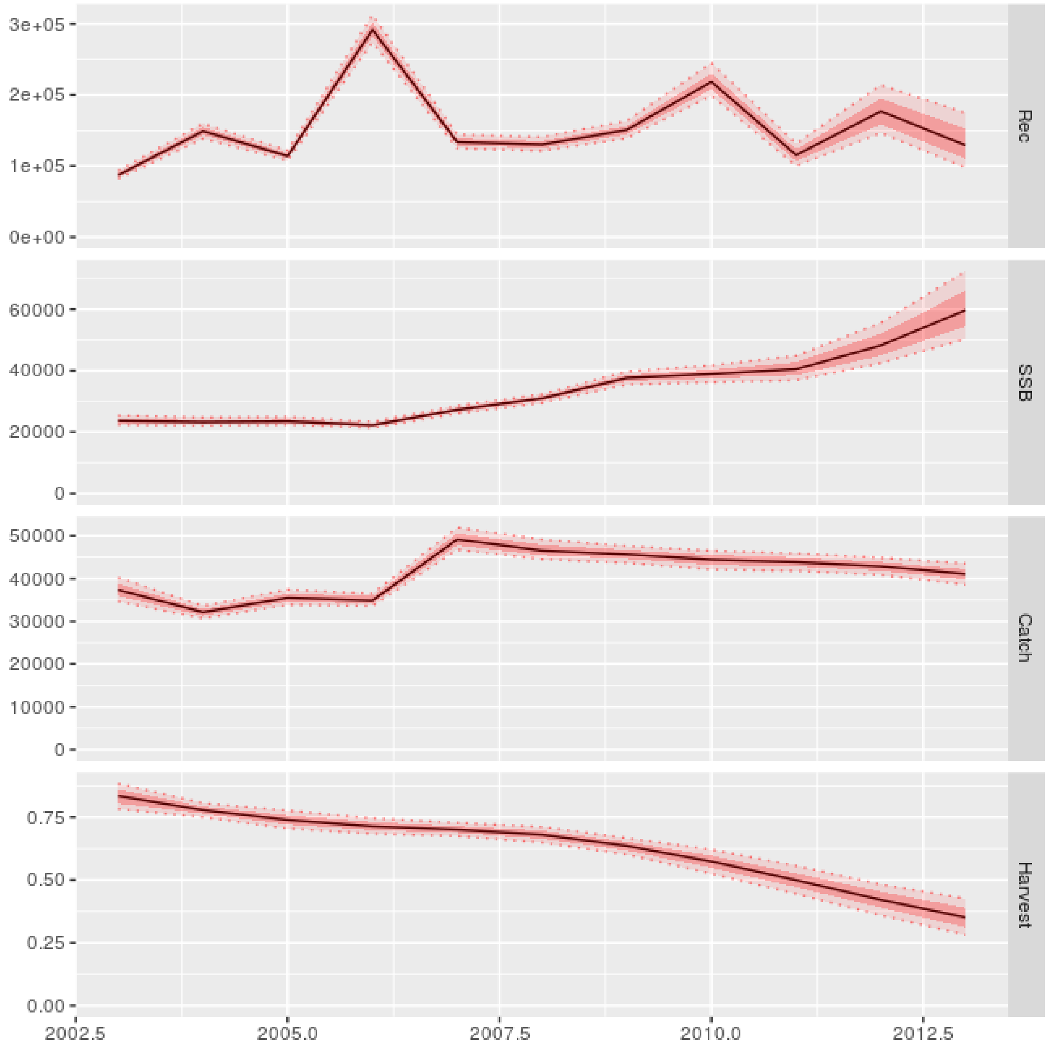

3.2 Northwest sub-population

```
stk <- codnw.stk
ids <- codnw.ids
ids[[1]] <- trim(ids[[1]], age = 1:5)
ids[[2]] <- trim(ids[[2]], age = 1:4)
fit <- a4aSCA(stk, ids, fit = "assessment", fmodel = fmod, qmodel = qmod)
res <- residuals(fit, stk, ids)
plot(res)
```

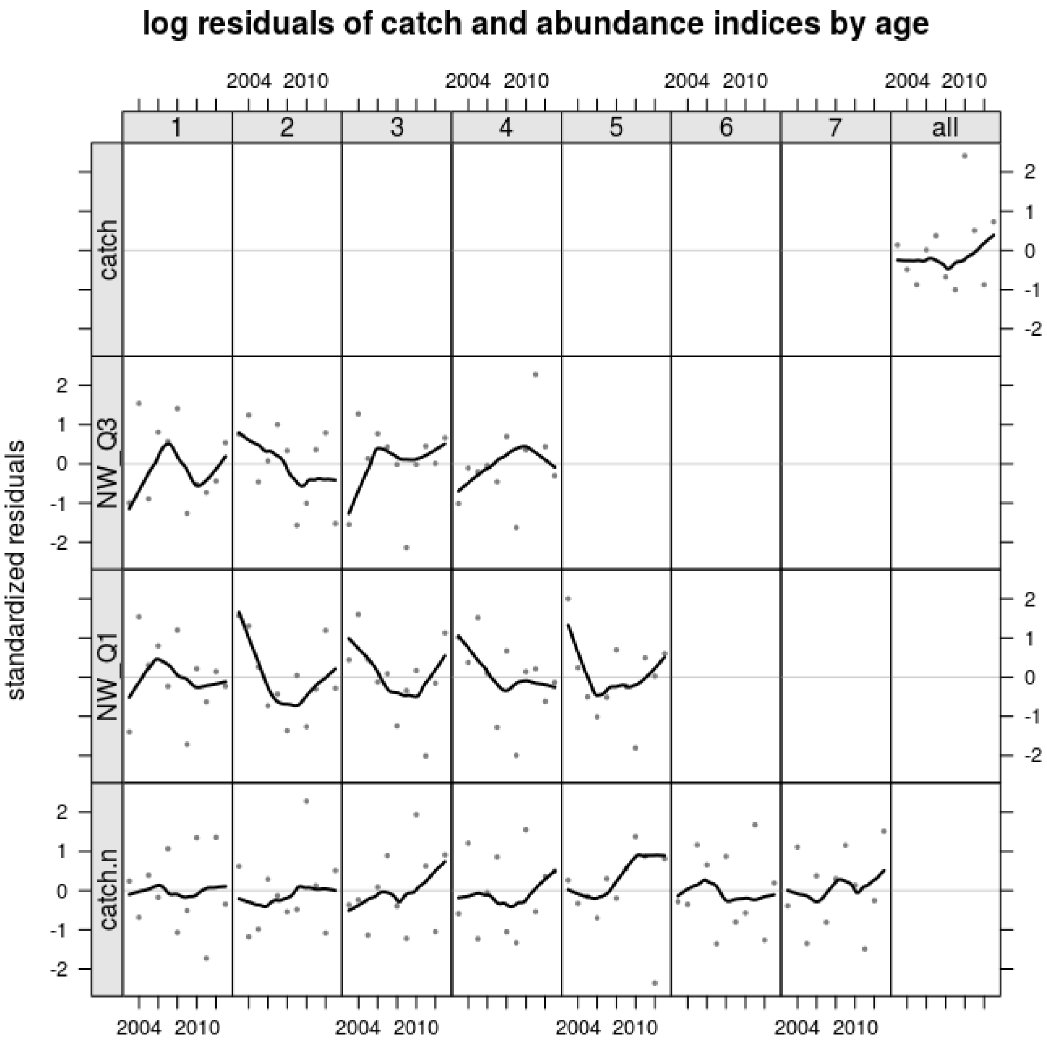


```
wireframe(data ~ year + age, data = harvest(fit))
```

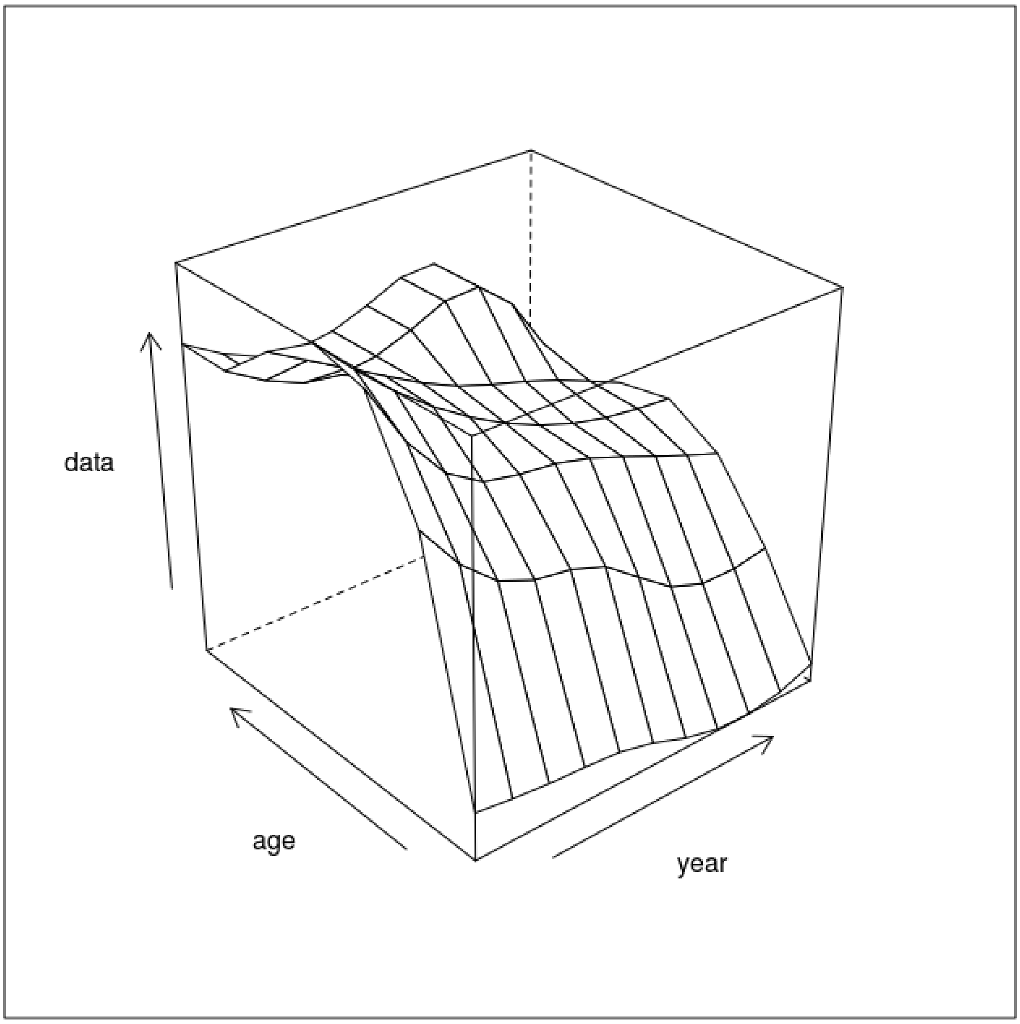


```
# codnw.fit <- fit
codnw.fstk <- stk + fit
codnw.fstks <- stk + a4aSCA(stk, ids, fit = "MCMC", fmodel = fmod, qmodel = qmod,
    mcmc = mc)
plot(codnw.fstks)
```

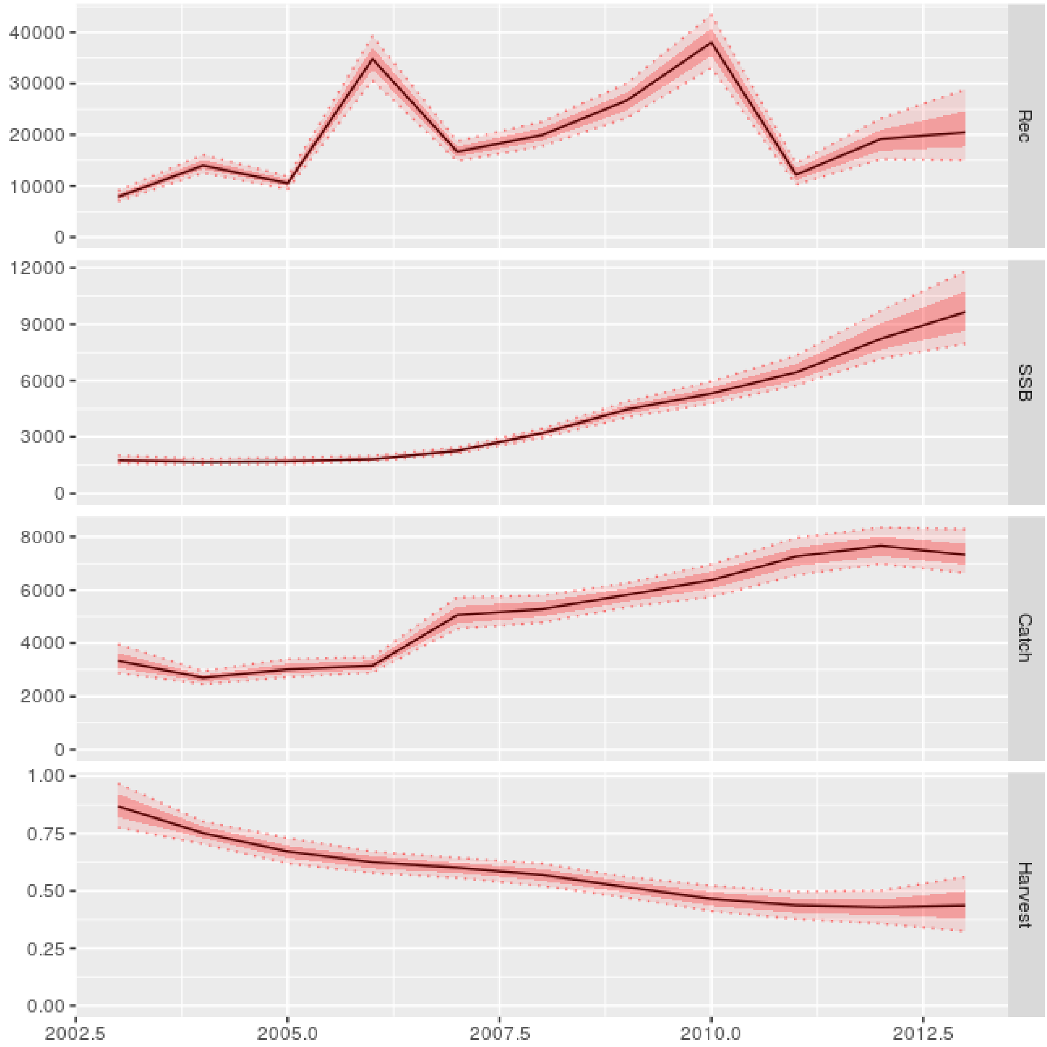

3.3 South sub-population

```
stk <- codso.stk
ids <- codso.ids
ids[[1]] <- trim(ids[[1]], age = 1:5)
ids[[2]] <- trim(ids[[2]], age = 1:4)
fit <- sca(stk, ids, fit = "assessment", fmodel = fmod, qmodel = qmod)
res <- residuals(fit, stk, ids)
plot(res)
```

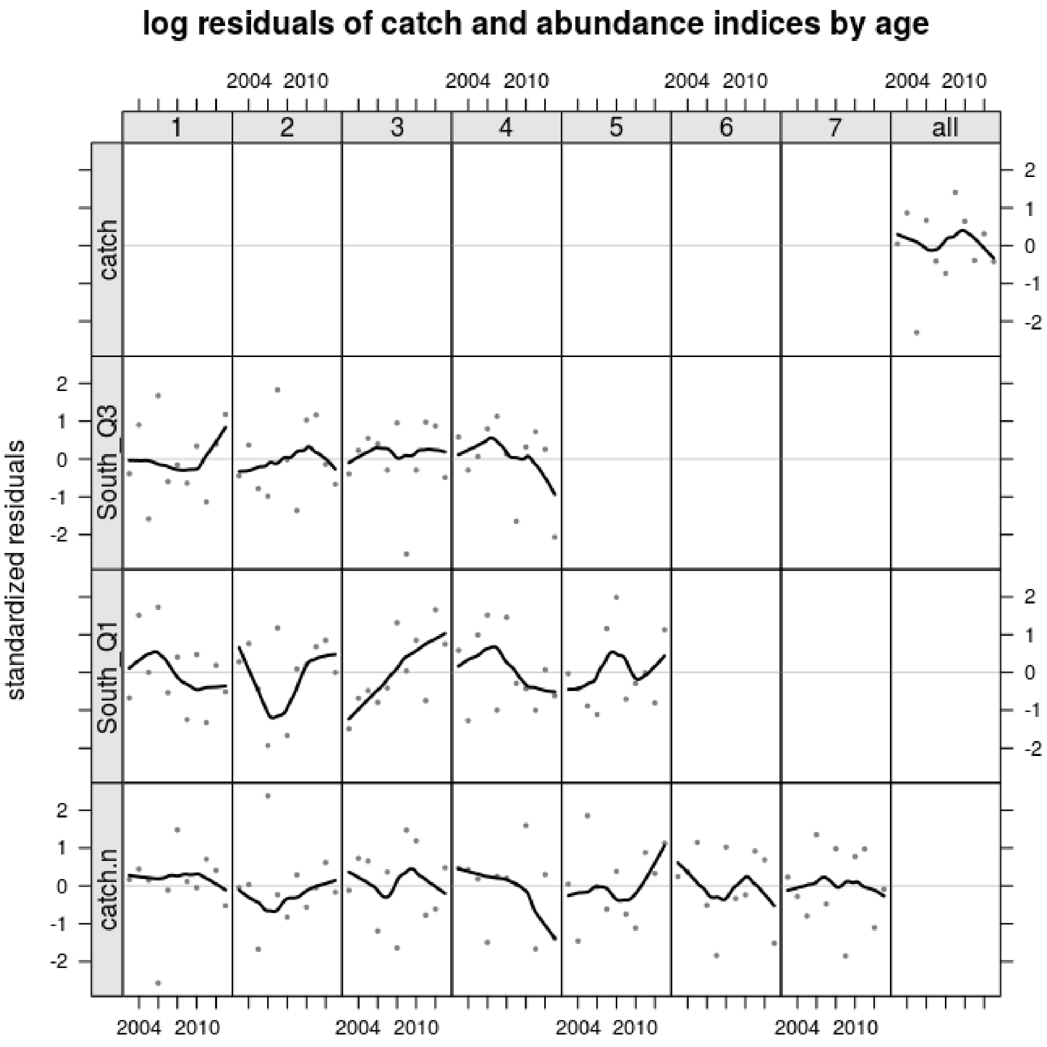


```r
wireframe(data ~ year + age, data = harvest(fit))
```

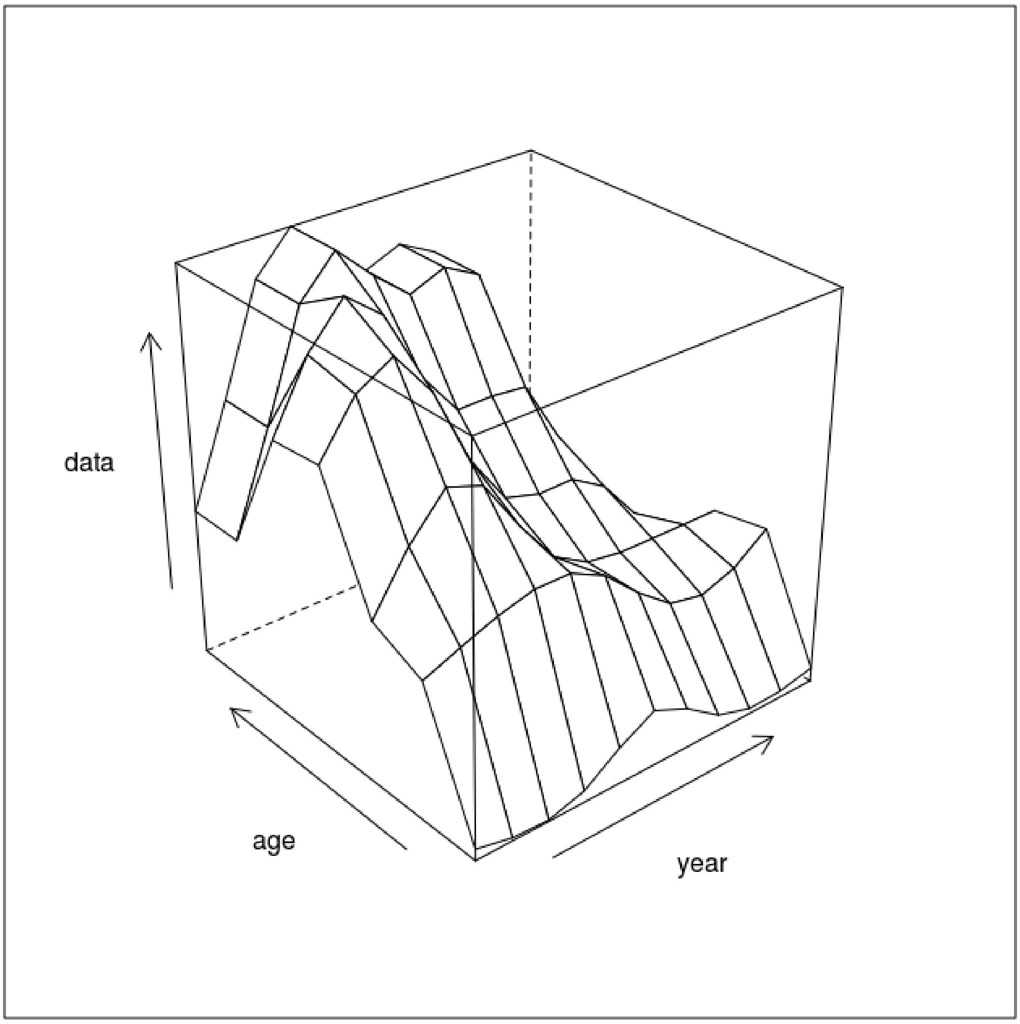


```
# codso.fit <- fit
codso.fstk <- stk + fit
codso.fstks <- stk + a4aSCA(stk, ids, fit = "MCMC", fmodel = fmod, qmodel = qmod,
    mcmc = mc)
plot(codso.fstks)
```

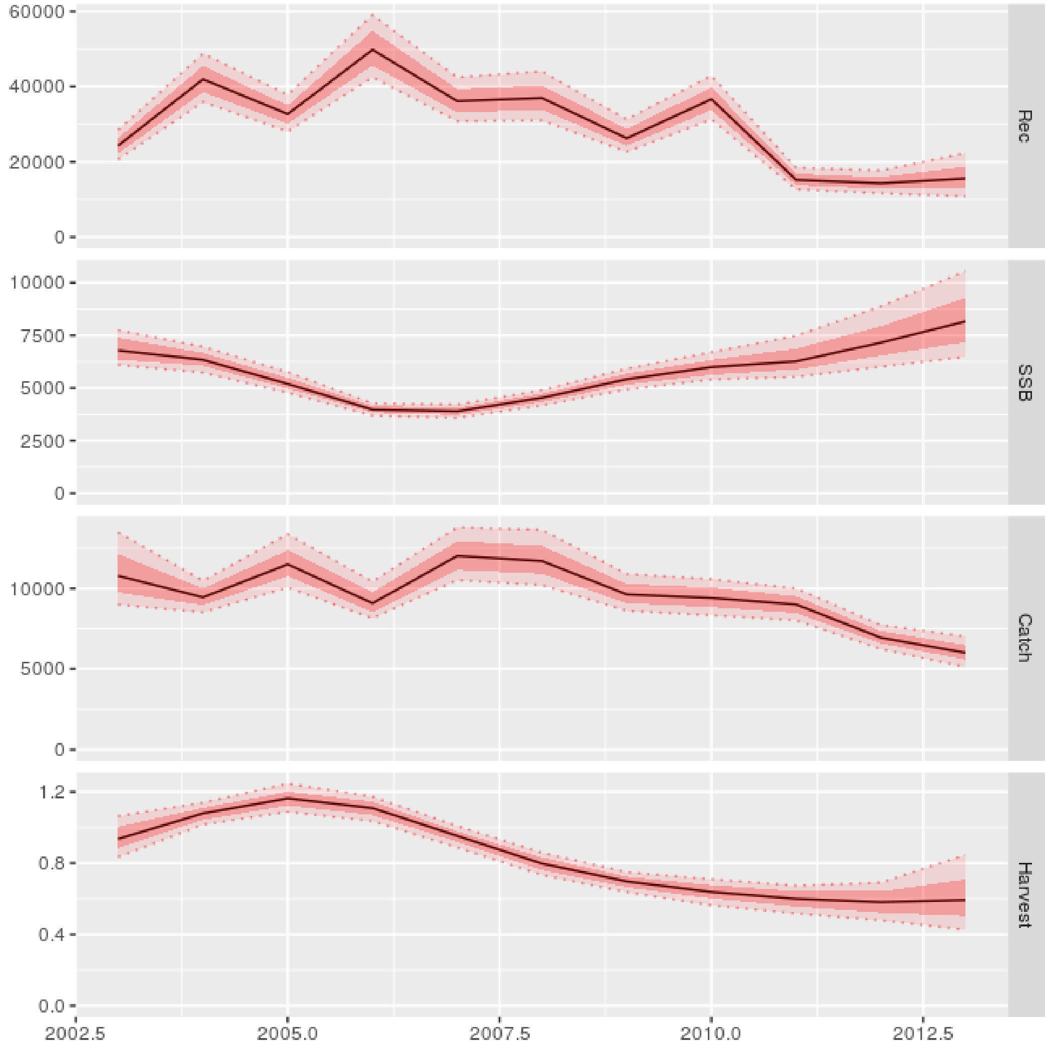

3.4 Skagerrak + viking sub-population

```
stk <- codskvk.stk
ids <- codskvk.ids
ids[[1]] <- trim(ids[[1]], age = 1:5)
ids[[2]] <- trim(ids[[2]], age = 1:4)
fit <- sca(stk, ids, fit = "assessment", fmodel = fmod, qmodel = qmod)
res <- residuals(fit, stk, ids)
plot(res)
```

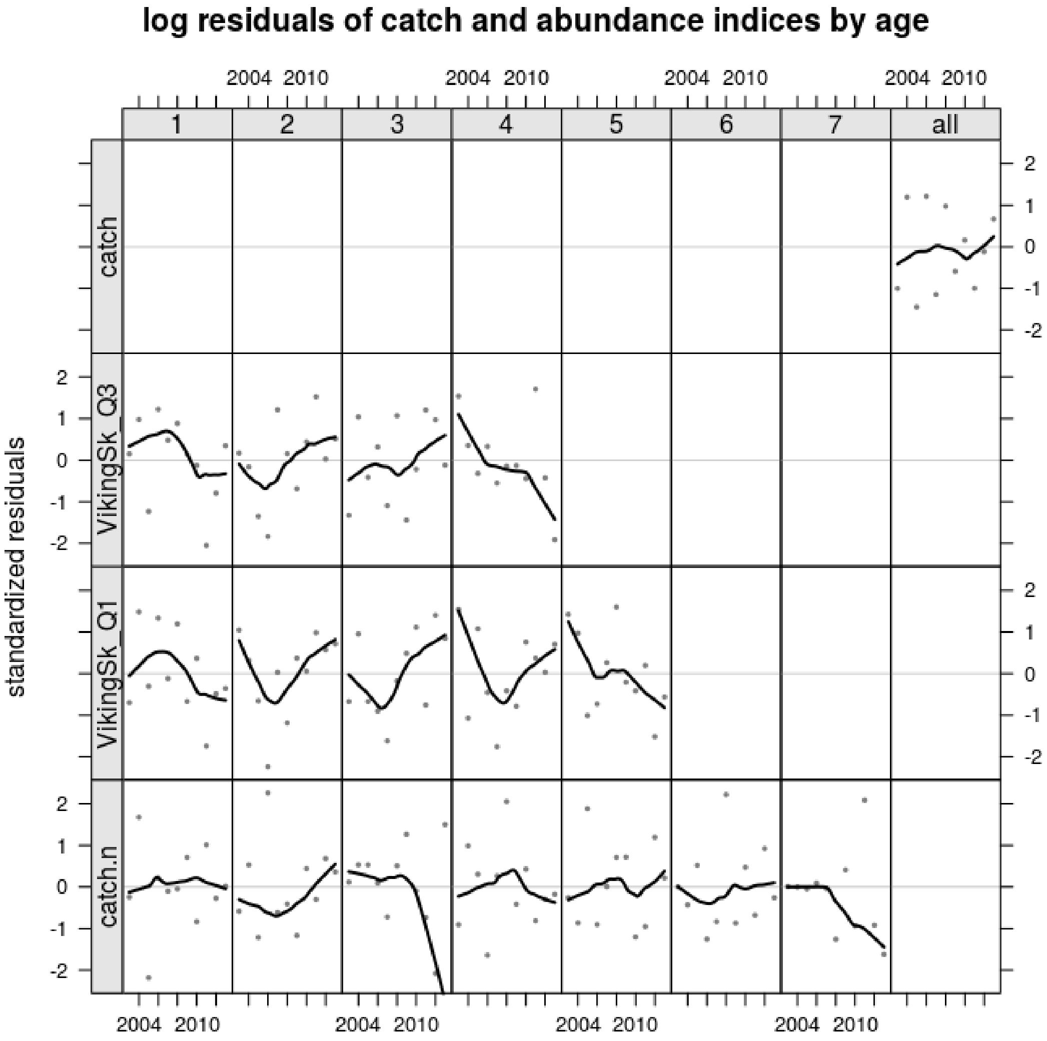


```r
wireframe(data ~ year + age, data = harvest(fit))
```

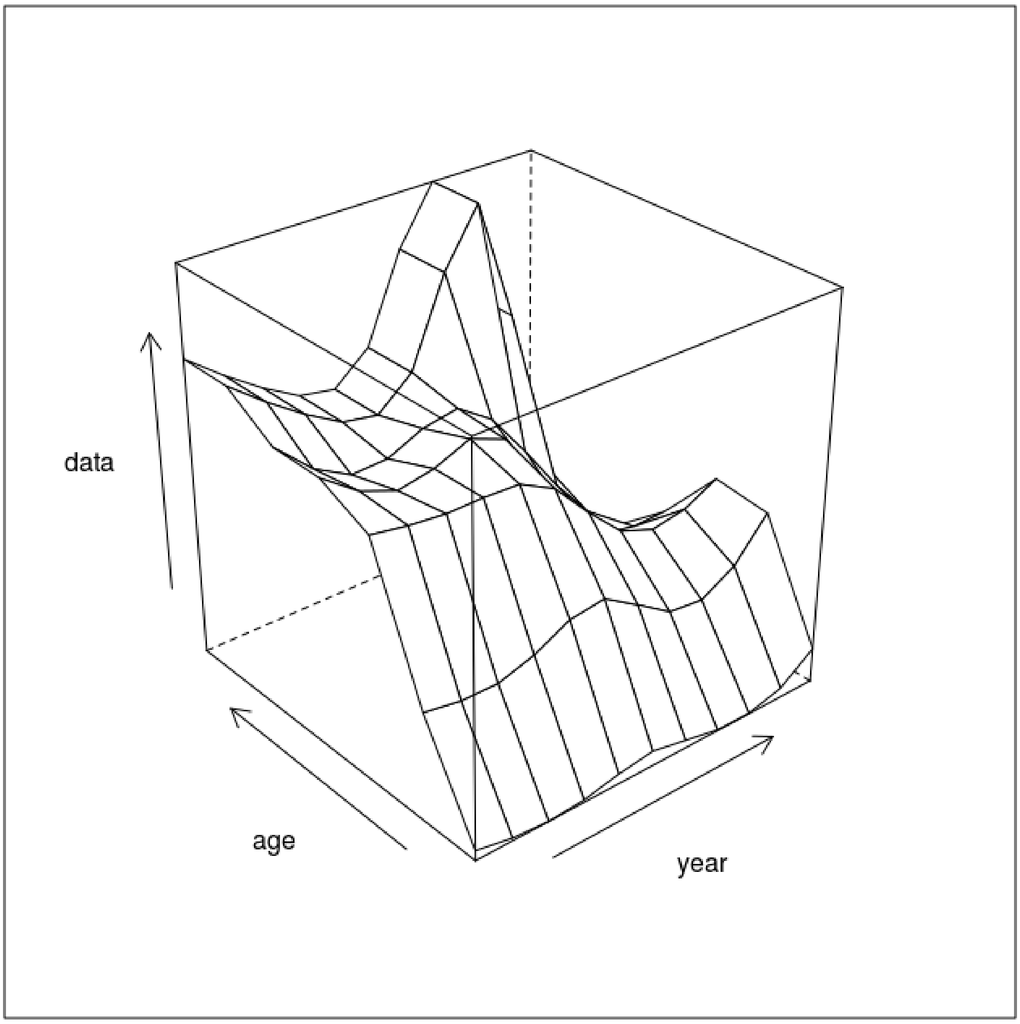


```
# codskvk.fit <- fit
codskvk.fstk <- stk + fit
codskvk.fstks <- stk + a4aSCA(stk, ids, fit = "MCMC", fmodel = fmod, qmodel = qmod,
    mcmc = mc)
plot(codskvk.fstks)
```

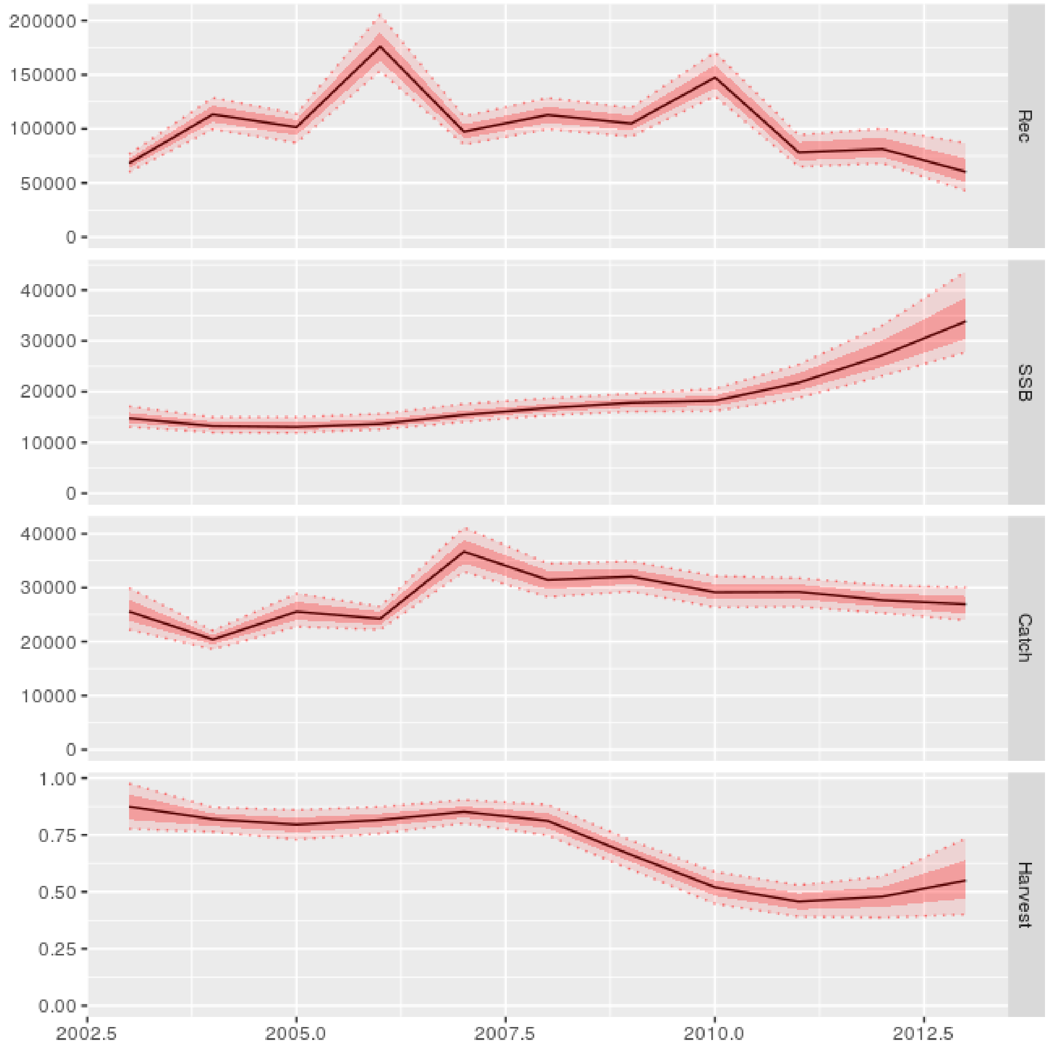


```
cod3ssb <- ssb(codnw.fstk) + ssb(codso.fstk) + ssb(codskvk.fstk)
ssb.flqs <- FLQuants(`meta-population` = ssb(cod.fstk), `sub-populations:tensor` = cod3ssb)

cod3n <- stock.n(codnw.fstk) + stock.n(codso.fstk) + stock.n(codskvk.fstk)
n.flqs <- FLQuants(`meta-population` = stock.n(cod.fstk), `sub-populations:tensor` = cod3n)
```

4 Fitting - separable

```
# ~~~~~~~~~~~~~~~~~~~~~~~~~~~~~~~~~~~~~~~~~~~~~~~~~~~~~~~~~~~~~~~~~~~~~~
# sub-models
# ~~~~~~~~~~~~~~~~~~~~~~~~~~~~~~~~~~~~~~~~~~~~~~~~~~~~~~~~~~~~~~~~~~~~~~
fmod <- ~s(replace(age, age > 6, 6), k = 5) + s(year, k = 10)
qmod <- list(~s(age, k = 5), ~s(age, k = 4) + s(year, k = 3))
```

4.1 NS cod meta population

```
stk <- window(cod1, 2003, 2013)
ids <- window(cod.tun, 2003, end = 2013)
fit <- sca(stk, ids, fit = "assessment", fmodel = fmod, qmodel = qmod)
res <- residuals(fit, stk, ids)
plot(res)
```

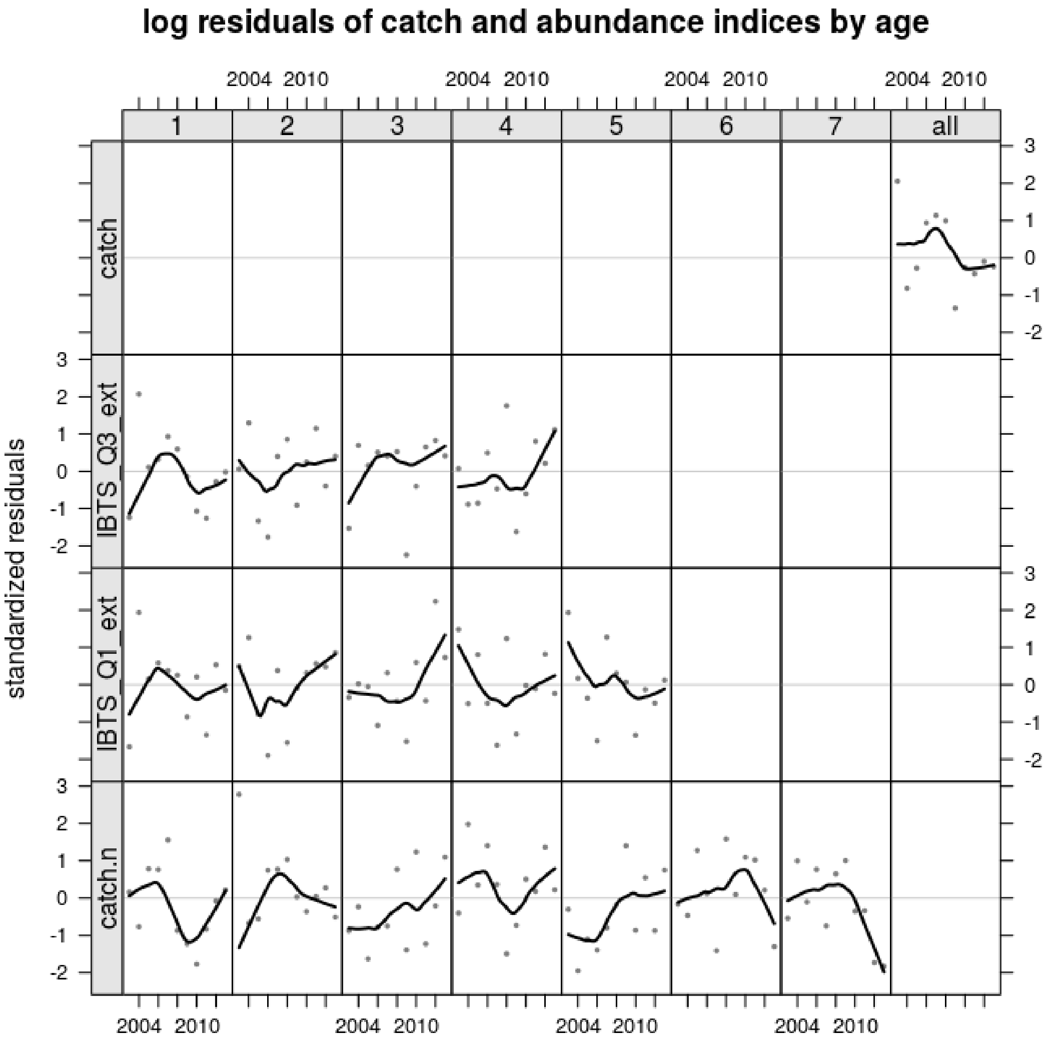


```
wireframe(data ~ year + age, data = harvest(fit))
```

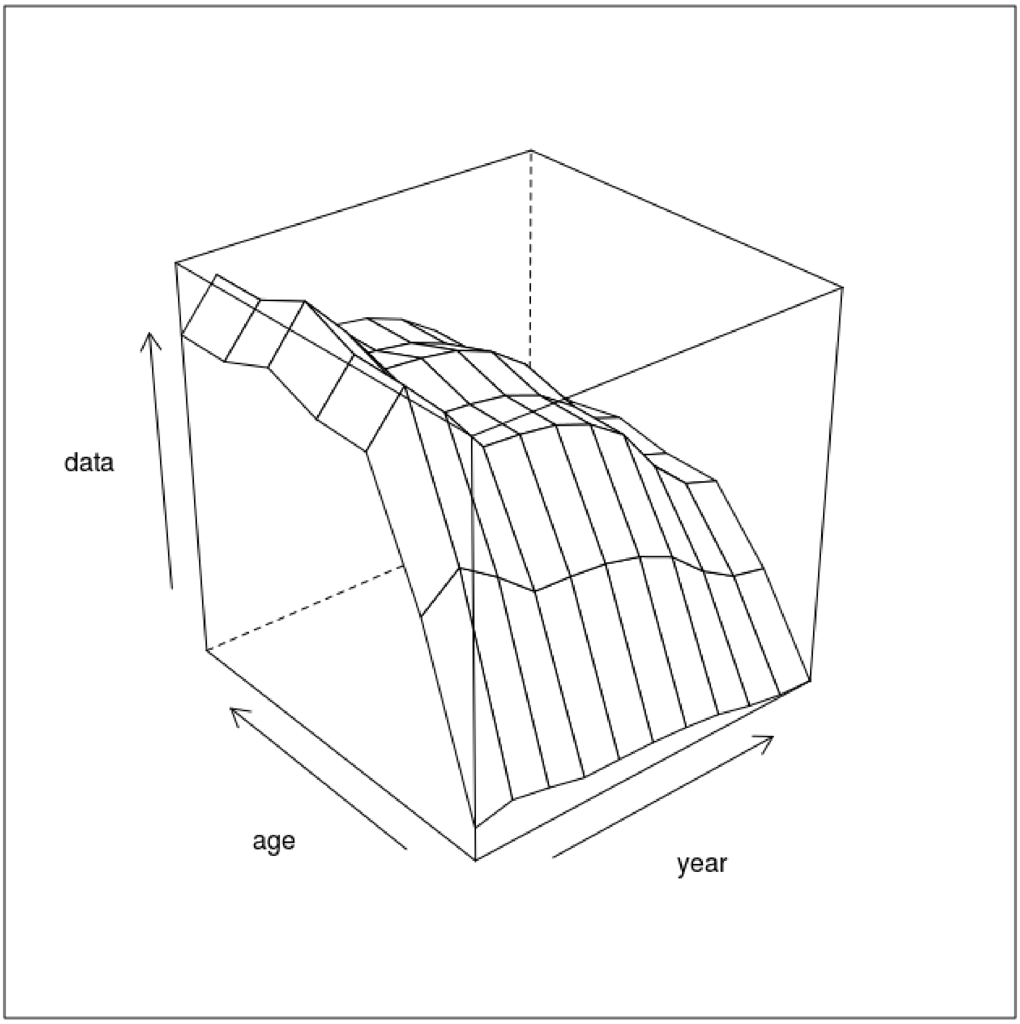


```
# cod.fit <- fit
cod.fstk <- stk + fit
cod.fstks <- stk + a4aSCA(stk, ids, fit = "MCMC", fmodel = fmod, qmodel = qmod,
    mcmc = mc)
plot(cod.fstks)
```

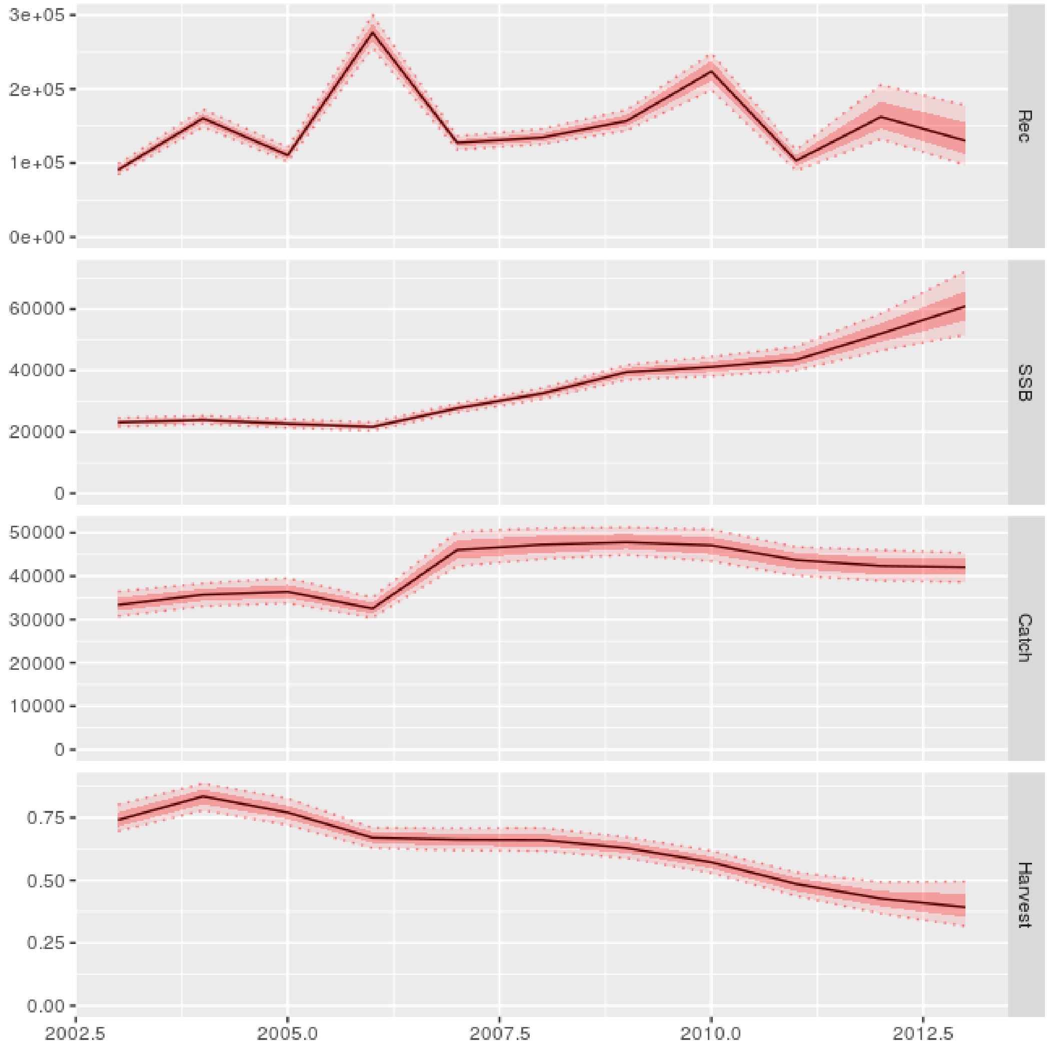

4.2 Northwest sub-population

```
stk <- codnw.stk
ids <- codnw.ids
ids[[1]] <- trim(ids[[1]], age = 1:5)
ids[[2]] <- trim(ids[[2]], age = 1:4)
fit <- a4aSCA(stk, ids, fit = "assessment", fmodel = fmod, qmodel = qmod)
res <- residuals(fit, stk, ids)
plot(res)
```

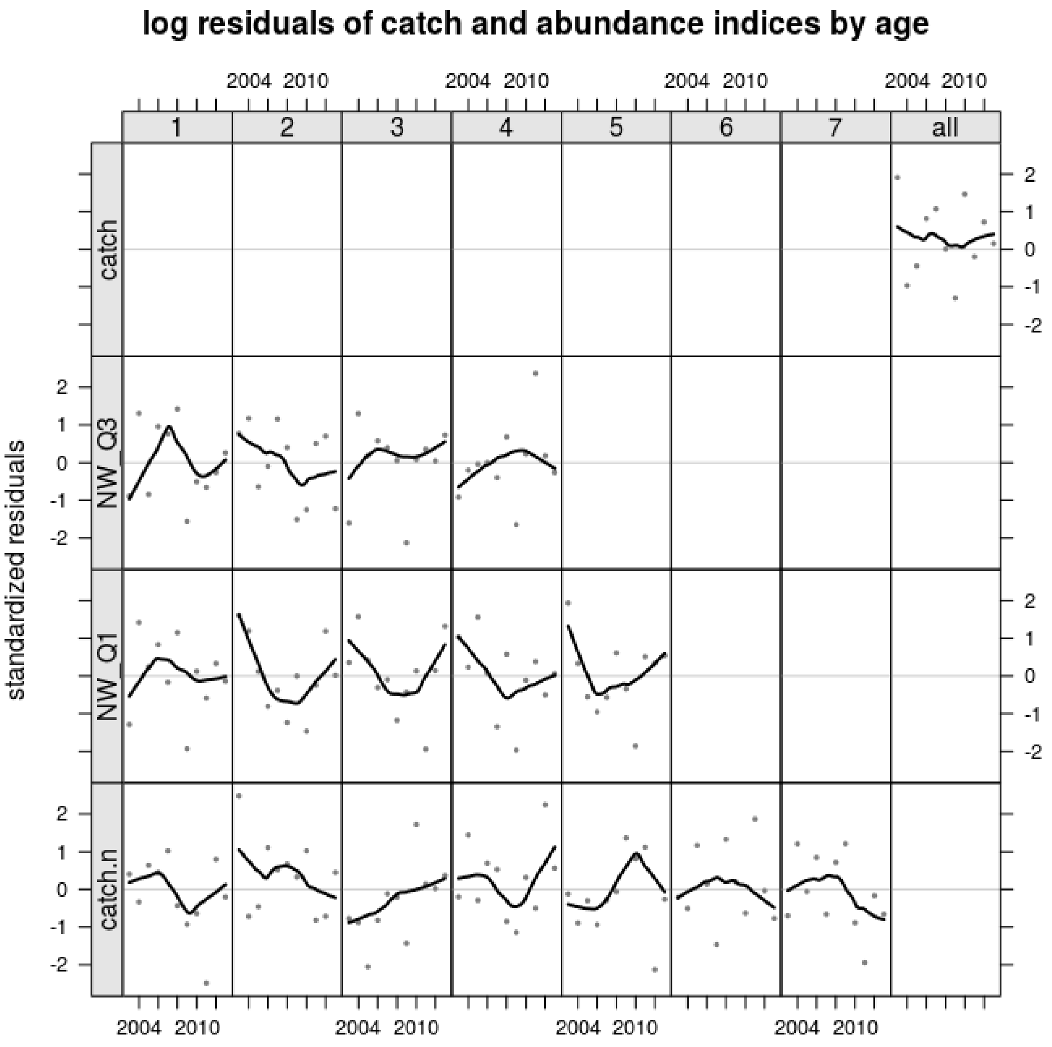


```r
wireframe(data ~ year + age, data = harvest(fit))
```

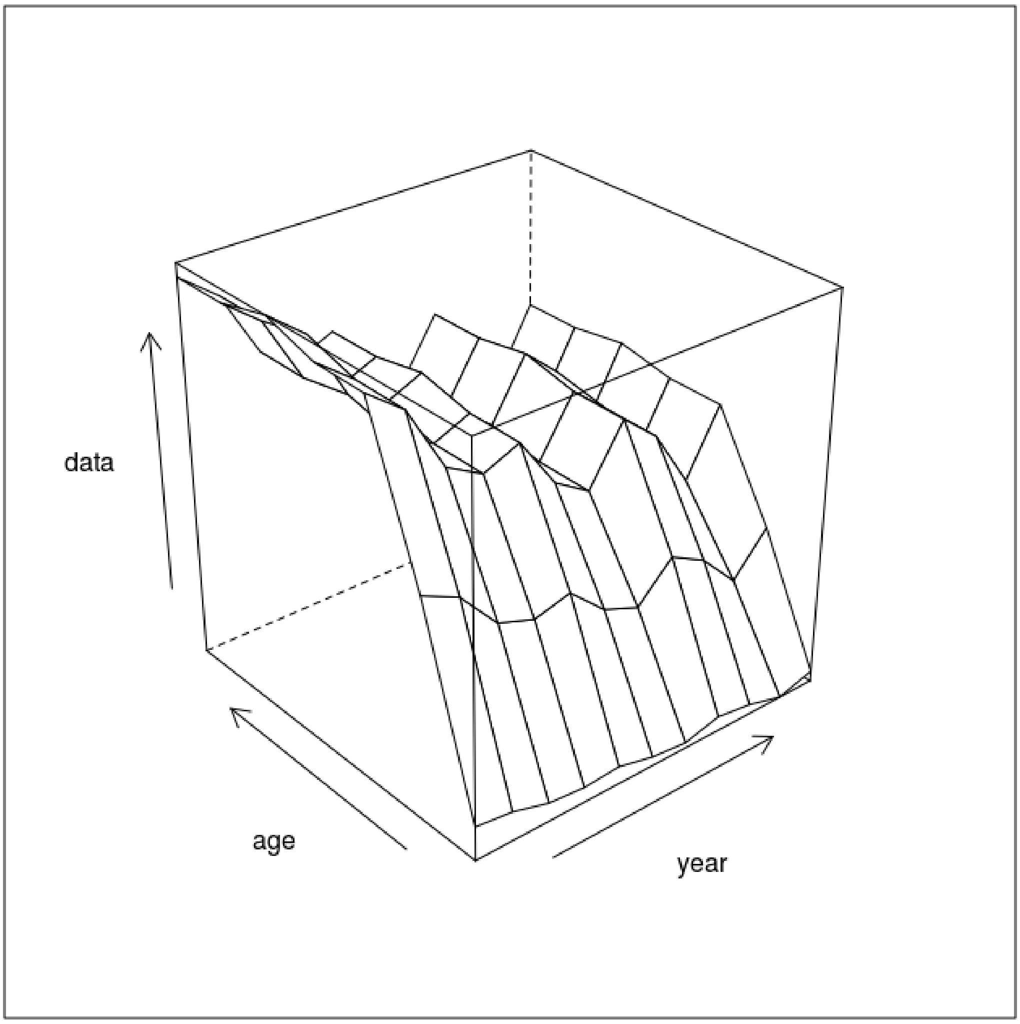


```
# codnw.fit <- fit
codnw.fstk <- stk + fit
codnw.fstks <- stk + a4aSCA(stk, ids, fit = "MCMC", fmodel = fmod, qmodel = qmod,
    mcmc = mc)
plot(codnw.fstks)
```

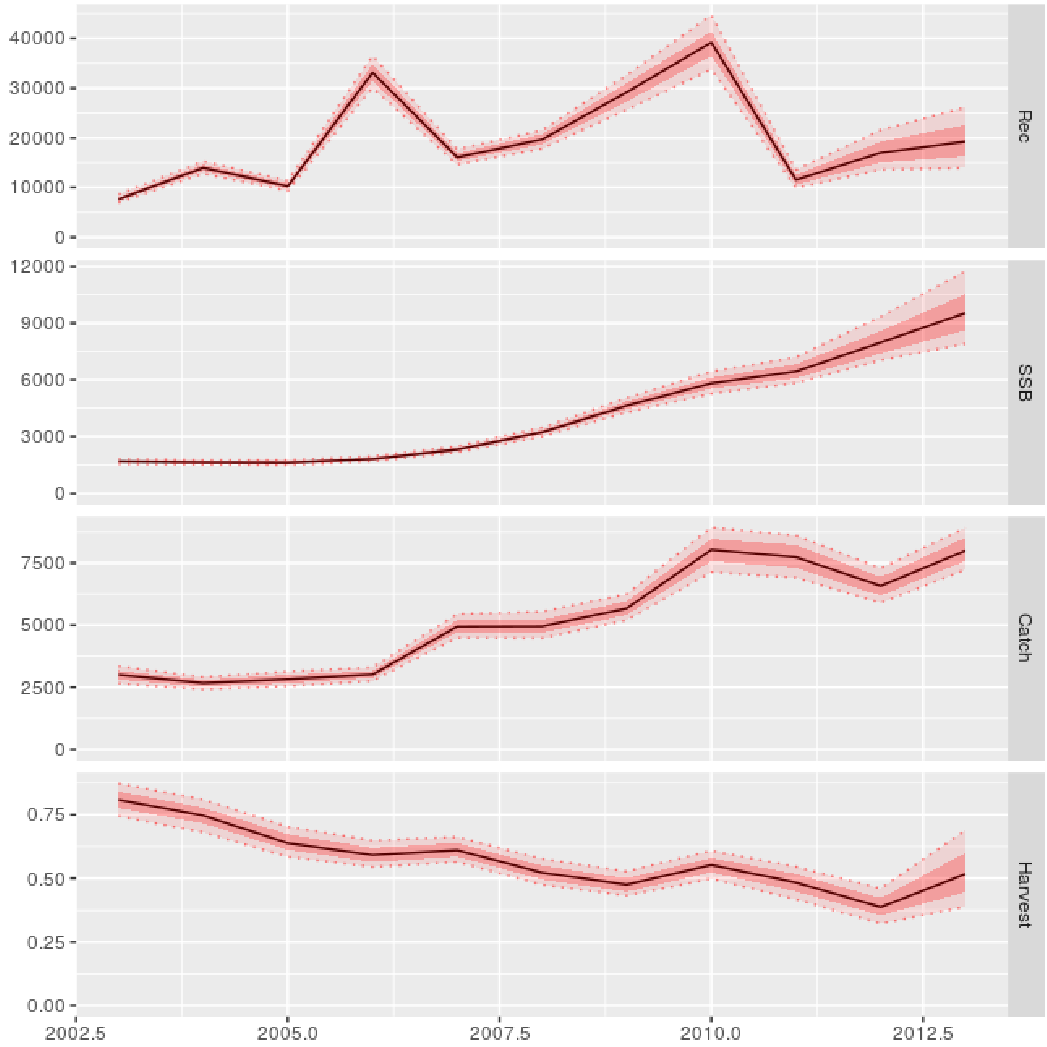

4.3 South sub-population

```
stk <- codso.stk
ids <- codso.ids
ids[[1]] <- trim(ids[[1]], age = 1:5)
ids[[2]] <- trim(ids[[2]], age = 1:4)
fit <- sca(stk, ids, fit = "assessment", fmodel = fmod, qmodel = qmod)
res <- residuals(fit, stk, ids)
plot(res)
```

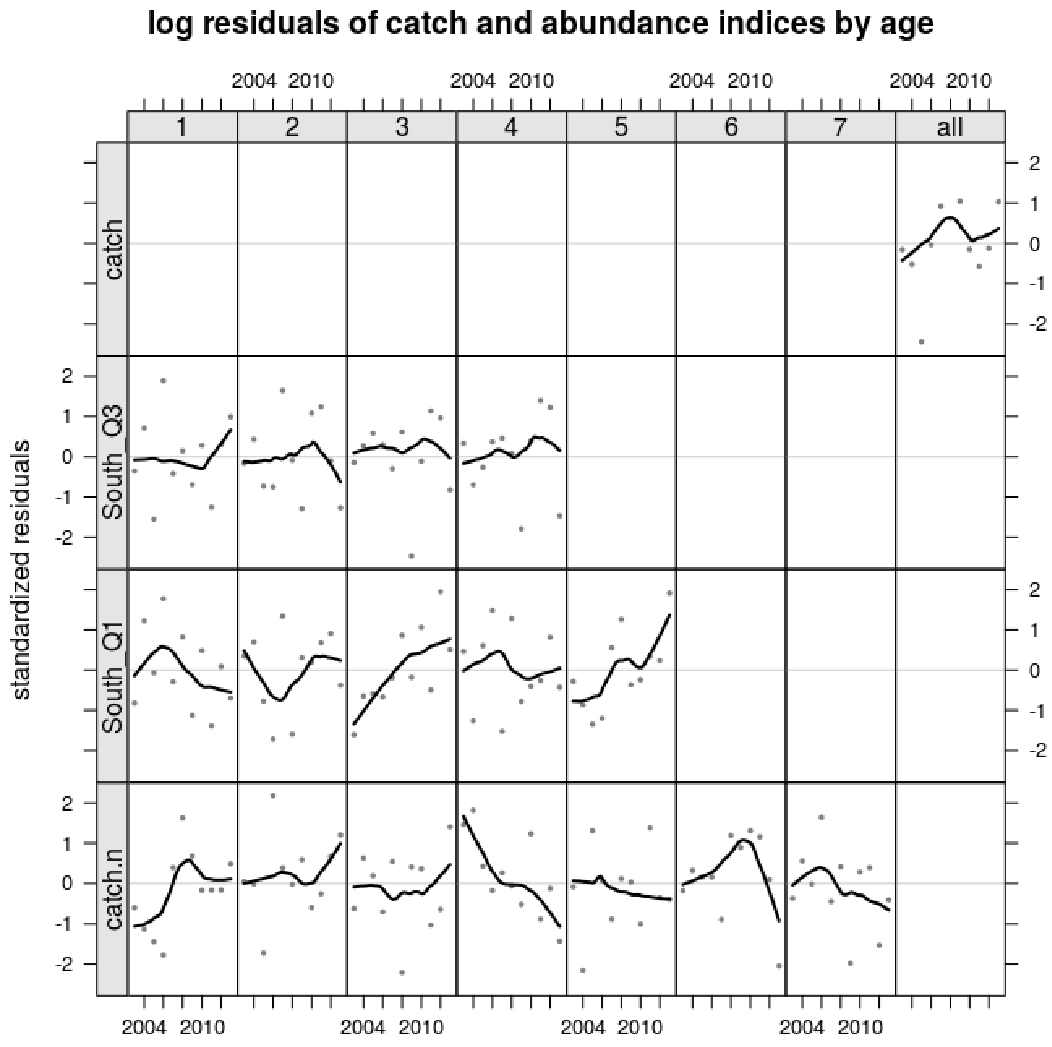


```
wireframe(data ~ year + age, data = harvest(fit))
```

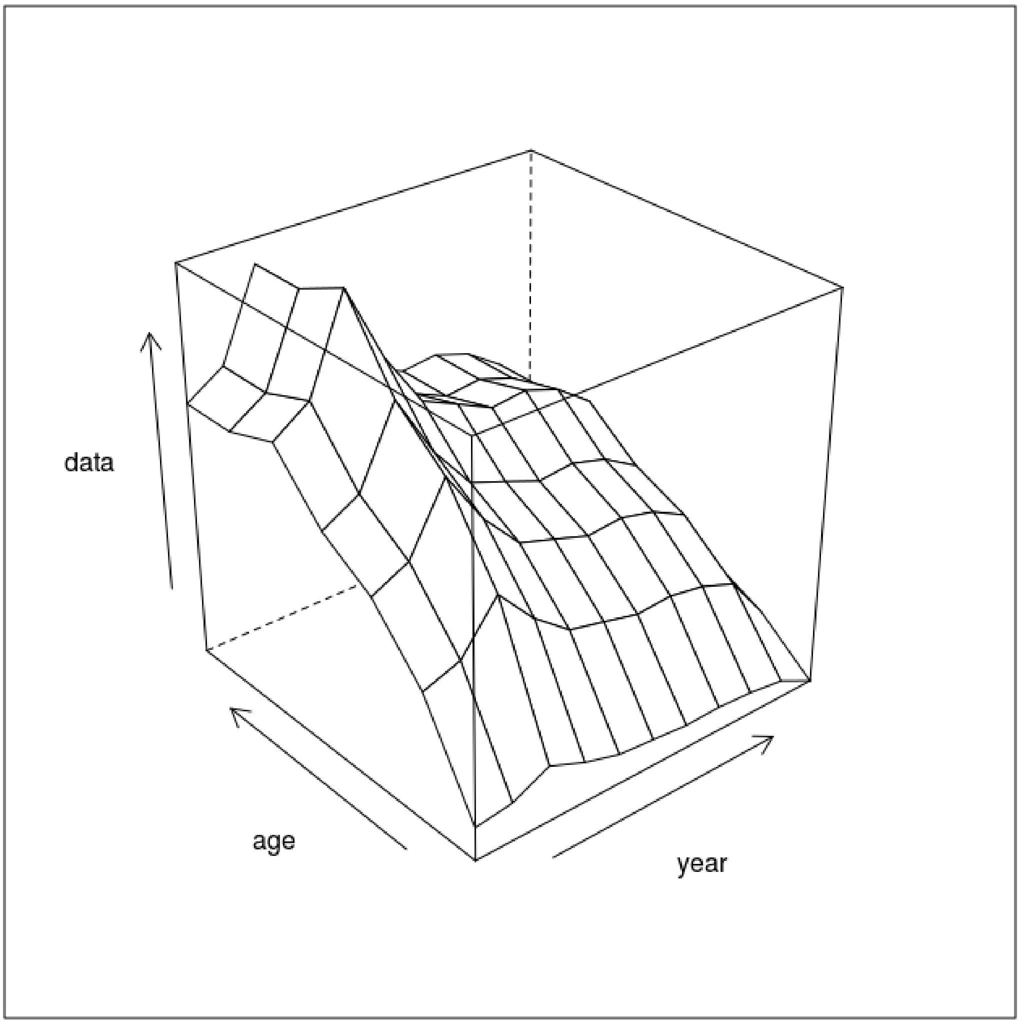


```
# codso.fit <- fit
codso.fstk <- stk + fit
codso.fstks <- stk + a4aSCA(stk, ids, fit = "MCMC", fmodel = fmod, qmodel = qmod,
    mcmc = mc)
plot(codso.fstks)
```

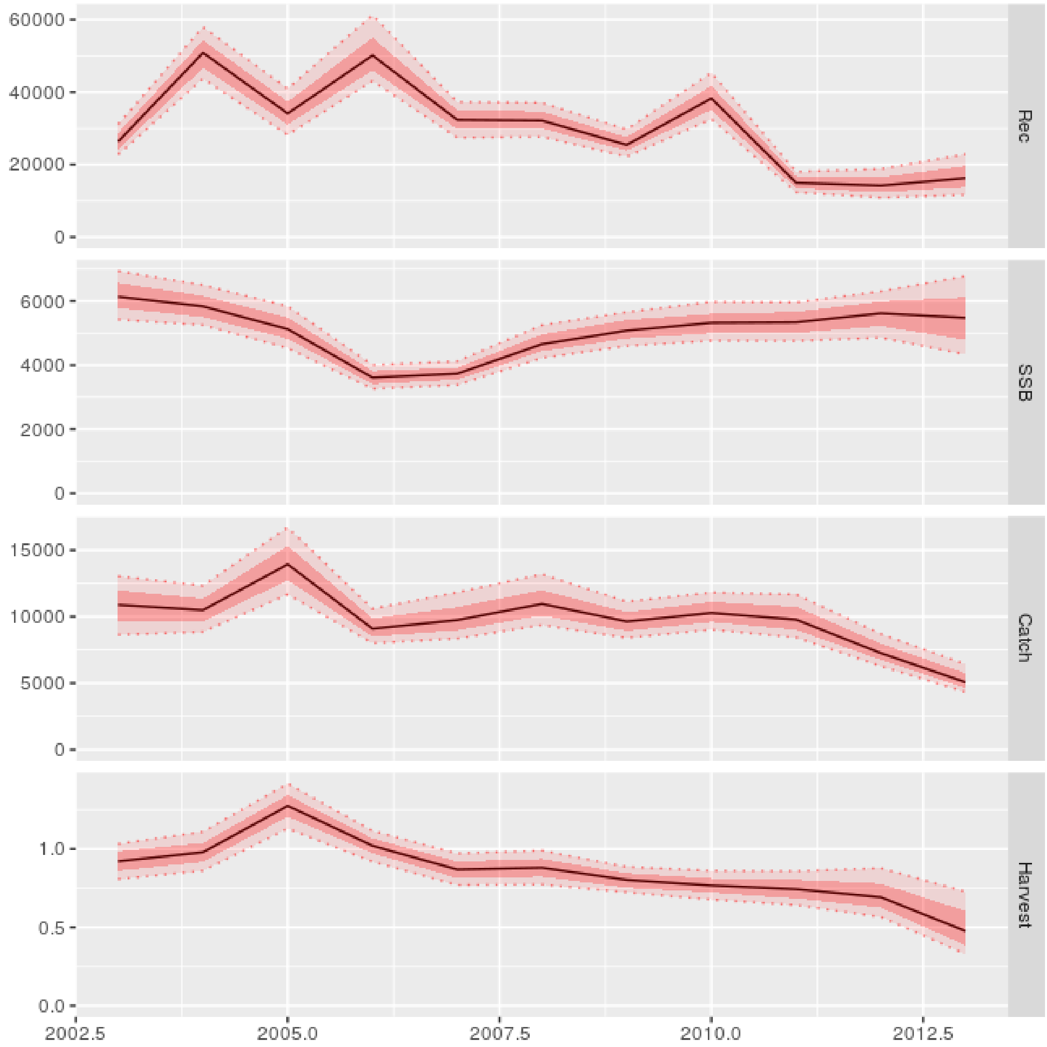

4.4 Skagerrak + viking sub-population

```
stk <- codskvk.stk
ids <- codskvk.ids
ids[[1]] <- trim(ids[[1]], age = 1:5)
ids[[2]] <- trim(ids[[2]], age = 1:4)
fit <- sca(stk, ids, fit = "assessment", fmodel = fmod, qmodel = qmod)
res <- residuals(fit, stk, ids)
plot(res)
```

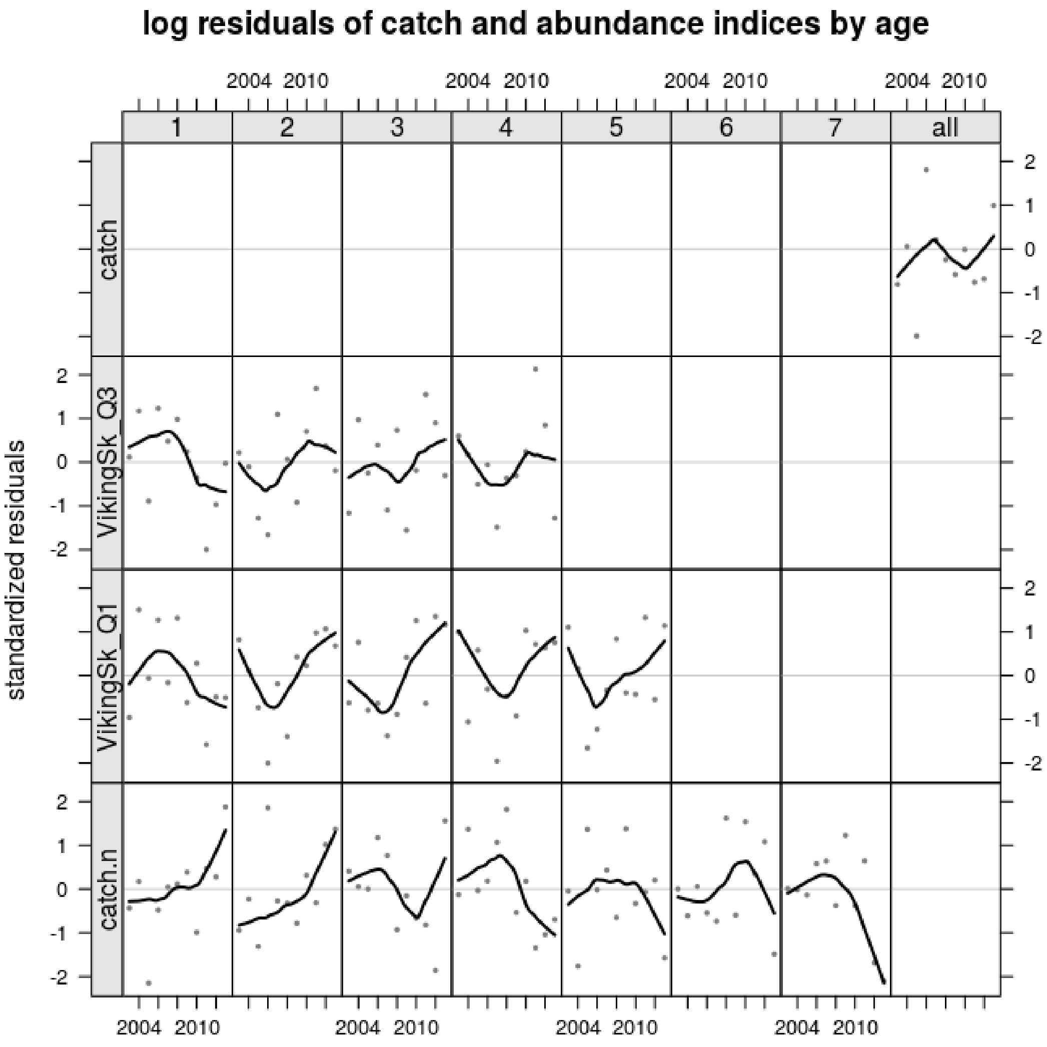


```
wireframe(data ~ year + age, data = harvest(fit))
```

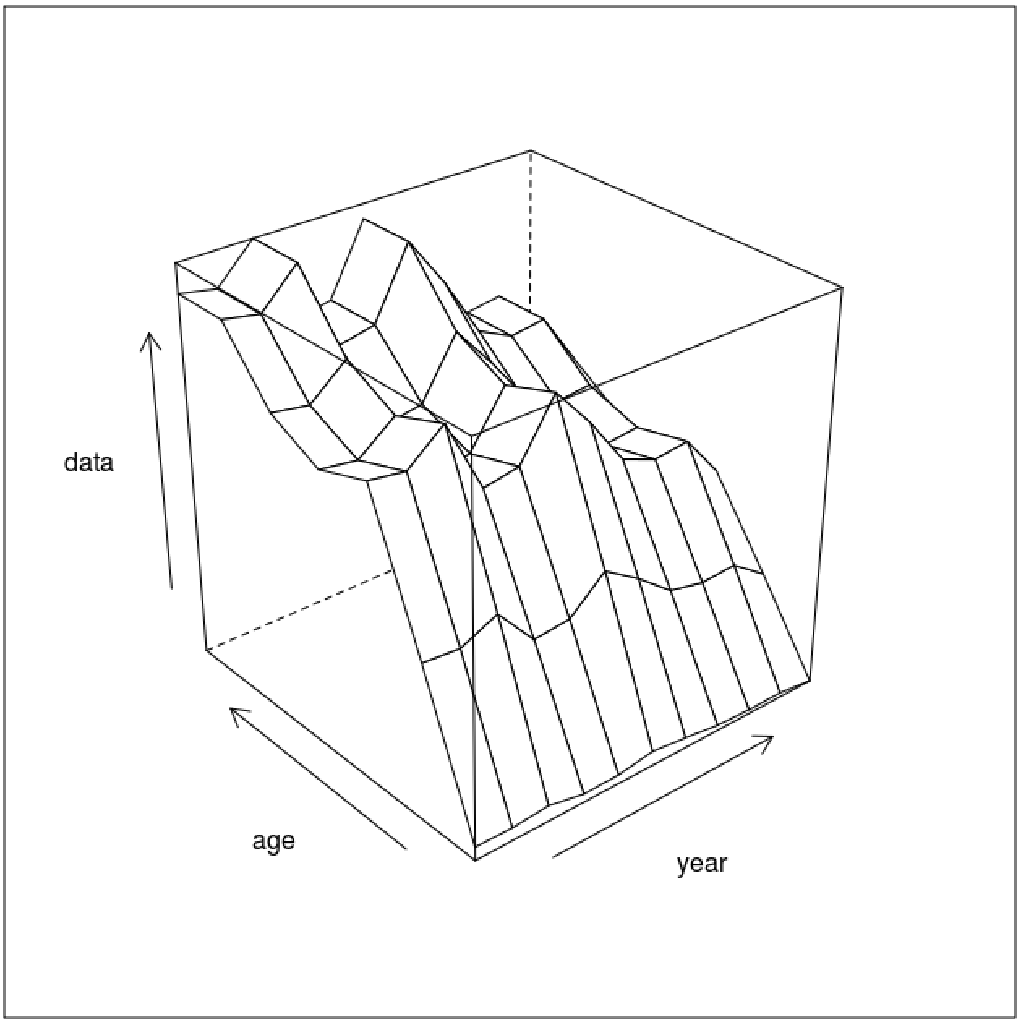


```
# codskvk.fit <- fit
codskvk.fstk <- stk + fit
codskvk.fstks <- stk + a4aSCA(stk, ids, fit = "MCMC", fmodel = fmod, qmodel = qmod,
    mcmc = mc)
plot(codskvk.fstks)
```

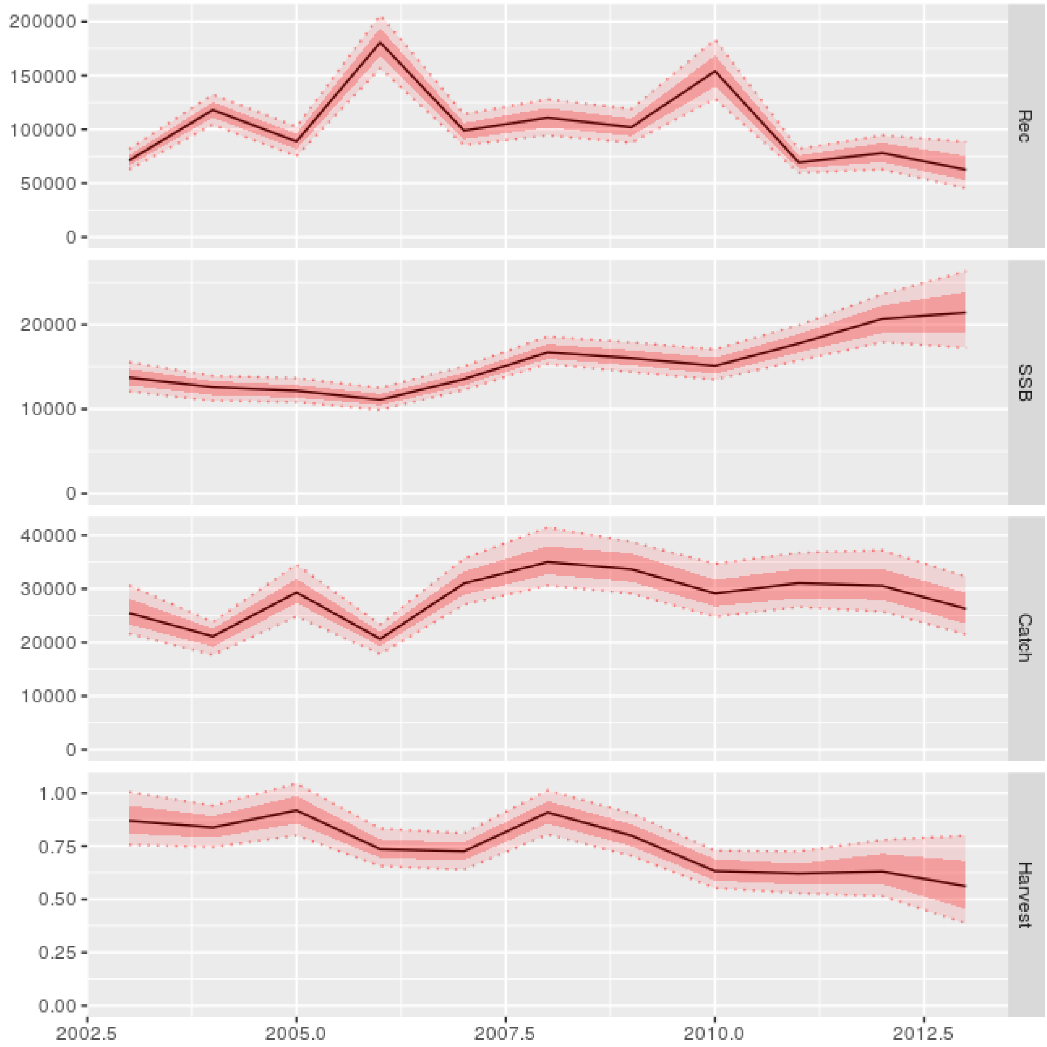
200000
150000
100000
50000
0
Rec
20000
10000
0
SSB
40000
30000
20000
10000
0
Catch
1.00
0.75
0.50
0.25
0.00
Harvest
2002.5
2005.0
2007.5
2010.0
2012.5

5 Comparing tensor with separable fishing mortalities

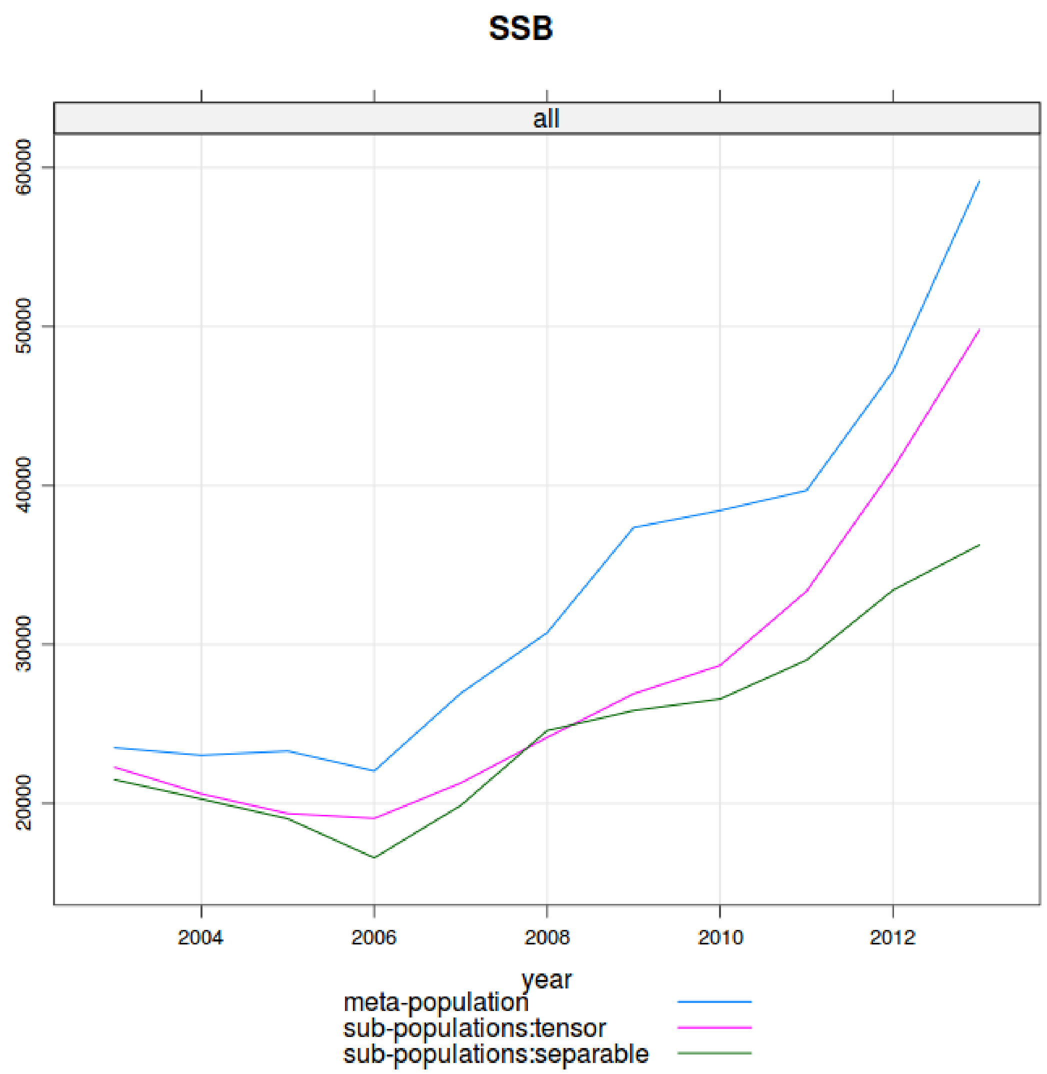

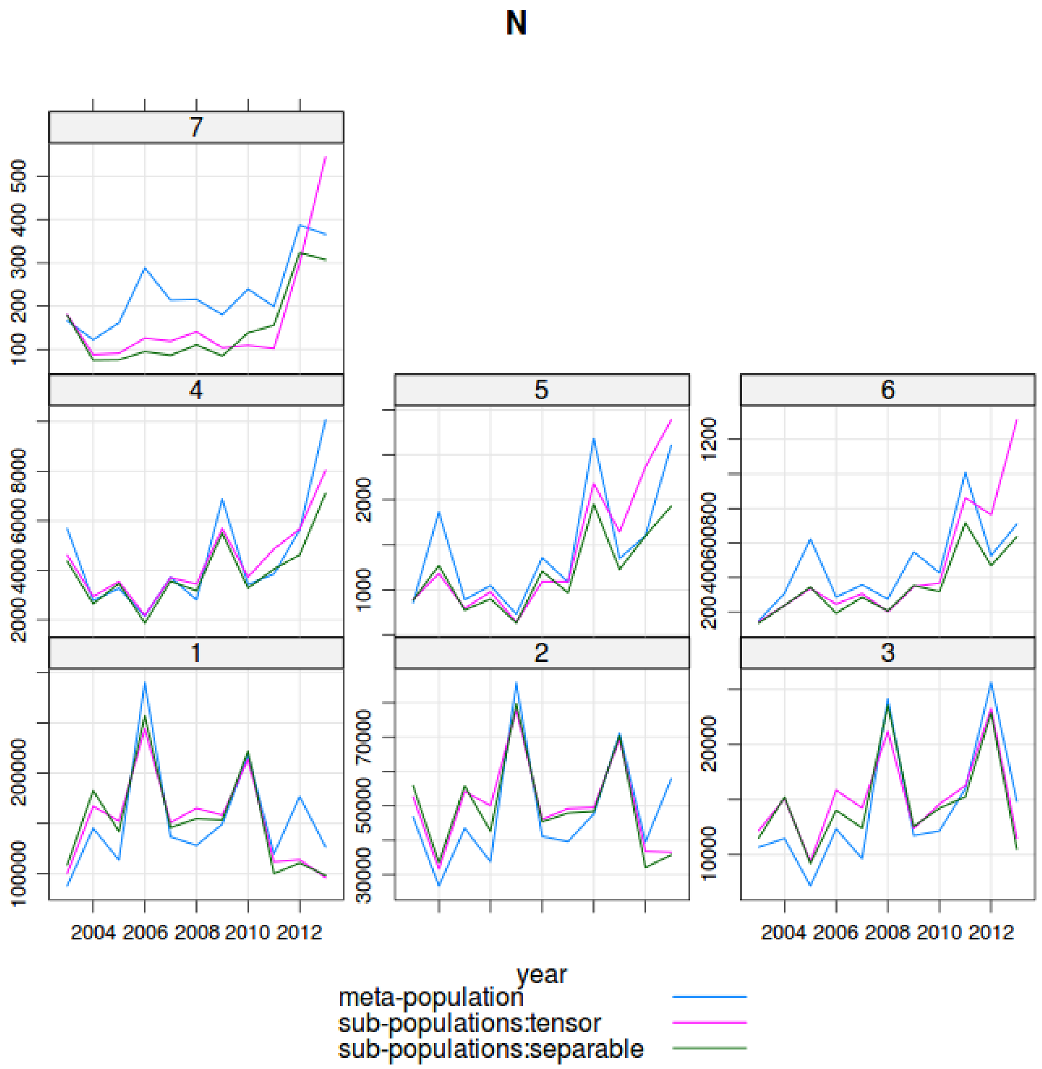

N
7
100 200 300 400 500
4
2000 4000 6000 8000
5
1000
2000
6
200 400 600 800
1200
1
100000
200000
2
30000 50000 70000
3
10000
20000
2004 2006 2008 2010 2012
year
meta-population
sub-populations:tensor
sub-populations:separable

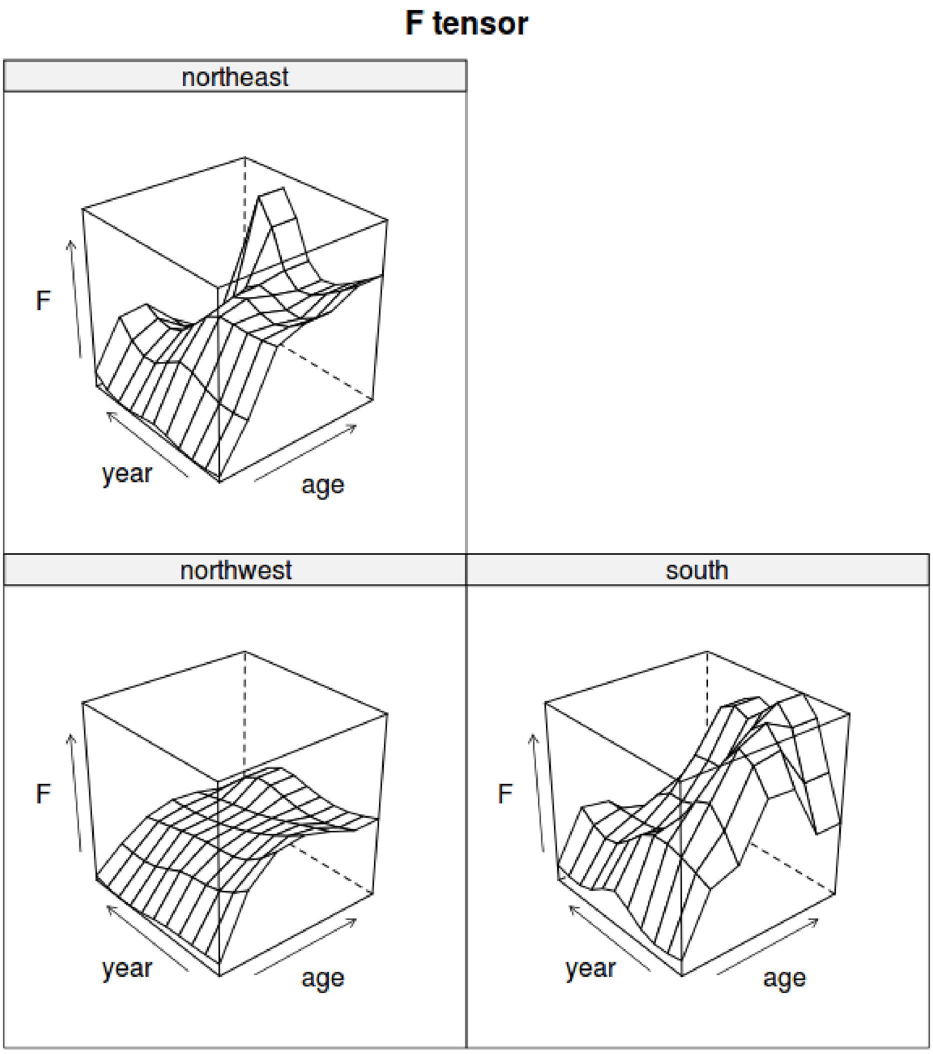
F tensor
northeast
F
year
age
northwest
F
year
age
south
F
year
age

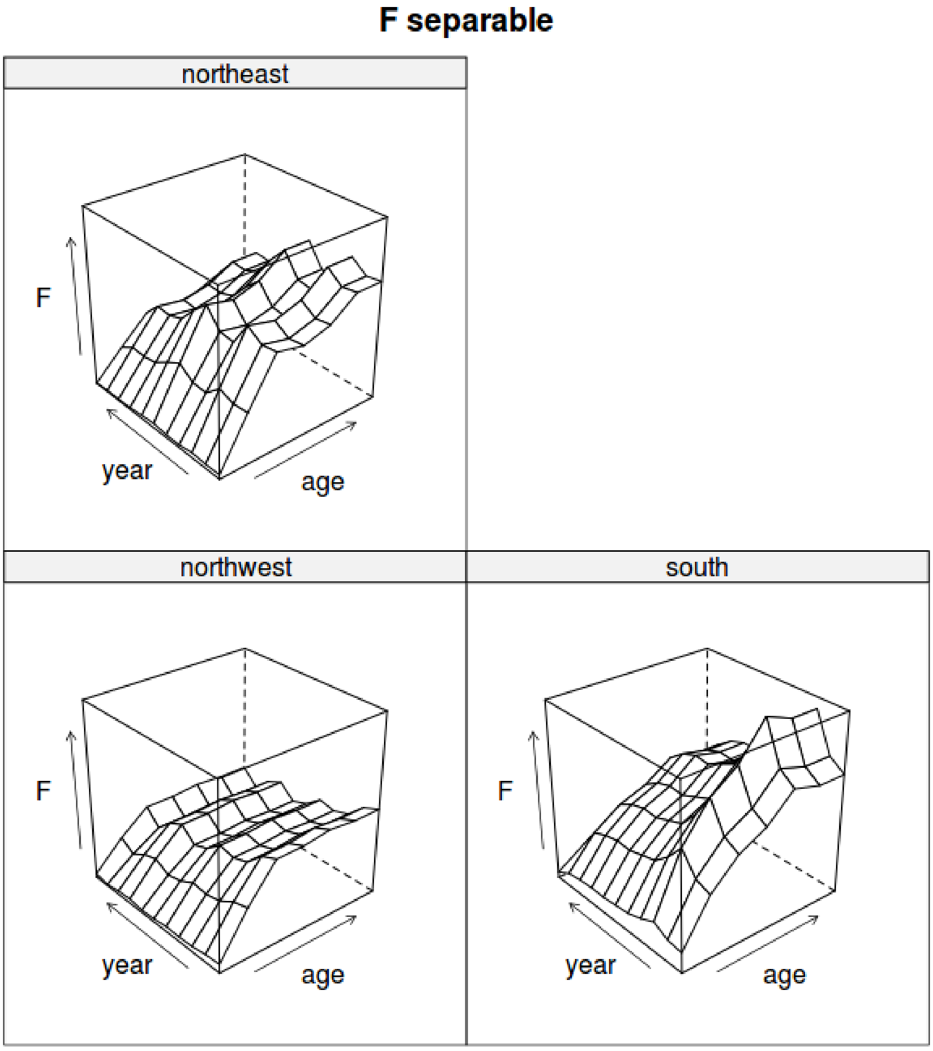
F separable
northeast
F
year
age
northwest
F
year
age
south
F
year
age
