# Supplementary material for: Testing spatial heterogeneity with stock assessment models
Source: PLoS One. 2018 Jan 24;13(1):e0190791. doi: 10.1371/journal.pone.0190791 (PMC5783371; doi:10.1371/journal.pone.0190791)
Supplement: S2 File — (PDF) [file pone.0190791.s005.pdf]

Testing spatial heterogeneity with stock assessment models Appendix II - Northeast Atlantic sardine model sensitivity analysis.

May 11, 2017

Contents

1 Introduction **2**

2 Read data **2**

3 Fitting - "best sub-models" **6**

3.1 Atlantic stock 6

3.2 BB 9

3.3 NW 12

3.4 S 15

4 Fitting - separable **19**

4.1 Atlantic stock 19

4.2 BB 22

4.3 NW 25

4.4 S 28

5 Comparing tensor with separable fishing mortalities **31**

1 Introduction

This document shows the results from assessments made to different spatial aggregations of the Atlantic sardine stock data:

- single **Atlantic stock (A)** from the North Bay of Biscay to the Gulf of Cadiz, and
- three stocks representing three putative non-overlapping sub-populations,
  - **Bay of Biscay or BoB (BB)**: ICES sub-divisions VIIIa, VIIIb and [the eastern half of sub-division VIIIc].
  - **Northwest (NW)**: The western half of ICES sub-division VIIIc and that part of ICES sub-division IXa on the west coast of Portugal.
  - **South (S)**: That part of ICES sub-division IXa off the south coasts of Portugal and Spain.

The input data for assessments consisted in:

- annual catch biomass (in tons)
- numbers-at-age in the catch (in thousand individuals)
- mean weight–at-age in the catch (in kg)
- an index of abundance-at-age (in thousand individuals) from annual spring acoustic surveys
- an index of biomass (in tons) from a triennial Daily Egg Production Method (DEPM) survey in the case of NW and S stocks and an index of total egg abundance from an annual DEPM survey (BB stock)
- maturity-at-age (in proportions)
- mean weight-at-age in the stock (in kg)

Fishery and survey data were available for the BB.

For the NW and S stocks, data on the fishery, maturity-at-age and weights-at-age span the period 1991-2014. Data from acoustic and DEPM surveys span from 1996 and 1997 to 2014, respectively. The datasets for the NW and S stocks were compiled from data disaggregated into seven ICES Divisions reported annually to the working groups for the assessment of the Atlantic-Iberian sardine stock. The NW stock comprise Divisions 8.c (South Bay of Biscay), 9.a.n (South Galicia), 9a.CN (North Portugal) and 9.a.CS (South-West Portugal) and the South stock comprise Divisions 9.a.Sa (South-Portugal) and 9.a.Sc (Gulf of Cadiz). Data by ICES Division were summed (in the case of catch and abundance) or weighted-averaged (in the case of mean weights and maturity ogives) to obtain the datasets for the NW and S stocks. The catch-at-age and abundance-at-age pooled from the NW and S showed some differences, generally below 5%, to the dataset used in the assessment of the Atlantic-Iberian stock.

For the Atlantic stock, A, the assessment period becomes 2002 – 2014.

Data by age are aggregated in a 6+ group.

For each stock definition a comparison was made between two different assessment approaches; a separable model for fishing mortality and a time/age variant model (tensor). Using both the tensor seperable modelling approaches the sum of the SSBs from assessments on the three sub-populations and the SSB obtained by fitting to data over the whole area were comparable.

The consistent SSB results, between whole stock and sub-population assessments and between tensor and separable methodologies, lead the study to conclude the three sub-population scenario was robust to model uncertainty.

2 Read data

```
# BB
load("BBdata.RData")
BB.stk <- setPlusGroup(BB.stk, 6)
BB.idx[[1]] <- FLIndex(index = setPlusGroup(index(BB.idx[[1]]), 6, by = "sum"))
range(BB.idx[[1]])[c("min", "max", "startf", "endf")] <- c(1, 6, 4.8/12, 6/12)
range(BB.idx[[2]])[c("min", "max", "startf", "endf")] <- c(1, 6, 5.1/12, 5.8/12)
mat(BB.stk)[2, ] <- 1
m.spwn(BB.stk) <- 0
harvest.spwn(BB.stk) <- 0
range(BB.stk)[c("minfbar", "maxfbar")] <- c(2, 5)

# NW
load("NW.Rdata")
# correct the NW.idx and save it
index(NW.idx[[1]])[, 9] <- NA
index(NW.idx[[1]])[, 17] <- NA

# NW NW.stk <- merge(N.stk, W.stk) m(NW.stk) <- m(N.stk) # m is the same for
# N and W mat(NW.stk) <- mat(N.stk) # m is the same for N and W
# stock.wt(NW.stk) <- (stock.wt(N.stk) + stock.wt(W.stk))/2 NW.idx <- N.idx
# NW.idx[[1]]@index <- (N.idx[[1]]@index + W.idx[[1]]@index)/2
# NW.idx[[2]]@index <- (N.idx[[2]]@index + W.idx[[2]]@index)/2

# S
load("S.RData")

# IB
load("IB.Rdata")

# A(ll)

#### Trimming ##########################
yearsel = 2002:2014
BB.temp <- trim(BB.stk, year = yearsel)
IB.temp <- trim(IB.stk, year = yearsel)
NW.temp <- trim(NW.stk, year = yearsel)
S.temp <- trim(S.stk, year = yearsel)

#### Building A.stk object ############
A.stk <- FLStock()
name(A.stk) <- "ALL REGIONS ATLANTO-IBERIAN SARDINE"
desc(A.stk) <- "prepared for a4a workshop - 15/12/2015"
A.stk@range <- IB.temp@range
A.stk@m.spwn <- IB.temp@m.spwn
A.stk@harvest.spwn <- IB.temp@harvest.spwn
A.stk@harvest <- BB.temp@harvest
A.stk@mat <- S.temp@mat
A.stk@m <- S.temp@m
A.stk@discards <- BB.temp@discards
A.stk@discards.wt <- BB.temp@discards.wt
A.stk@discards.n <- BB.temp@discards.n
A.stk@landings <- BB.temp@landings + S.temp@landings * 1000 + NW.temp@landings *
    1000
units(A.stk@landings) <- "tons"
A.stk@landings.n <- BB.temp@landings.n + S.temp@landings.n * 1000 + NW.temp@landings.n *
    1000
units(A.stk@landings.n) <- "thousands"
A.stk@catch <- A.stk@landings
```

```
A.stk@catch.n <- A.stk@landings.n
A.stk@landings.wt <- (BB.temp@catch.wt * BB.temp@landings.n + NW.temp@catch.wt *
    NW.temp@landings.n * 1000 + S.temp@catch.wt * S.temp@landings.n * 1000)/A.stk@landings.n
units(A.stk@landings.wt) <- "kg"
A.stk@catch.wt <- A.stk@landings.wt
A.stk@stock <- S.temp@stock
A.stk@stock.n <- S.temp@stock.n
A.stk@stock.wt <- (BB.temp@stock.wt * BB.temp@landings.n + NW.temp@stock.wt *
    NW.temp@landings.n * 1000 + S.temp@stock.wt * S.temp@landings.n * 1000)/A.stk@landings.n
units(A.stk@stock.wt) <- "kg"

#### Building A.idx object ############
BB1 <- trim(BB.idx[[1]], year = yearsel, age = 1:6)
BB2 <- trim(BB.idx[[2]], year = yearsel, age = 1:6)
IB1 <- trim(IB.idx[[1]], year = yearsel, age = 1:6)
IB2 <- trim(IB.idx[[2]], year = yearsel, age = 1:6)
NW1 <- trim(NW.idx[[1]], year = yearsel, age = 1:6)
NW2 <- trim(NW.idx[[2]], year = yearsel, age = 1:6)
S1 <- trim(S.idx[[1]], year = yearsel, age = 1:6)
S2 <- trim(S.idx[[2]], year = yearsel, age = 1:6)
desc(BB2) <- "BB Egg"
desc(BB1) <- "BB Acoustic"
desc(IB1) <- "IB Acoustic"
desc(IB2) <- "IB DEPM"
desc(NW1) <- "NW Acoustic"
desc(NW2) <- "NW DEPM"
desc(S1) <- "S Acoustic"
desc(S2) <- "S DEPM"
name(BB2) <- "BB Egg"
name(BB1) <- "BB Acoustic"
name(IB1) <- "IB Acoustic"
name(IB2) <- "IB DEPM"
name(NW1) <- "NW ACoustic"
name(NW2) <- "NW DEPM"
name(S1) <- "S Acoustic"
name(S2) <- "S DEPM"

#### Separate survey file ############

### Separate object file ###
NW1@index[, 3] <- (NW1@index[, 2] + NW1@index[, 4])/2
NW1@index[, 11] <- (NW1@index[, 10] + NW1@index[, 12])/2
S1@index[, 3] <- (S1@index[, 2] + S1@index[, 4])/2
S1@index[, 11] <- (S1@index[, 10] + S1@index[, 12])/2

#### AC: Aggregated acoustic index ####
AC <- NW1
name(AC) <- "Aggr Acoustic"
desc(AC) <- "Aggr Acoustic"
AC@index <- BB1@index + NW1@index * 1000 + S1@index * 1000
AC@range <- BB1@range
units(AC@index) <- "Thousands"

#### DP: Aggregated DEPM index ########
DP <- NW2
name(DP) <- "Aggr DEPM"
desc(DP) <- "Aggr DEPM"
DP@index <- NW2@index * 1000 + S2@index * 1000
```

```r
DP@range <- NW2@range
units(DP@index) <- "Thousands"
DP@index[, 2] <- (DP@index[, 1] * 2 + DP@index[, 4] * 1)/3

A.idx <- FLIndices(AC, BB2, DP)
```

3 Fitting - "best sub-models"

```
# sub-models
fmod1a <- ~te(age, year, k = c(5, 6))  # Ks/data = 0.329
fmod1b <- ~te(age, year, k = c(6, 6))  # Ks/data = 0.395
fmod2a <- ~te(age, year, k = c(5, 11))  # Ks/data = 0.327
fmod2b <- ~te(age, year, k = c(6, 11))  # Ks/data = 0.392
qmod1 <- list(~factor(replace(age, age > 1, 2)) + age, ~year, ~1)
qmod2 <- list(~factor(replace(age, age > 1, 2)) + age, ~1)
# tested: lowest BIC when k=11 for srmodel
rmod1 <- ~s(year, k = 11)
rmod2 <- ~s(year, k = 18)
n1 <- ~s(age, k = 4)
# mcmc settings
mc <- SCAMCMC(mcmc = 125000, mcsave = 250, mcprobe = 0.4, mcrb = 3)
```

3.1 Atlantic stock

```
stk <- A.stk
ids <- A.idx
fit <- a4aSCA(stk, ids, fmodel = fmod1a, qmodel = qmod1, n1model = n1, srmodel = rmod1)
res <- residuals(fit, stk, ids)
plot(res)
```

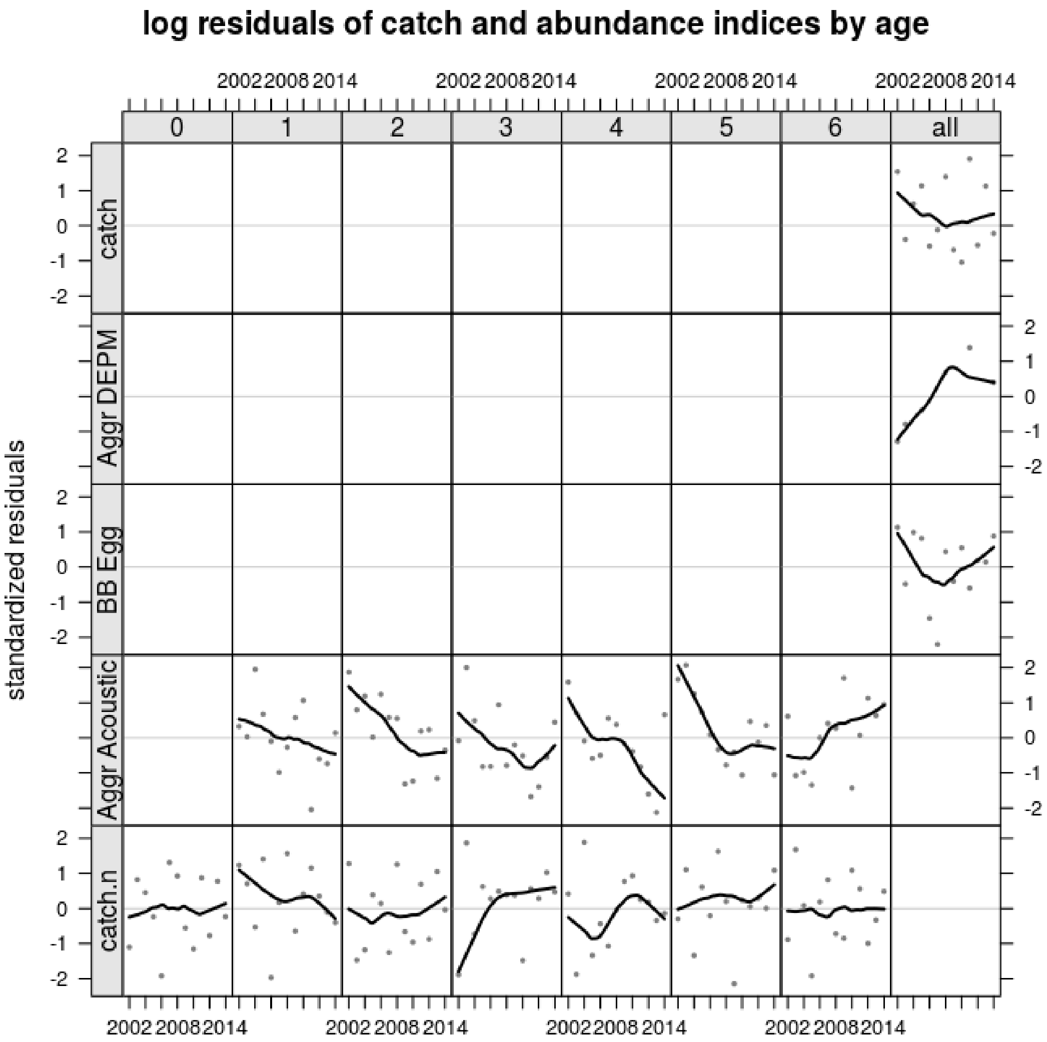


```r
wireframe(data ~ year + age, data = harvest(fit))
```

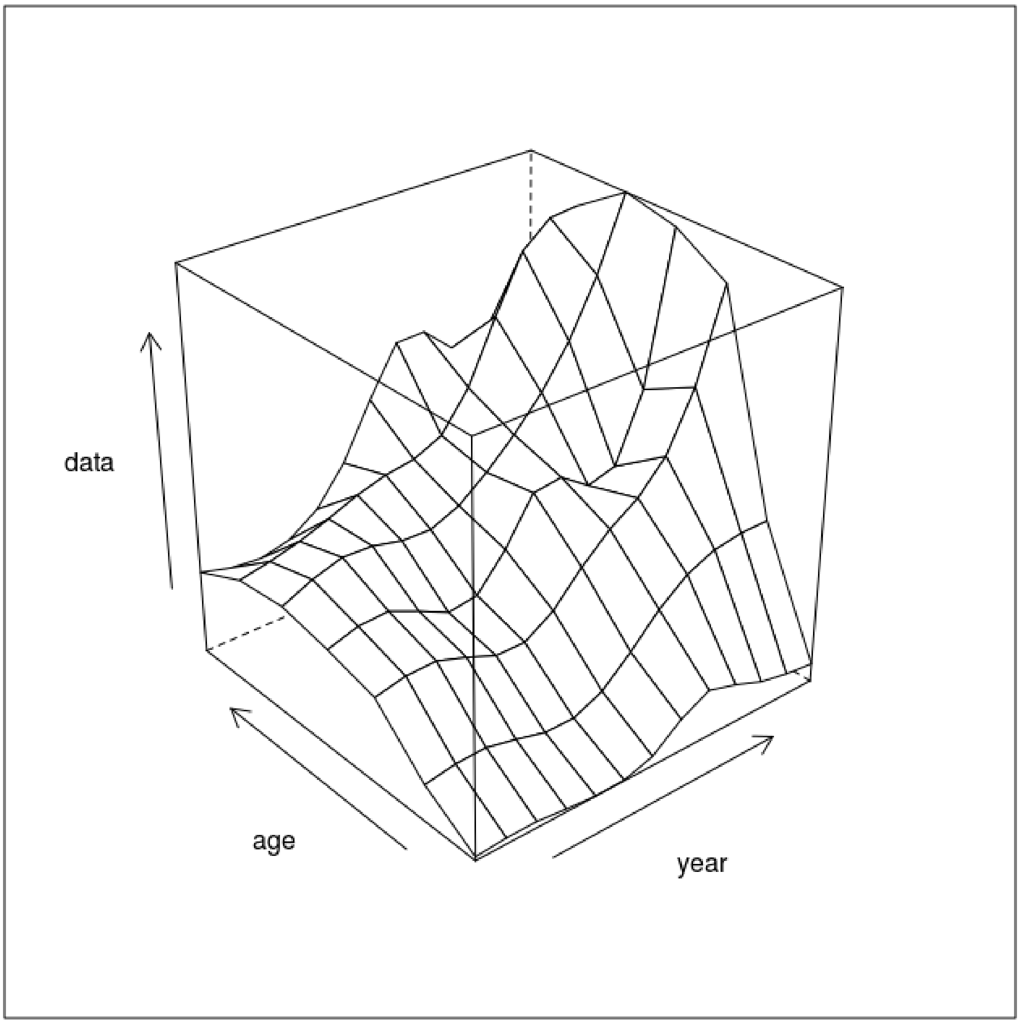


```
pil.fit <- fit
pil.fstk <- stk + fit
pil.fstks <- stk + a4aSCA(stk, ids, fit = "MCMC", fmodel = fmod1a, qmodel = qmod1,
    n1model = n1, srmodel = rmod1, mcmc = mc)
plot(pil.fstks)
```

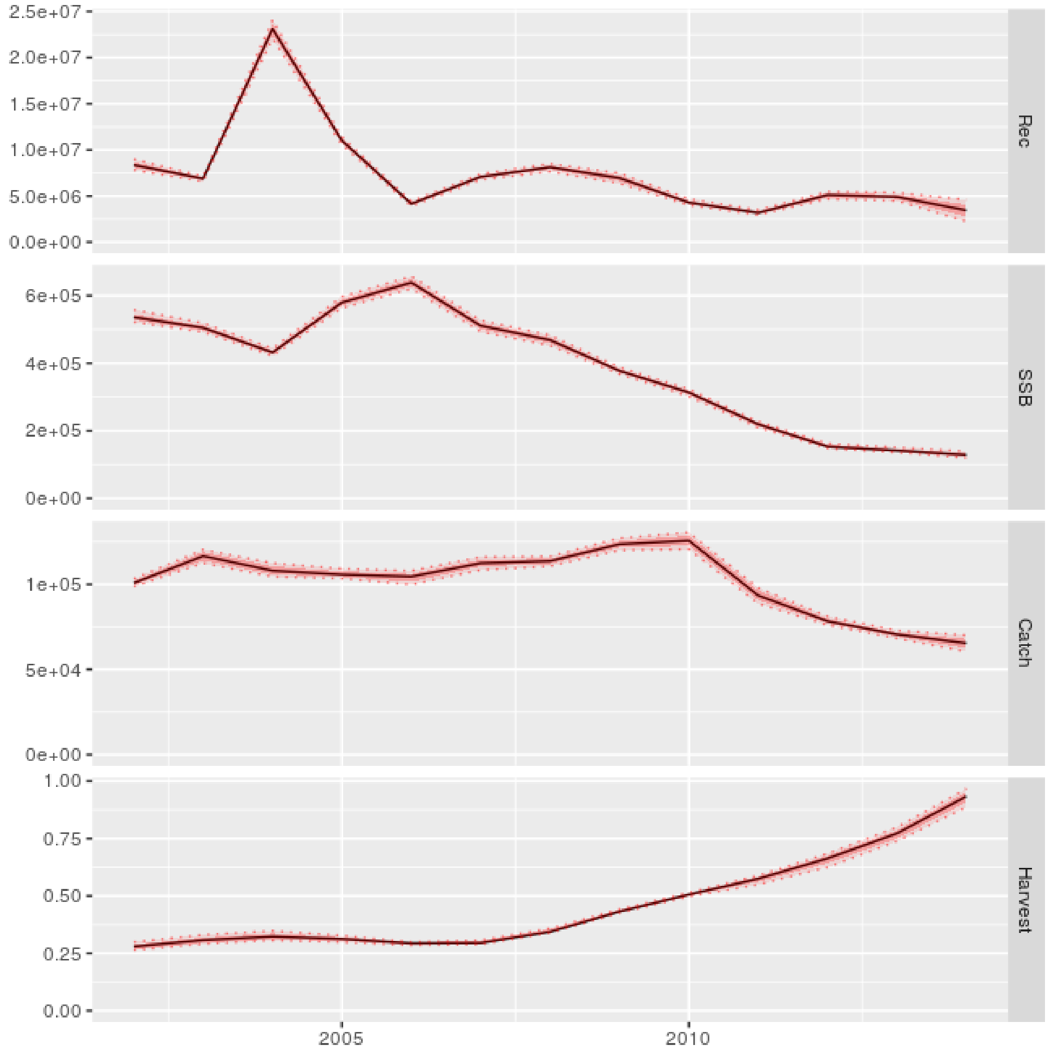

3.2 BB

```
stk <- BB.stk
ids <- BB.idx
fit <- a4aSCA(stk, ids, fmodel = fmod1b, qmodel = qmod2, n1model = n1, srmodel = rmod1)
res <- residuals(fit, stk, ids)
plot(res)
```

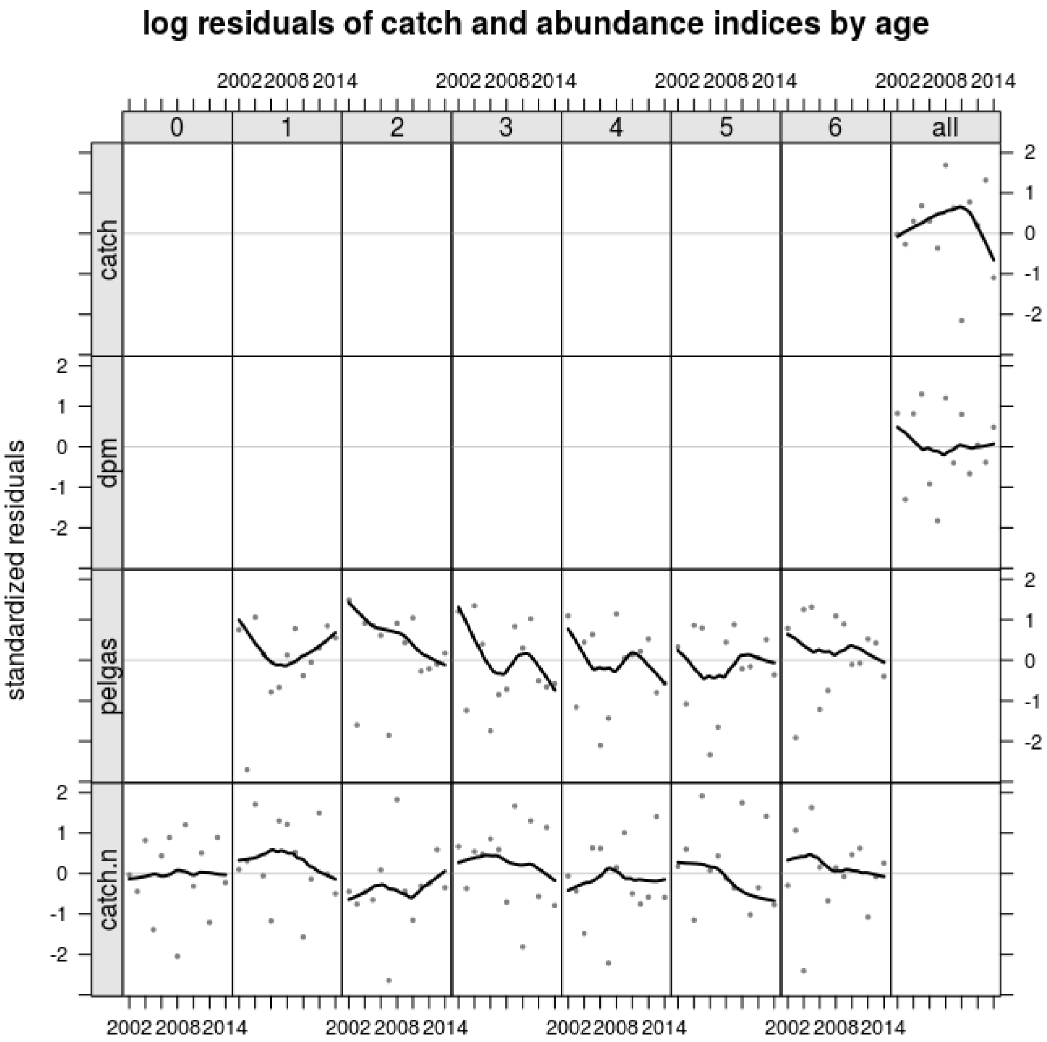


```
wireframe(data ~ year + age, data = harvest(fit))
```

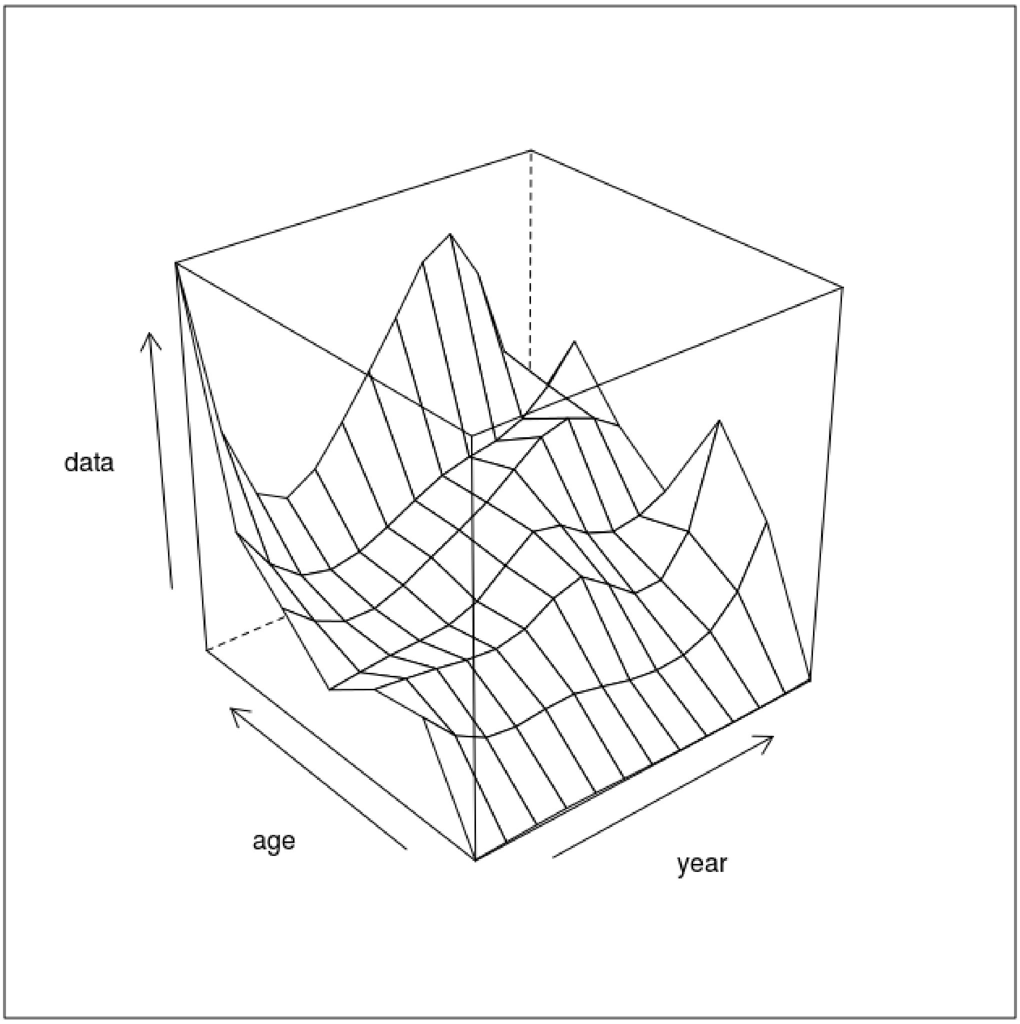


```
pilbb.fit <- fit
pilbb.fstk <- stk + fit
pilbb.fstks <- stk + a4aSCA(stk, ids, fit = "MCMC", fmodel = fmod1b, qmodel = qmod2,
    n1model = n1, srmodel = rmod1, mcmc = mc)
plot(pilbb.fstks)
```

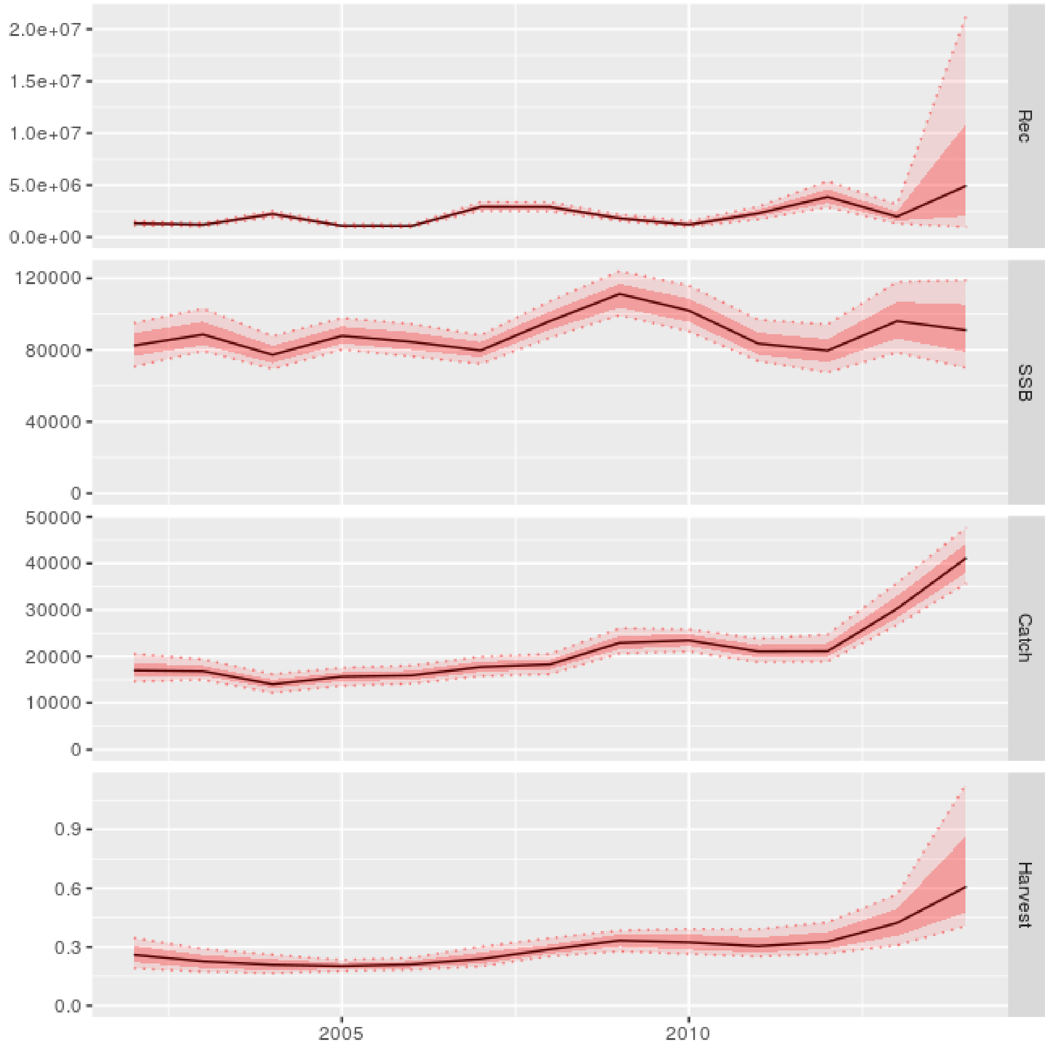

3.3 NW

```
stk <- NW.stk
ids <- NW.idx
fit <- a4aSCA(stk, ids, fmodel = fmod2a, qmodel = qmod2, n1model = n1, srmodel = rmod2)
res <- residuals(fit, stk, ids)
plot(res)
```

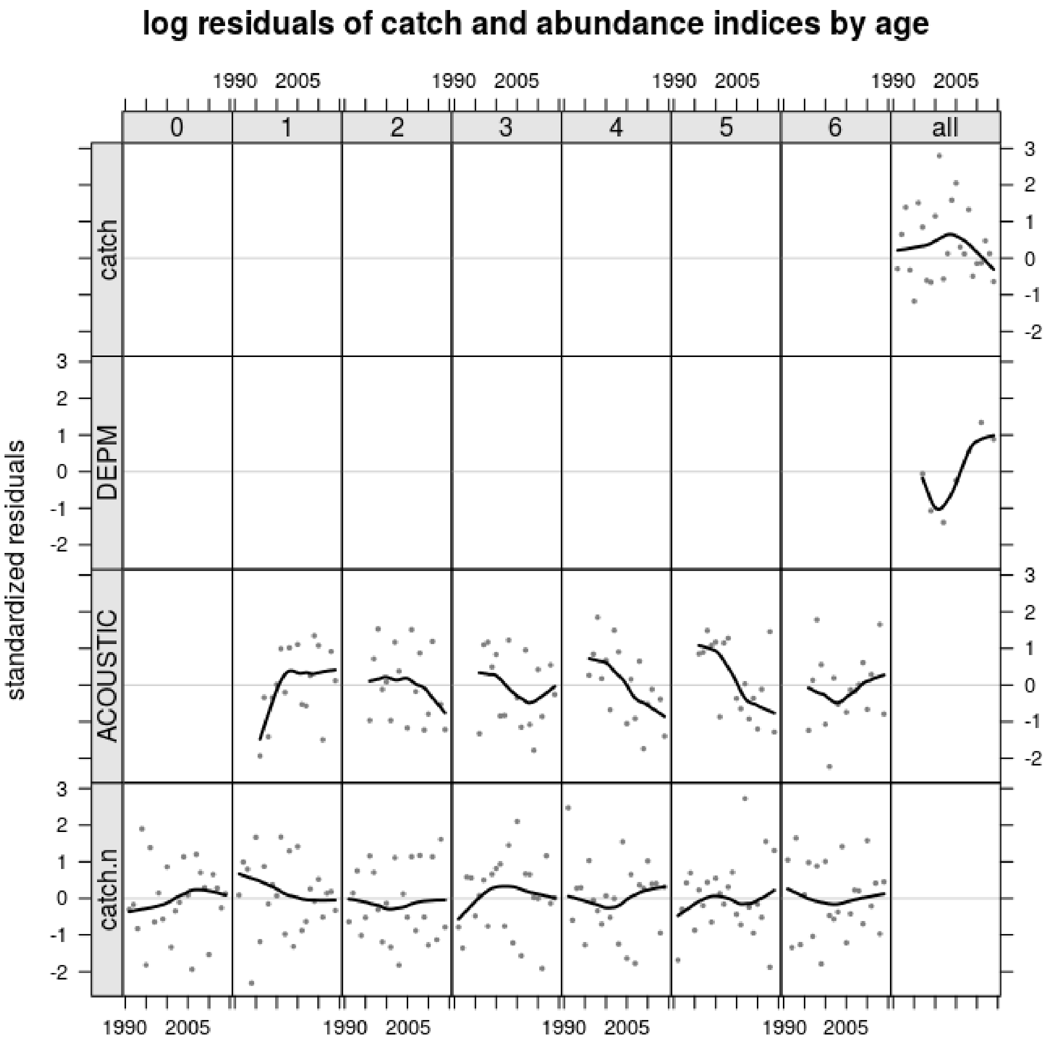


```r
wireframe(data ~ year + age, data = harvest(fit))
```

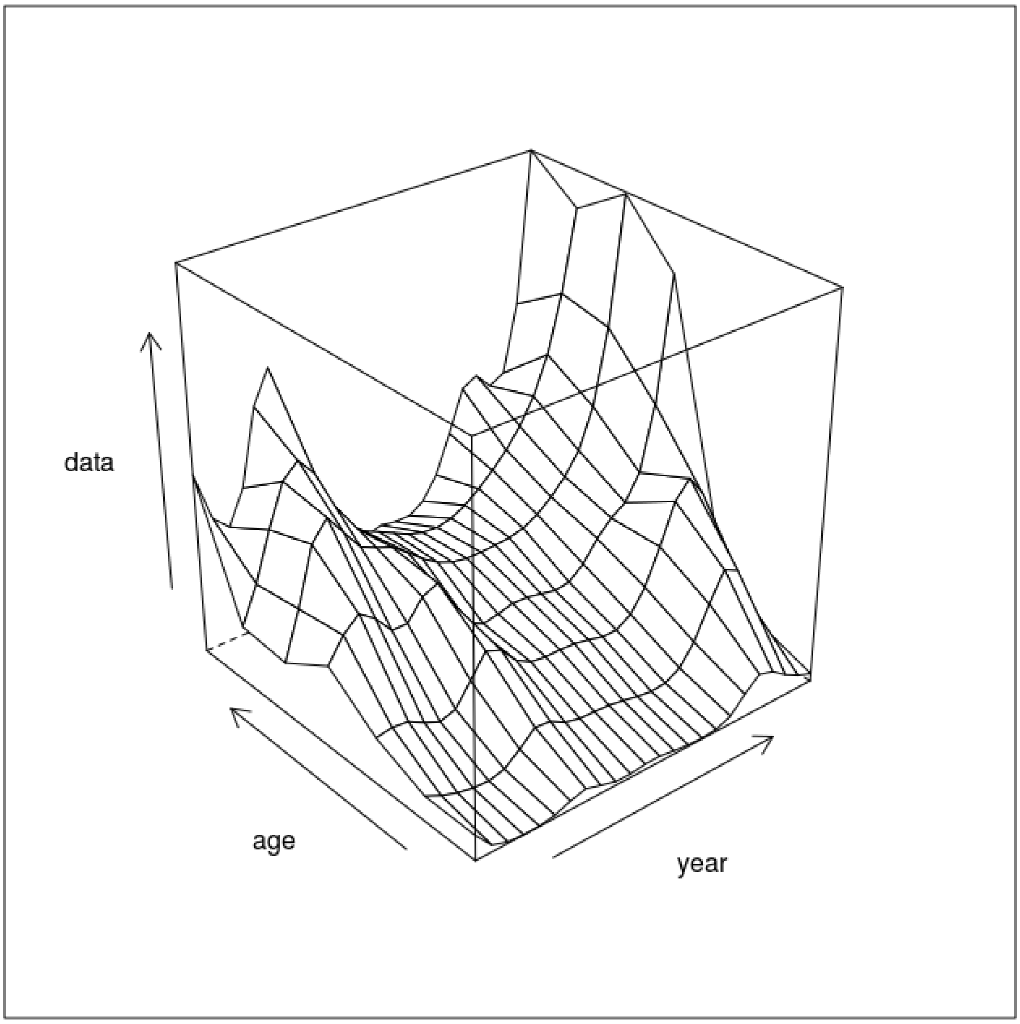


```
pilnw.fit <- fit
pilnw.fstk <- stk + fit
pilnw.fstks <- stk + a4aSCA(stk, ids, fit = "MCMC", fmodel = fmod2a, qmodel = qmod2,
    n1model = n1, srmodel = rmod2, mcmc = mc)
plot(pilnw.fstks)
```

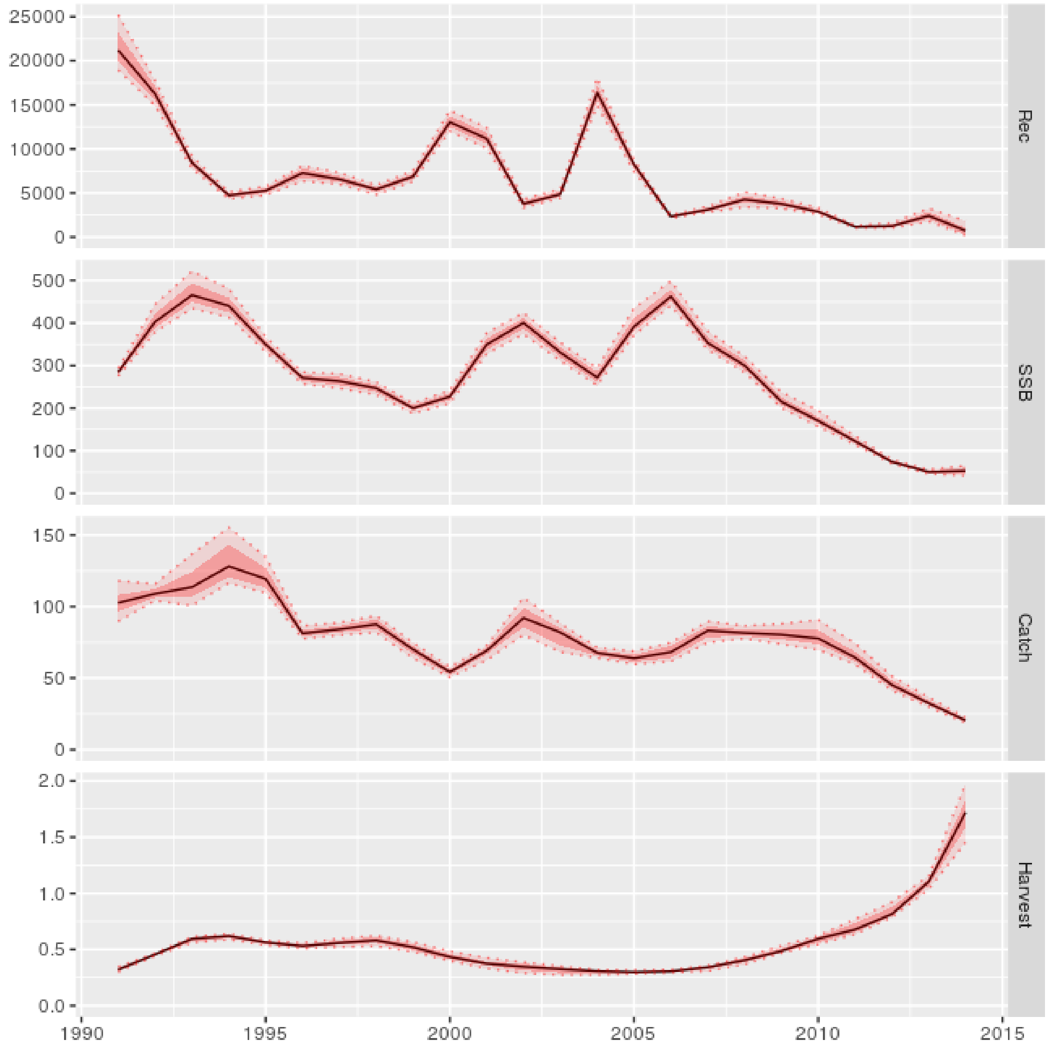

3.4 S

```
stk <- S.stk
ids <- S.idx
fit <- a4aSCA(stk, ids, fmodel = fmod2a, qmodel = qmod2, n1model = n1, srmodel = rmod2)
res <- residuals(fit, stk, ids)
plot(res)
```

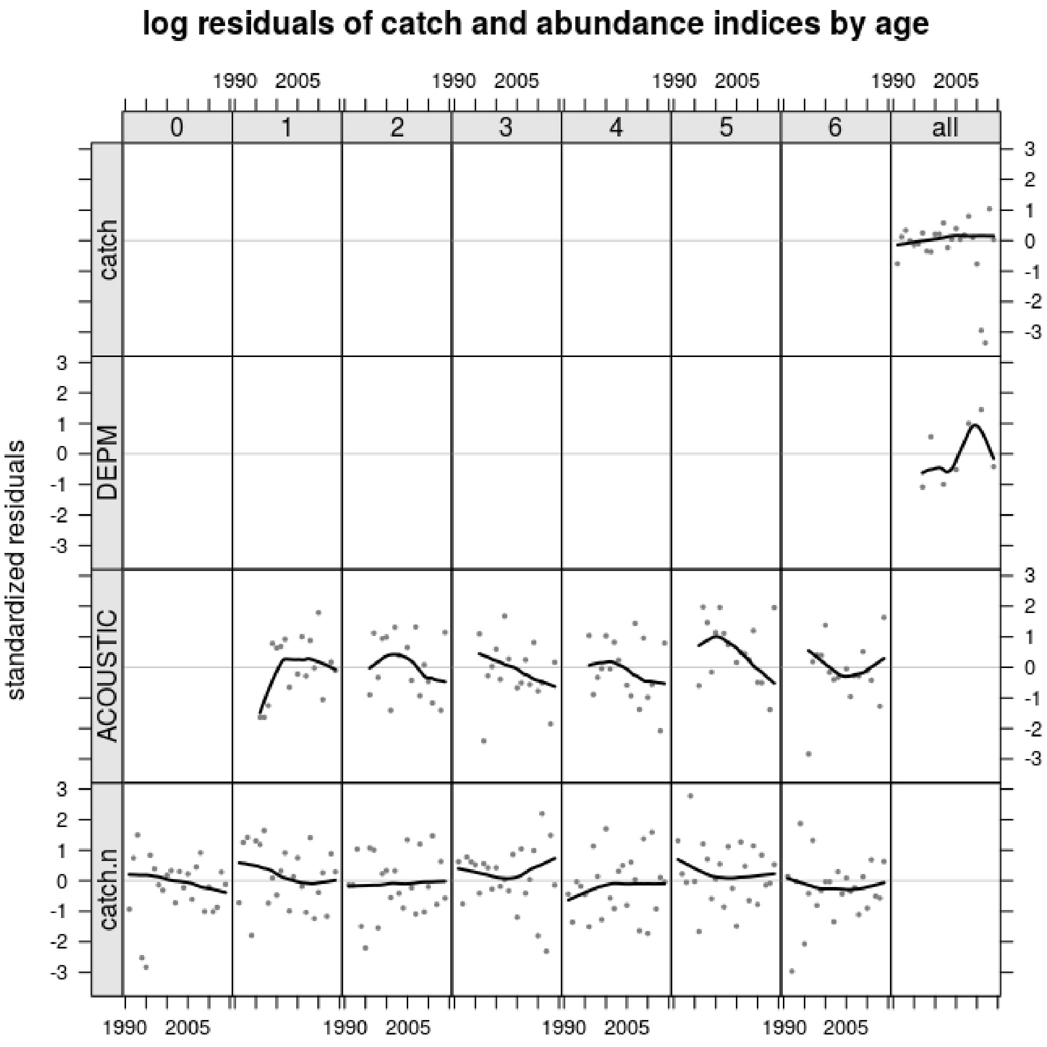


```
wireframe(data ~ year + age, data = harvest(fit))
```

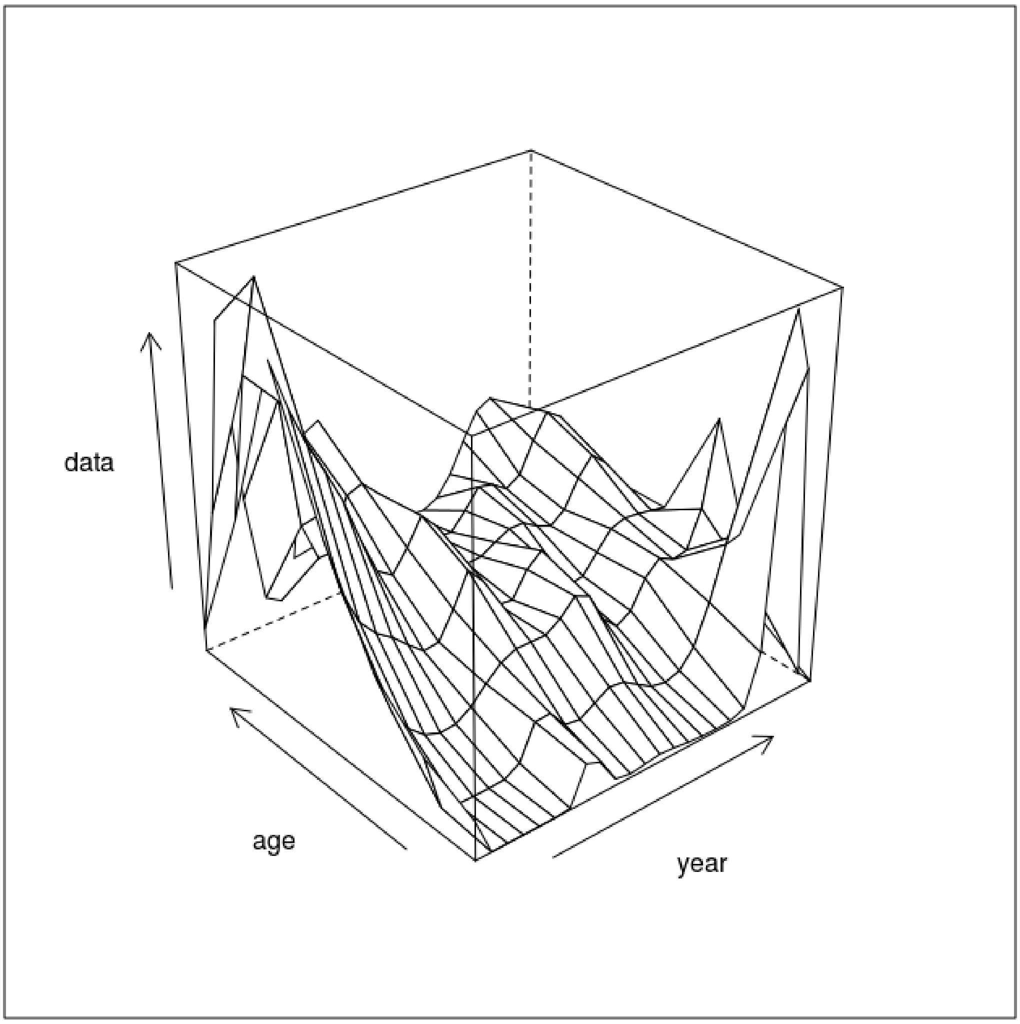


```
pilso.fit <- fit
pilso.fstk <- stk + fit
pilso.fstks <- stk + a4aSCA(stk, ids, fit = "MCMC", fmodel = fmod2a, qmodel = qmod2,
    n1model = n1, srmodel = rmod2, mcmc = mc)
plot(pilso.fstks)
```

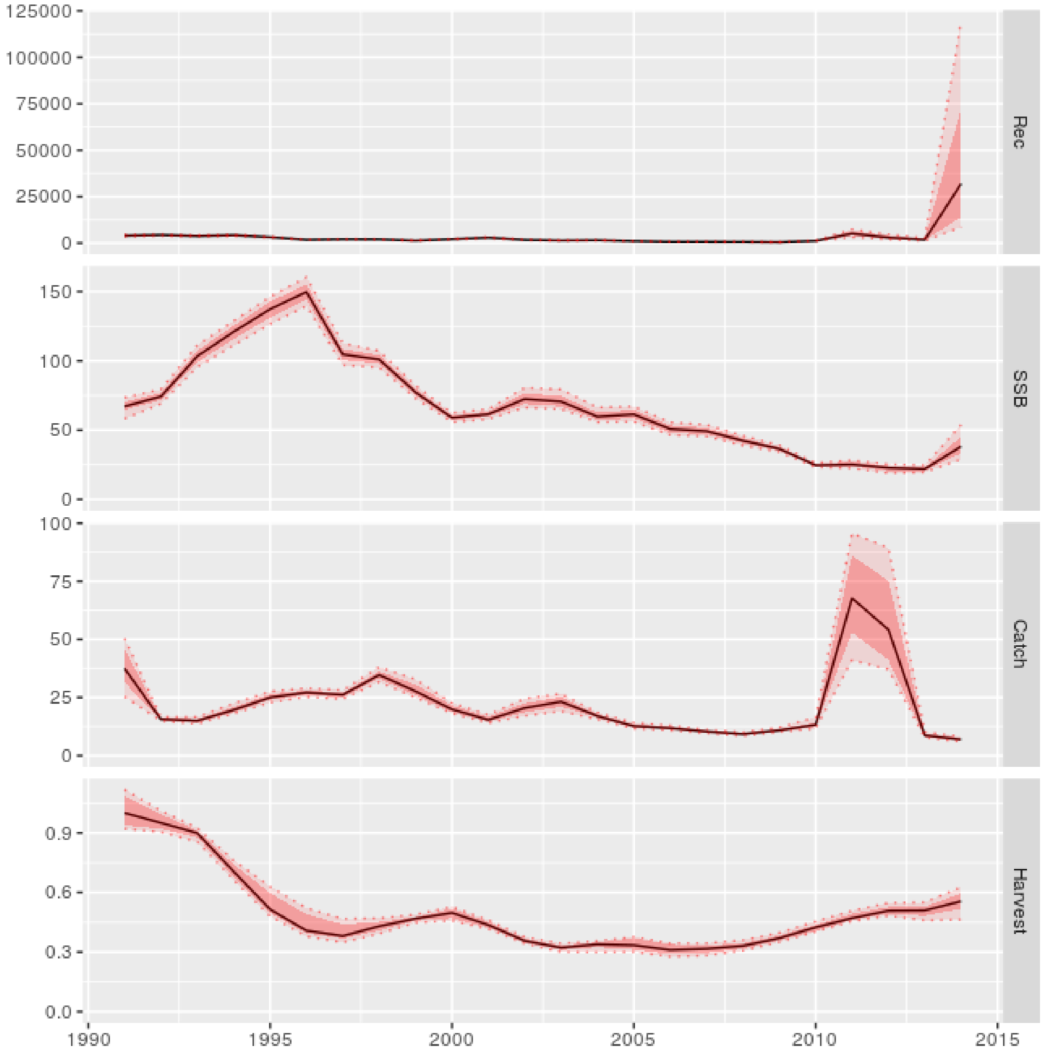


```
pil3ssb <- ssb(window(pilnw.fstk, start = 2002)) * 1000 + ssb(window(pilso.fstk,
    start = 2002)) * 1000 + ssb(pilbb.fstk)
ssb.flqs <- FLQuants(`meta-population` = ssb(pil.fstk), `sub-populations:tensor` = pil3ssb)

pil3n <- stock.n(window(pilnw.fstk, start = 2002)) * 1000 + stock.n(window(pilso.fstk,
    start = 2002)) * 1000 + stock.n(pilbb.fstk)
n.flqs <- FLQuants(`meta-population` = stock.n(pil.fstk), `sub-populations:tensor` = pil3n)
```

4 Fitting - separable

```
# sub-models
fmod1a <- ~factor(age) + s(year, k = 6)
fmod1b <- ~factor(age) + s(year, k = 6)
fmod2a <- ~factor(age) + s(year, k = 11)
fmod2b <- ~factor(age) + s(year, k = 11)
qmod1 <- list(~factor(replace(age, age > 1, 2)) + age, ~year, ~1)
qmod2 <- list(~factor(replace(age, age > 1, 2)) + age, ~1)
# tested: lowest BIC when k=11 for srmodel
rmod1 <- ~s(year, k = 11)
rmod2 <- ~s(year, k = 18)
n1 <- ~s(age, k = 4)
# mcmc settings
mc <- SCAMCMC(mcmc = 125000, mcsave = 250, mcprobe = 0.4, mcrb = 3)
```

4.1 Atlantic stock

```
stk <- A.stk
ids <- A.idx
fit <- a4aSCA(stk, ids, fmodel = fmod1a, qmodel = qmod1, n1model = n1, srmodel = rmod1)
res <- residuals(fit, stk, ids)
plot(res)
```

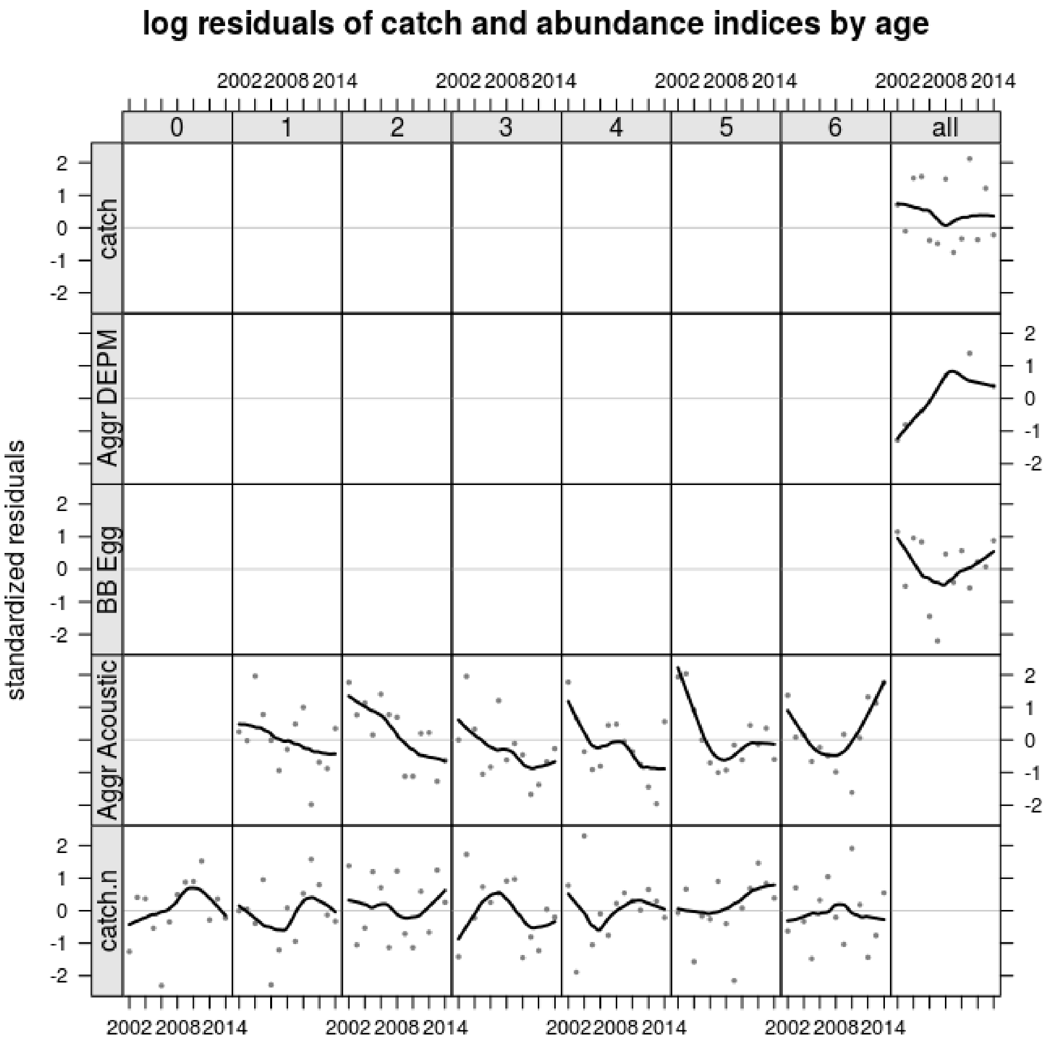


```r
wireframe(data ~ year + age, data = harvest(fit))
```

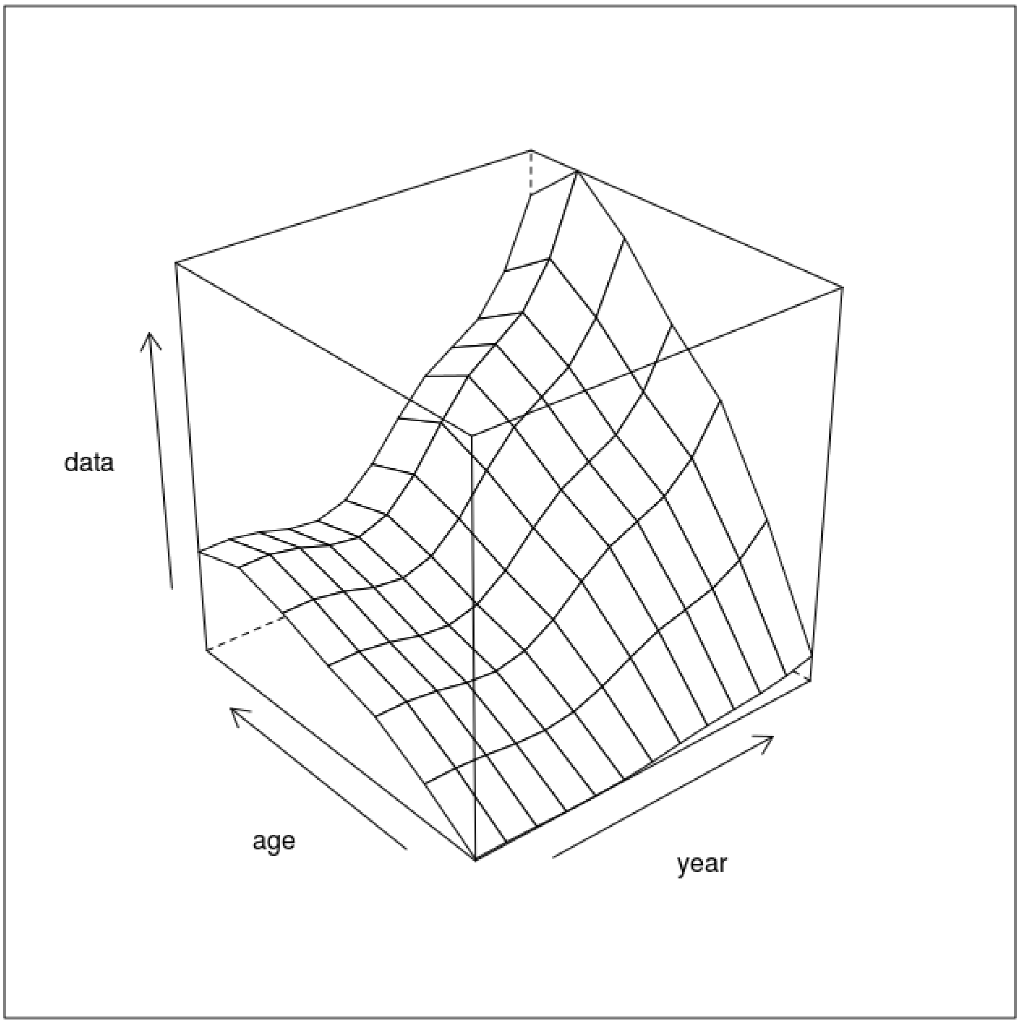


```
pil.fit <- fit
pil.fstk <- stk + fit
pil.fstks <- stk + a4aSCA(stk, ids, fit = "MCMC", fmodel = fmod1a, qmodel = qmod1,
    n1model = n1, srmodel = rmod1, mcmc = mc)
plot(pil.fstks)
```

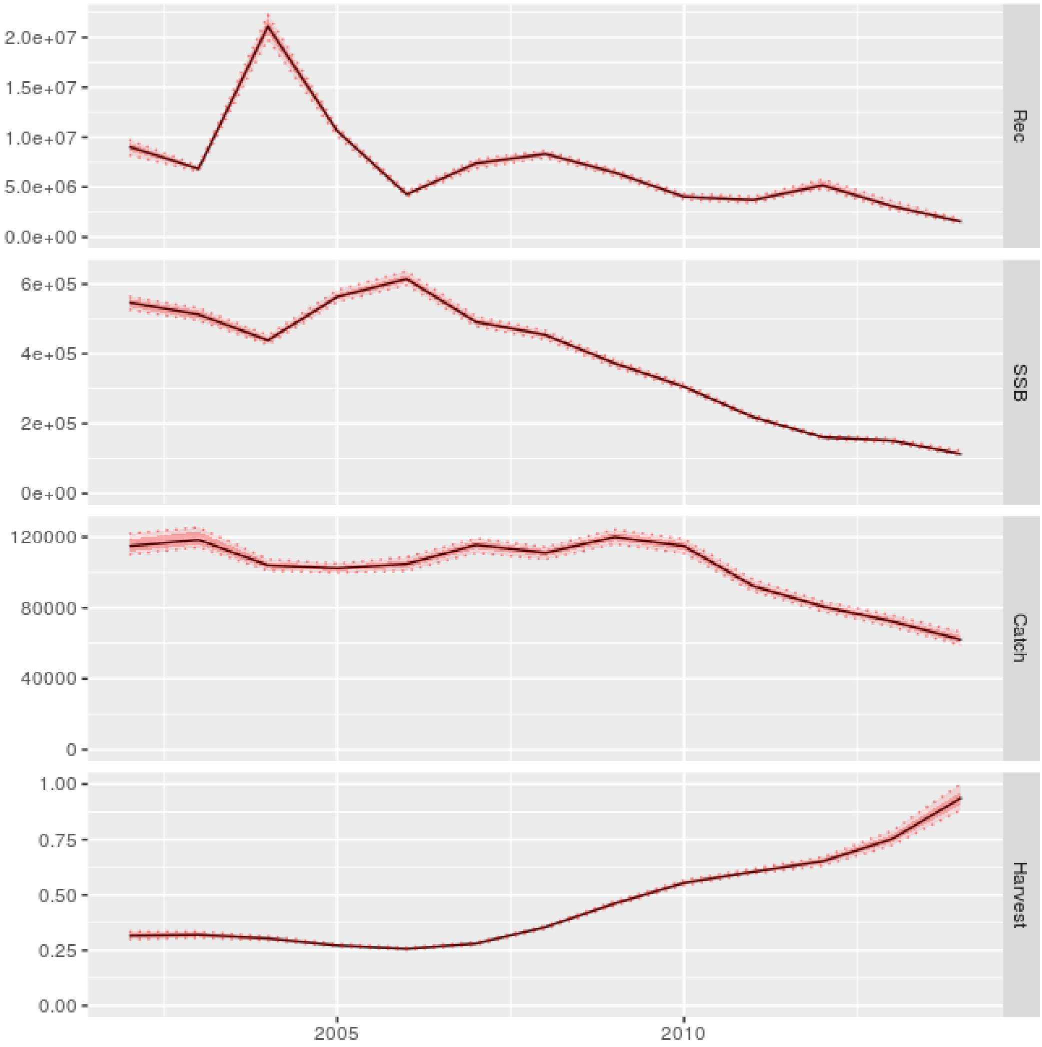

4.2 BB

```
stk <- BB.stk
ids <- BB.idx
fit <- a4aSCA(stk, ids, fmodel = fmod1b, qmodel = qmod2, n1model = n1, srmodel = rmod1)
res <- residuals(fit, stk, ids)
plot(res)
```

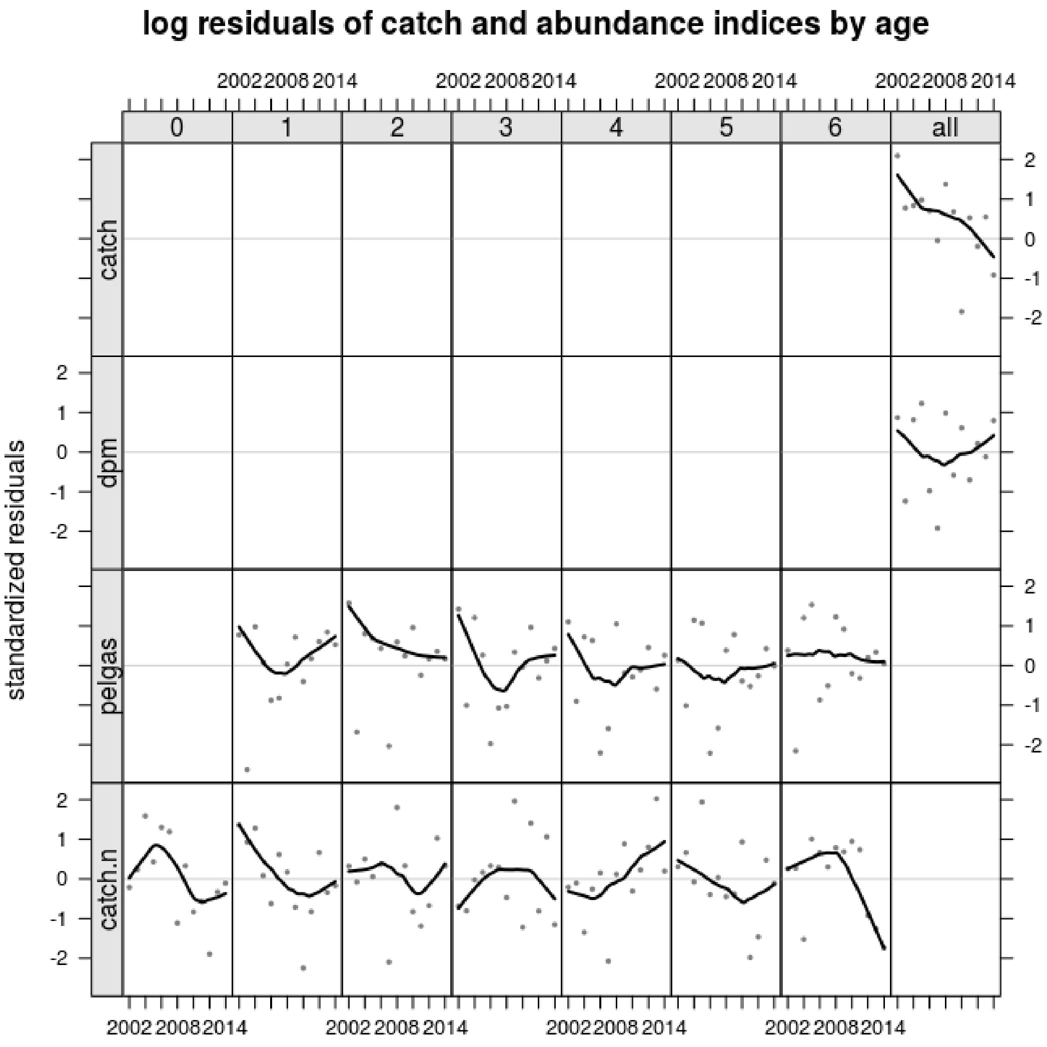


```
wireframe(data ~ year + age, data = harvest(fit))
```

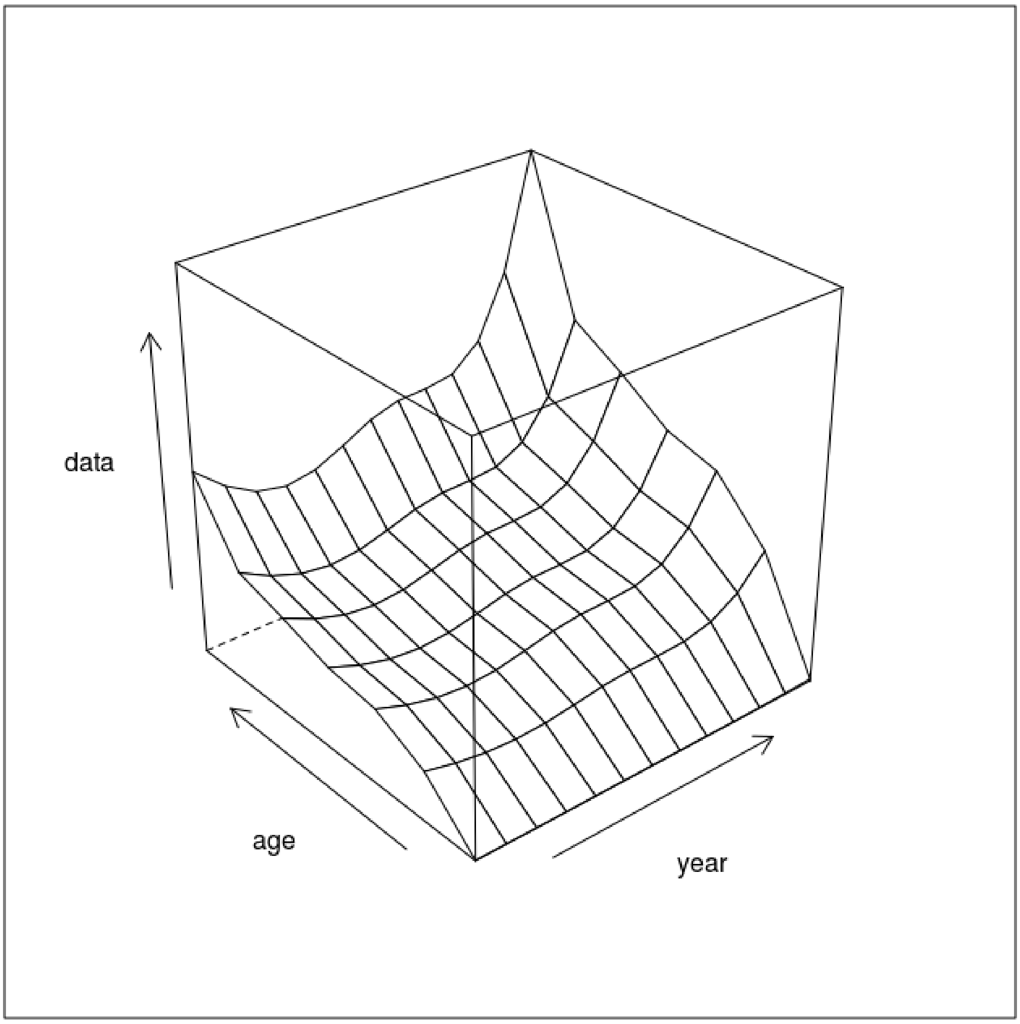


```
pilbb.fit <- fit
pilbb.fstk <- stk + fit
pilbb.fstks <- stk + a4aSCA(stk, ids, fit = "MCMC", fmodel = fmod1b, qmodel = qmod2,
    n1model = n1, srmodel = rmod1, mcmc = mc)
plot(pilbb.fstks)
```

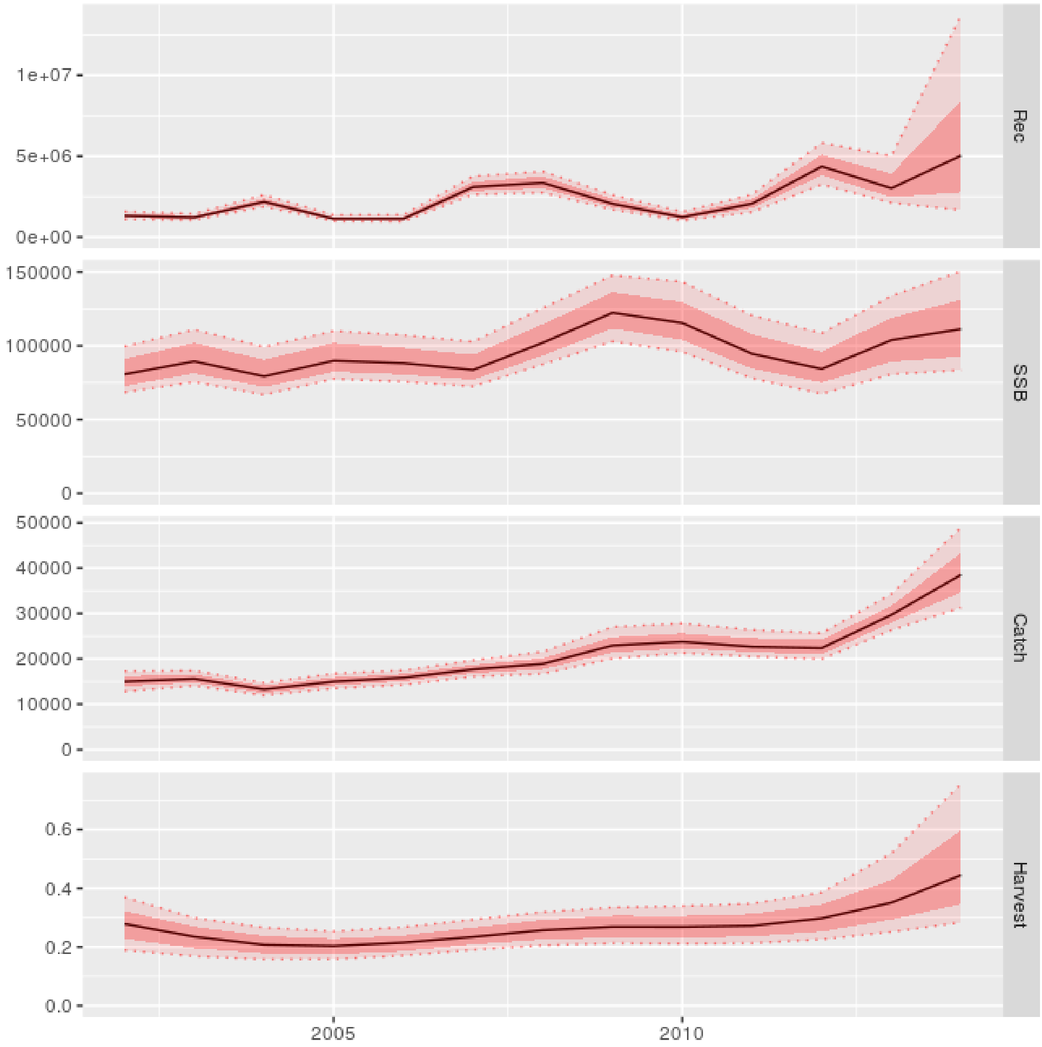

4.3 NW

```
stk <- NW.stk
ids <- NW.idx
fit <- a4aSCA(stk, ids, fmodel = fmod2a, qmodel = qmod2, n1model = n1, srmodel = rmod2)
res <- residuals(fit, stk, ids)
plot(res)
```

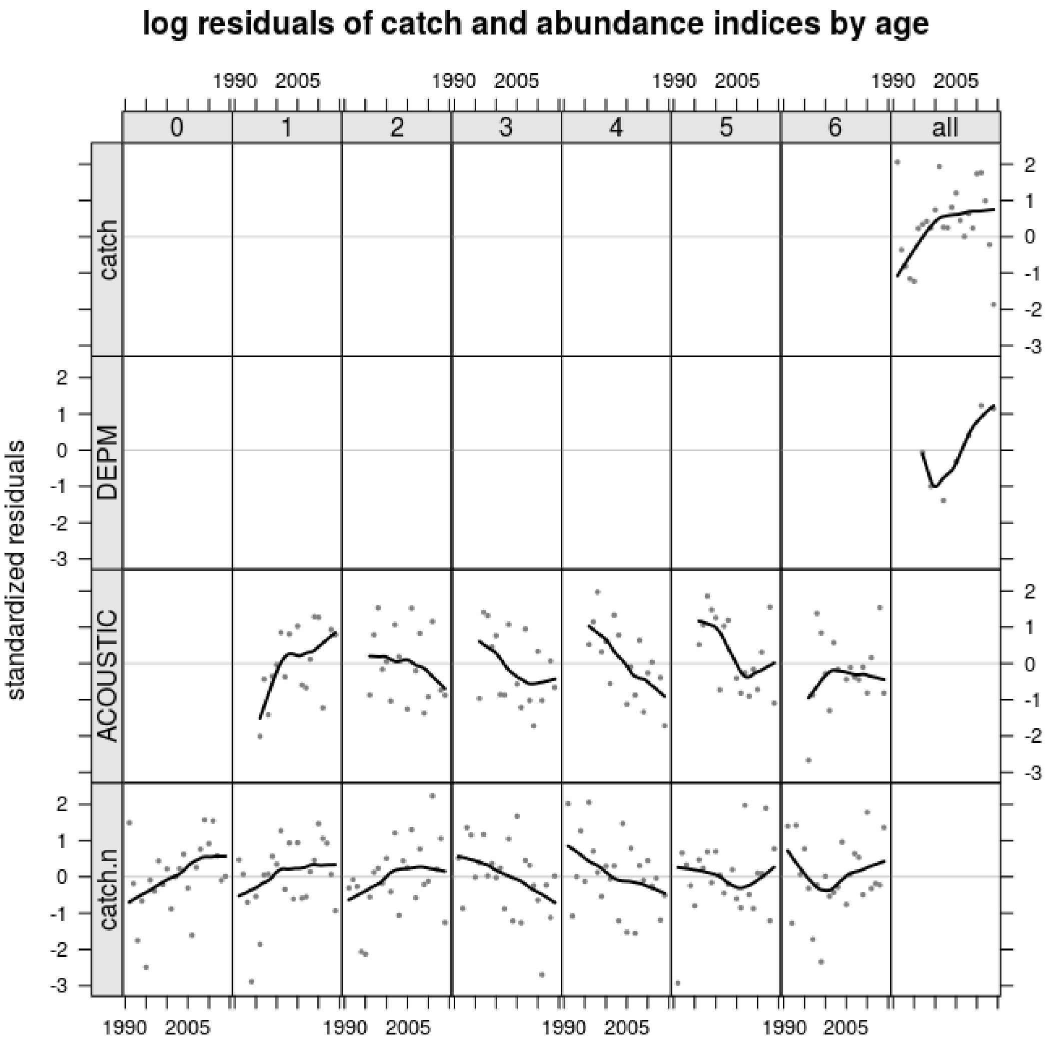


```
wireframe(data ~ year + age, data = harvest(fit))
```

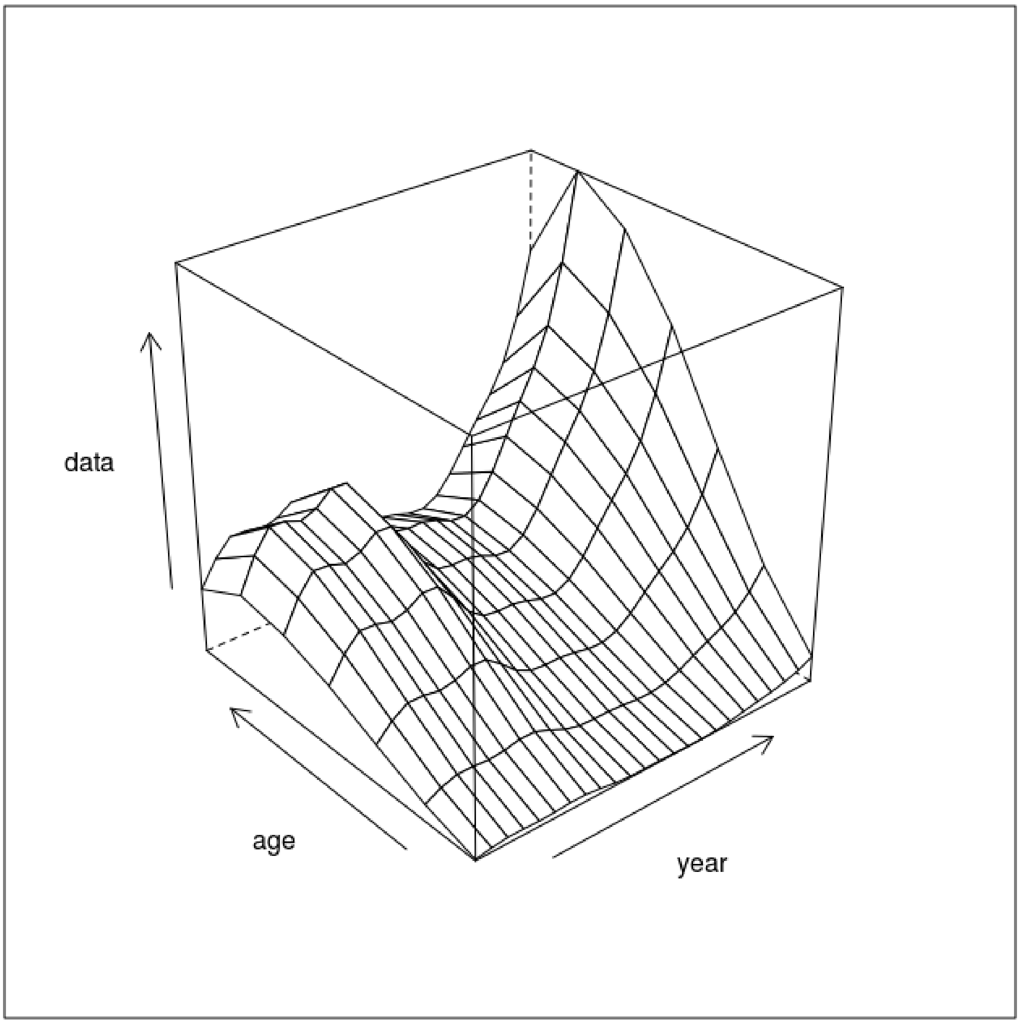


```
pilnw.fit <- fit
pilnw.fstk <- stk + fit
pilnw.fstks <- stk + a4aSCA(stk, ids, fit = "MCMC", fmodel = fmod2a, qmodel = qmod2,
    n1model = n1, srmodel = rmod2, mcmc = mc)
plot(pilnw.fstks)
```

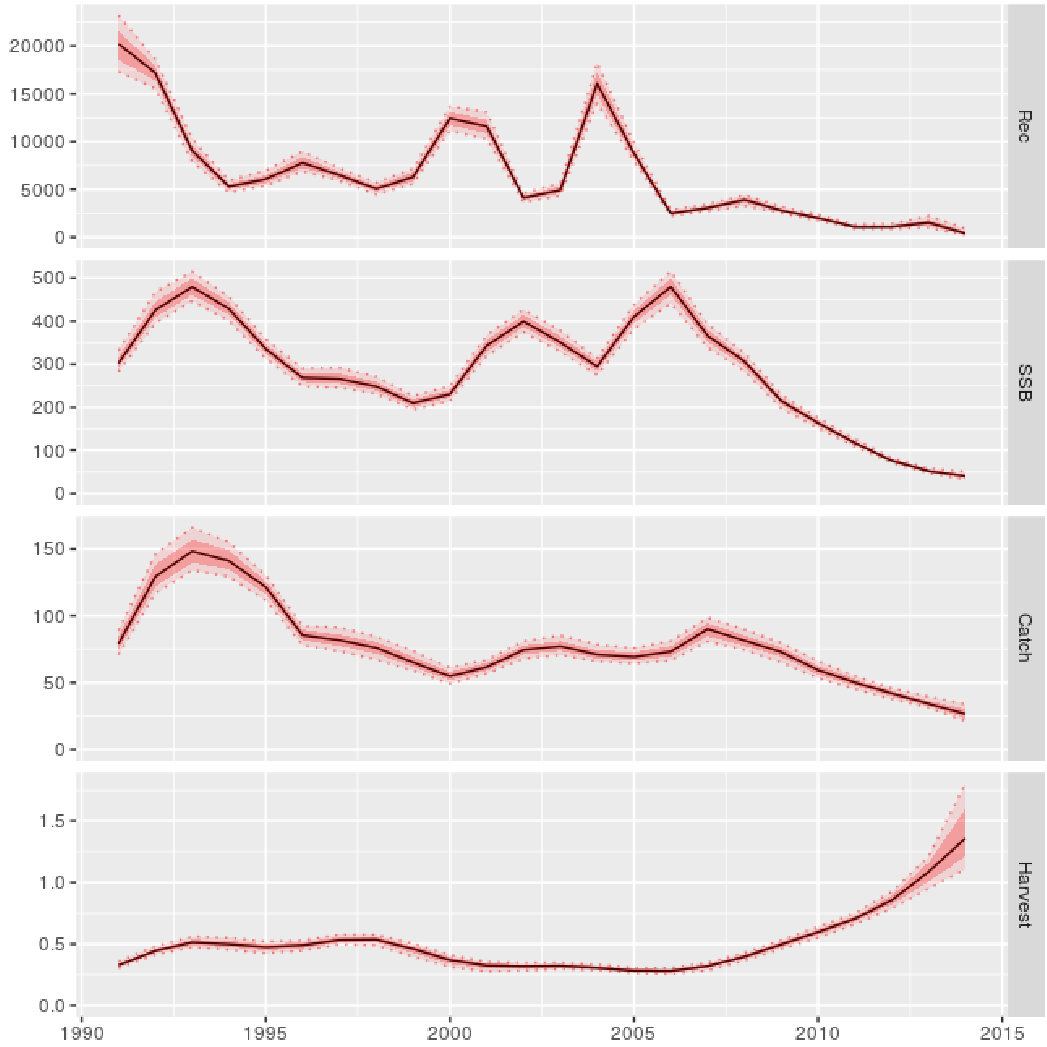

4.4 S

```
stk <- S.stk
ids <- S.idx
fit <- a4aSCA(stk, ids, fmodel = fmod2a, qmodel = qmod2, n1model = n1, srmodel = rmod2)
res <- residuals(fit, stk, ids)
plot(res)
```

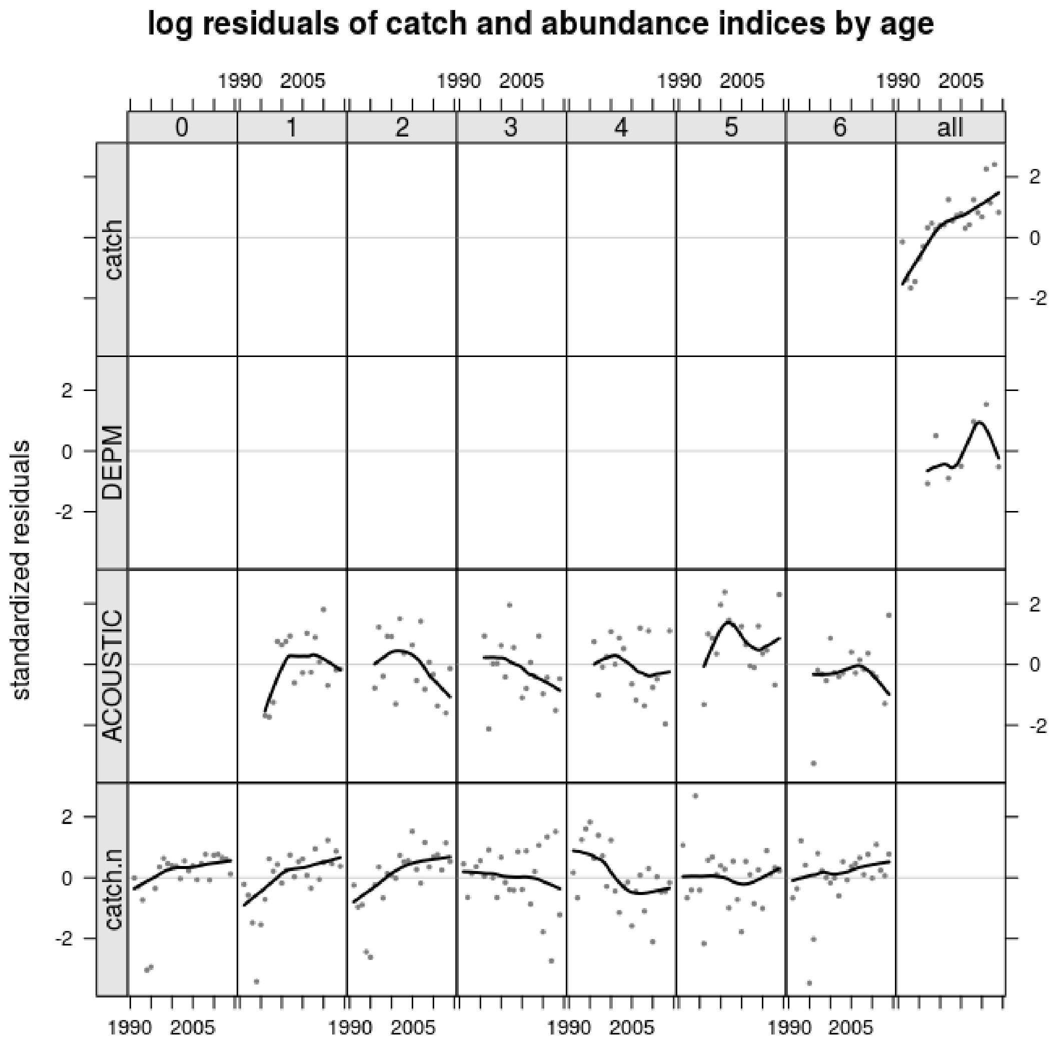


```r
wireframe(data ~ year + age, data = harvest(fit))
```

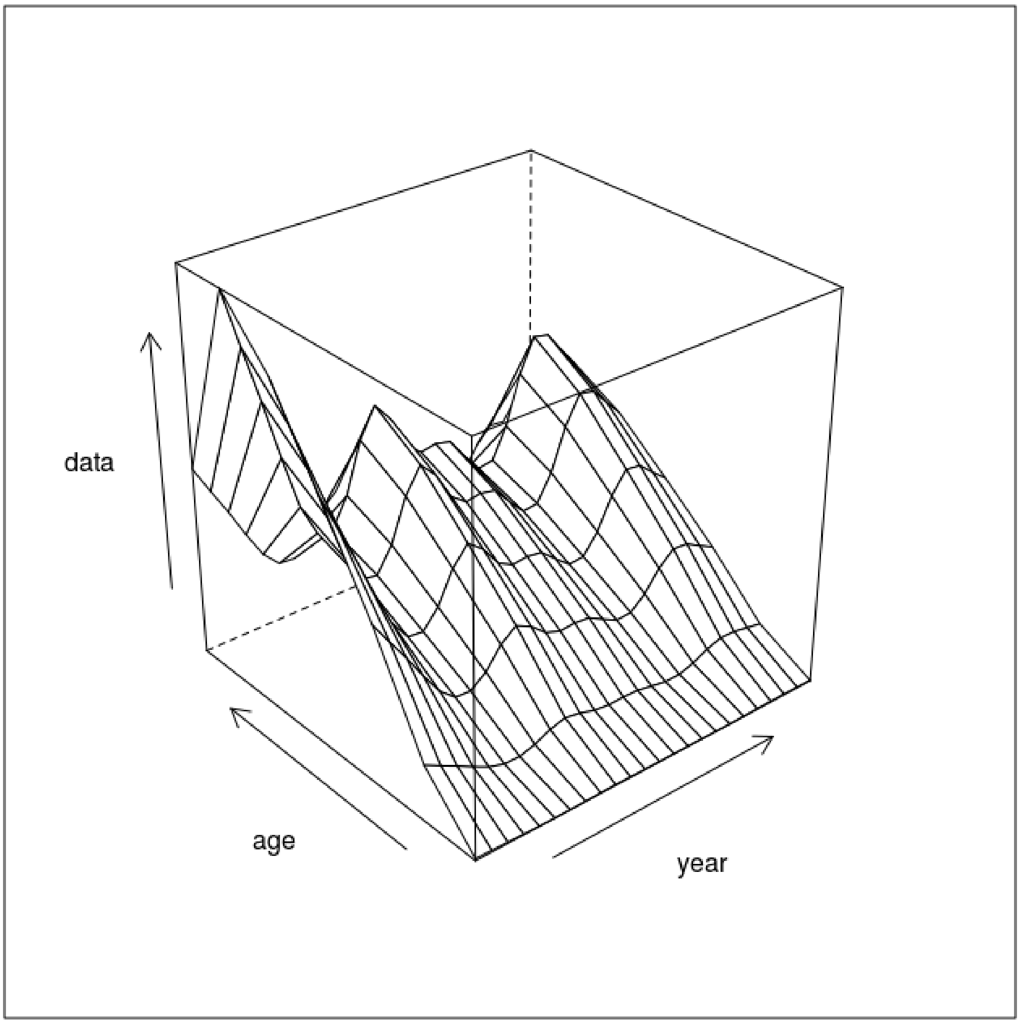


```
pilso.fit <- fit
pilso.fstk <- stk + fit
pilso.fstks <- stk + a4aSCA(stk, ids, fit = "MCMC", fmodel = fmod2a, qmodel = qmod2,
    n1model = n1, srmodel = rmod2, mcmc = mc)
plot(pilso.fstks)
```

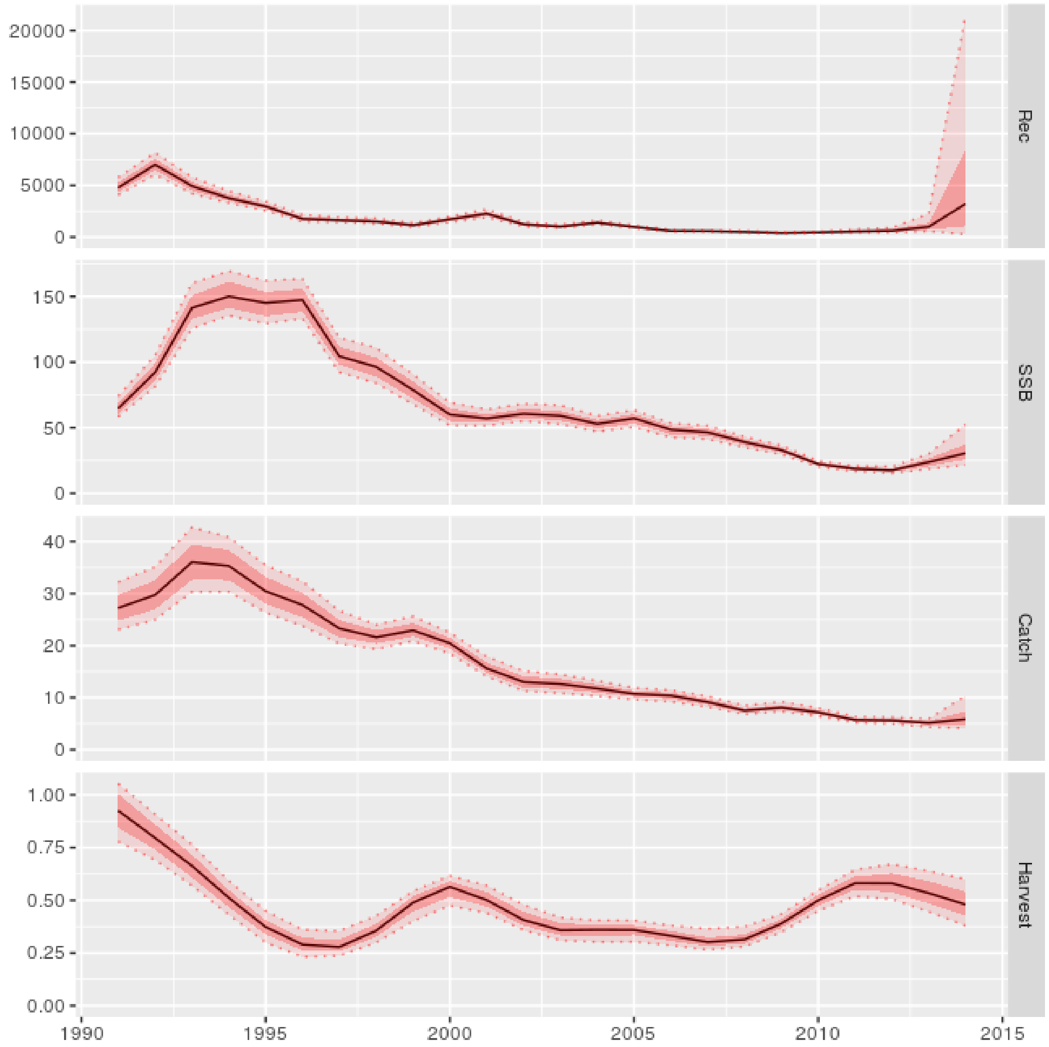

5 Comparing tensor with separable fishing mortalities

```
pil3ssb <- ssb(window(pilnw.fstk, start = 2002)) * 1000 + ssb(window(pilso.fstk,
    start = 2002)) * 1000 + ssb(pilbb.fstk)
ssb.flqs$"sub-populations:separable" <- pil3ssb
xyplot(data ~ year | factor(age), groups = qname, data = lapply(ssb.flqs, iterMedians),
    type = c("g", "l"), auto.key = list(points = FALSE, lines = TRUE, space = "bottom"),
    scales = list(y = list(relation = "free")), main = "SSB", ylab = "", par.settings = pset)
```

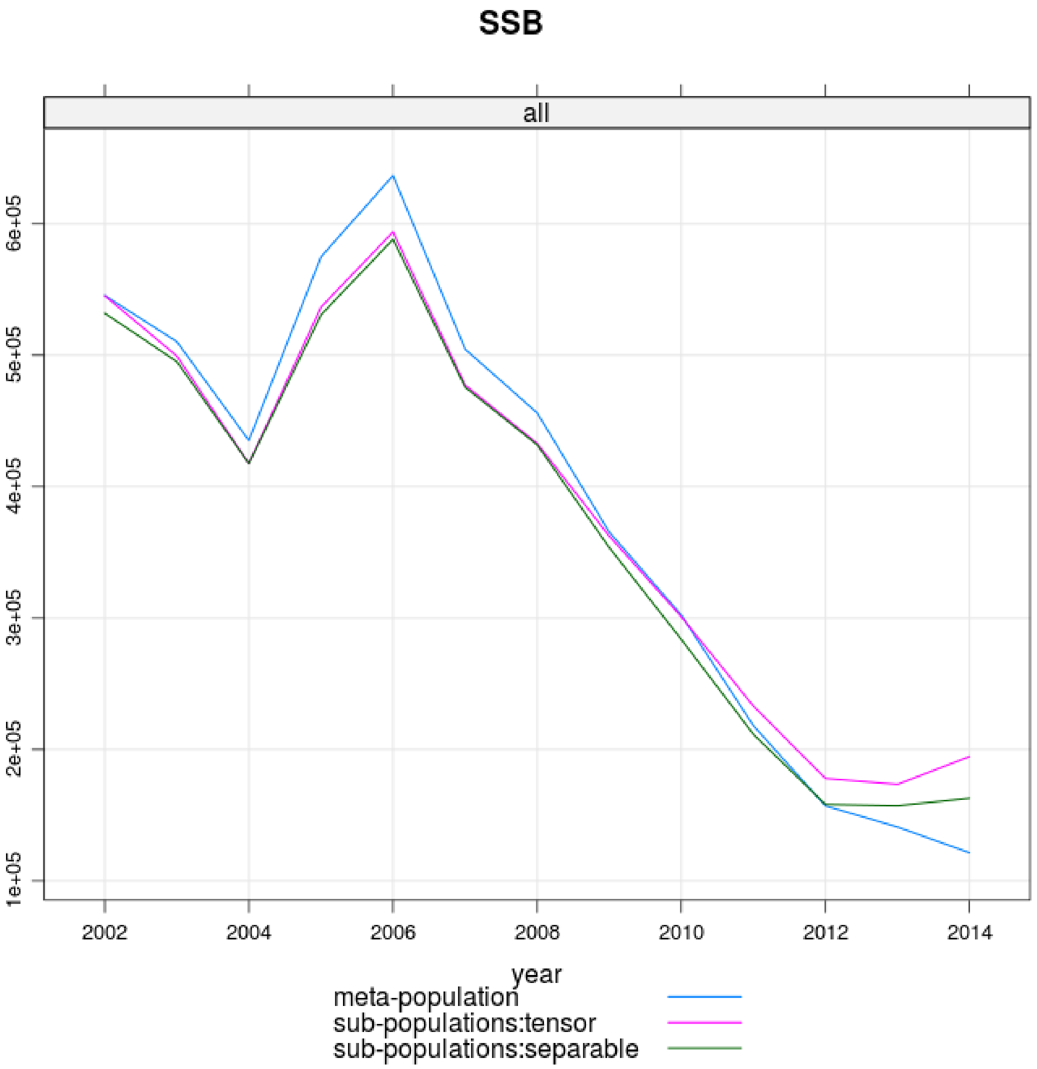


```
pil3n <- stock.n(window(pilnw.fstk, start = 2002)) * 1000 + stock.n(window(pilso.fstk,
    start = 2002)) * 1000 + stock.n(pilbb.fstk)
n.flqs$"sub-populations:separable" <- pil3n
xyplot(data ~ year | factor(age), groups = qname, data = n.flqs, type = c("g",
    "l"), auto.key = list(points = FALSE, lines = TRUE, space = "bottom"), scales = list(y = list(relat
    main = "N", ylab = "", par.settings = pset)
```

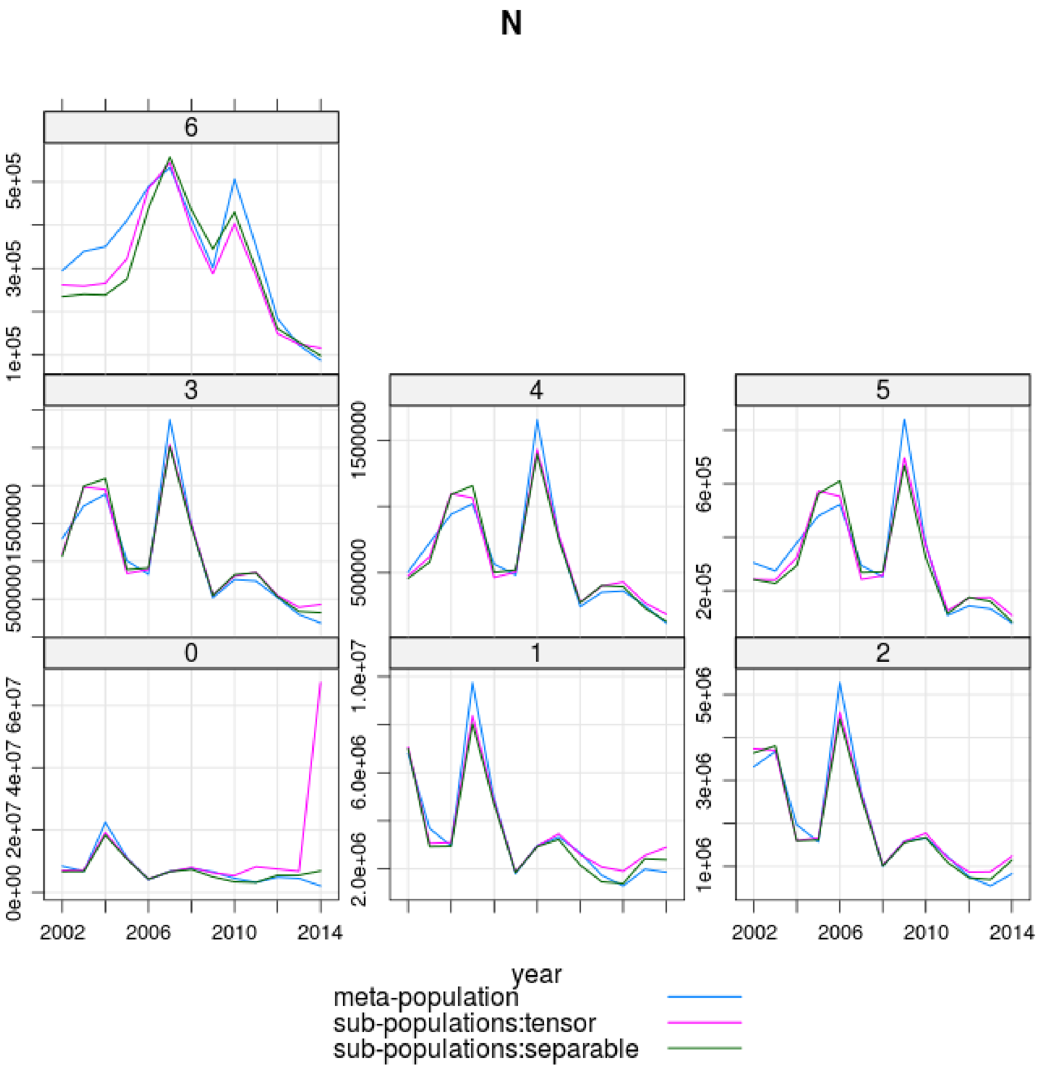


```
wireframe(data ~ age + year | qname, data = as.data.frame(lapply(pil3f, "[",
    ac(0:5))), main = "F tensor", zlab = "F", par.settings = pset)
```

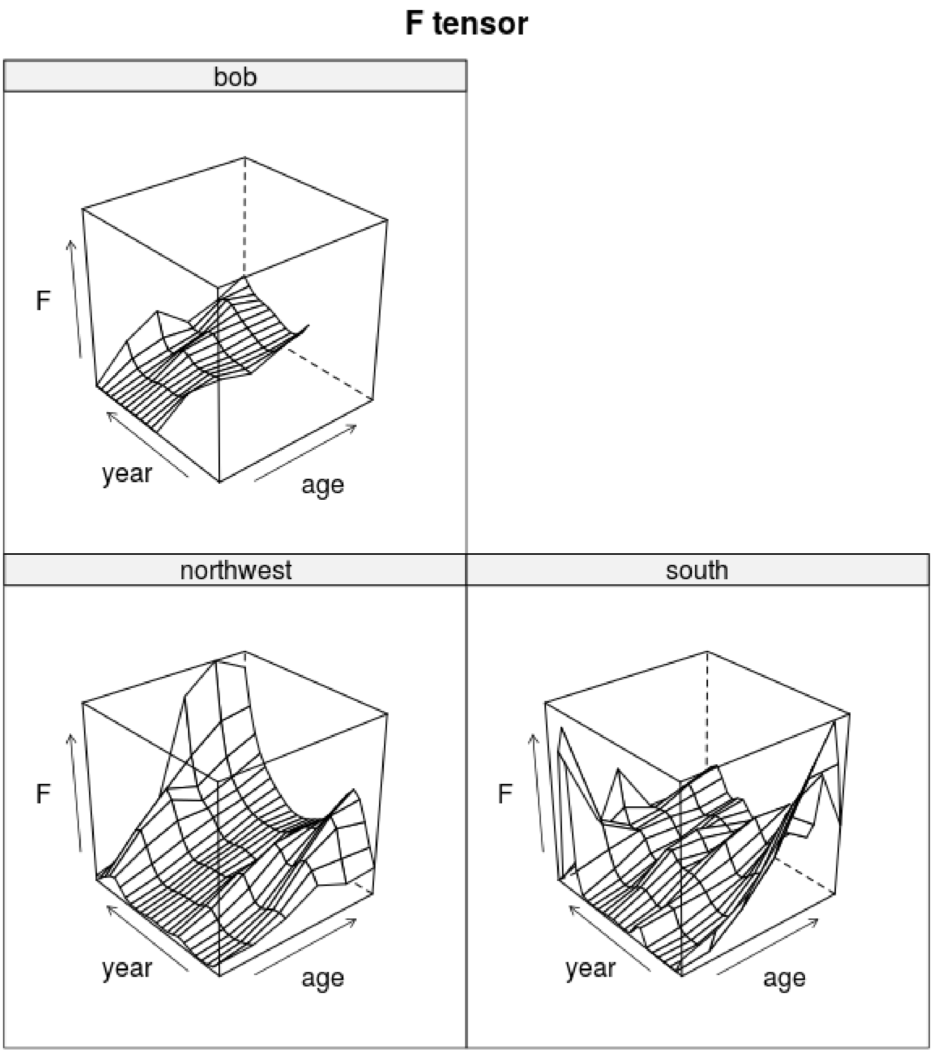


```r
wireframe(data ~ age + year | qname, data = as.data.frame(lapply(pil3fsep, "[",
    ac(0:5))), main = "F separable", zlab = "F", par.settings = pset)
```

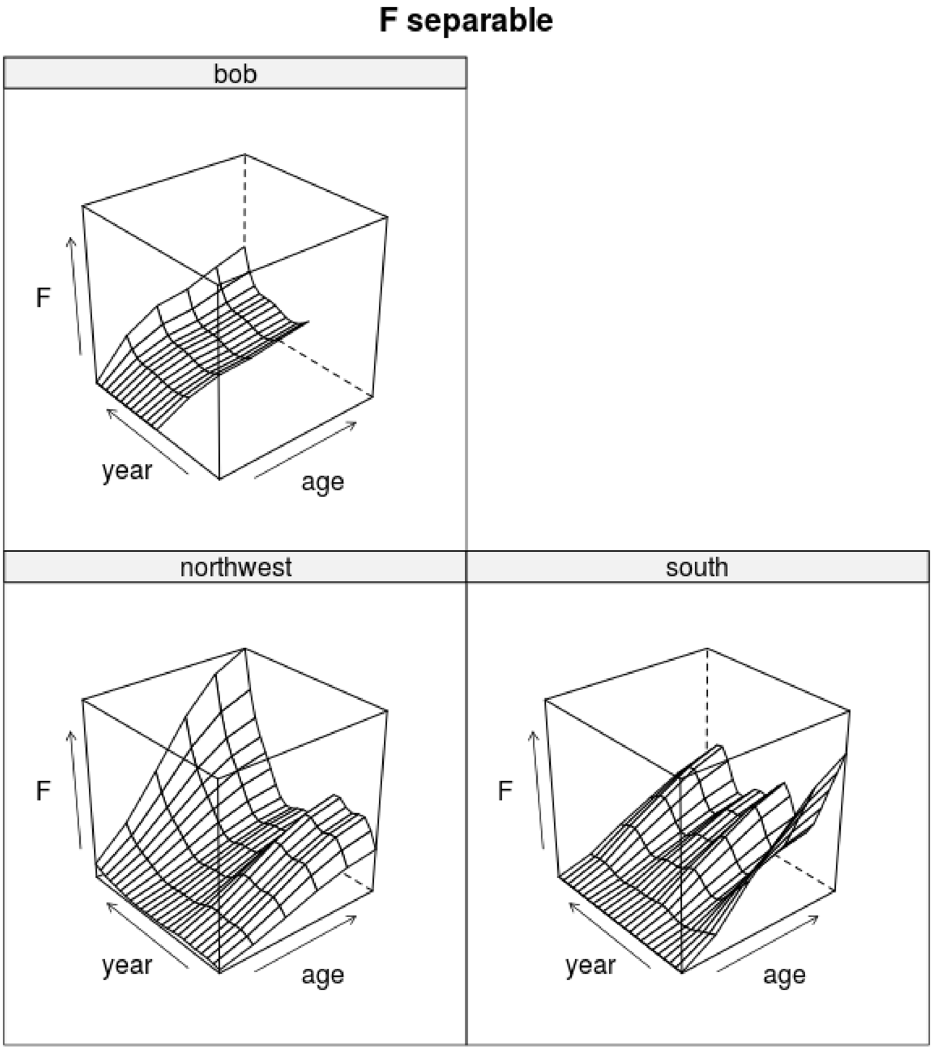
F separable
bob
F
year
age
northwest
F
year
age
south
F
year
age
